# Photoactive preorganized subphthalocyanine-based molecular tweezers for selective complexation of fullerenes†

**DOI:** 10.1039/d0sc00059k

**Published:** 2020-02-28

**Authors:** Germán Zango, Marcel Krug, Swathi Krishna, Víctor Mariñas, Timothy Clark, M. Victoria Martinez-Diaz, Dirk M. Guldi, Tomás Torres

**Affiliations:** Department of Organic Chemistry, Universidad Autónoma de Madrid c/Francisco Tomás y Valiente 7, Cantoblanco 28049 Madrid Spain; Department of Chemistry and Pharmacy, Interdisciplinary Center for Molecular Materials (ICMM), Friedrich-Alexander-Universität Erlangen-Nürnberg Egerlandstrasse 3 Erlangen 91058 Germany dirk.guldi@fau.de; IMDEA-Nanociencia c/Faraday 9, Campus de Cantoblanco 28049 Madrid Spain; Institute for Advanced Research in Chemical Sciences (IAdChem), Universidad Autónoma de Madrid 28049 Madrid Spain

## Abstract

The development of new chromophoric receptors capable of binding curved carbon nanostructures is central to the quest for improved fullerene-based organic photovoltaics. We herein report the synthesis and characterization of a subphthalocyanine-based multicomponent ensemble consisting of two electron-rich SubPc-monomers rigidly attached to the convex surface of an electron-poor SubPc-dimer. Such a unique configuration, especially in terms of the two SubPc-monomers, together with the overall stiffness of the linker, endows the multicomponent system with a well-defined tweezer-like topology to efficiently embrace a fullerene in its inner cavity. The formation of a 1 : 1 complex was demonstrated in a variety of titration studies with either **C60** or **C70**. In solution, the underlying association constants were of the order of 10^5^ M^−1^. Detailed physicochemical experiments revealed a complex scenario of energy- and electron-transfer processes upon photoexcitation in the absence and presence of fullerenes. The close proximity of the fullerenes to the electron-rich SubPcs enables a charge shift from the initially formed reduced SubPc-dimer to either **C60** to **C70**.

## Abstract 2

A tweezer-like subphthalocyanine-based ensemble has been developed for the selective recognition of fullerenes. The physicochemical properties of both the photoactive receptor and its inclusion complexes with fullerenes have been investigated.

## Graphical abstract

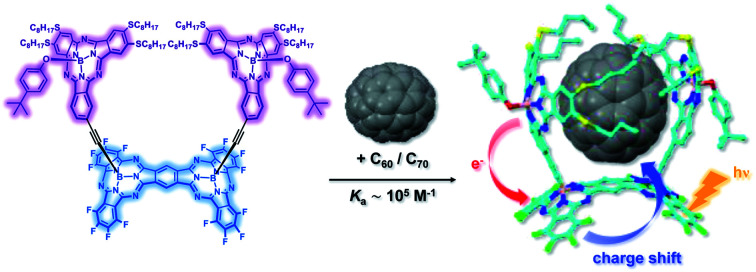

## Introduction

In addition to the outstanding optoelectronic properties that subphthalocyanines (SubPcs)^1,2^ share with other members of the porphyrinoid family, SubPcs are unusual boron compounds with a π-extended bowl-shaped geometry that makes them potential receptors of shape- and size-complementary convex-shaped molecules, such as fullerenes. Fullerene encapsulation within a self-assembled homodimeric SubPc cage was first demonstrated by Claessens and Torres in 2004. They documented the extraction of **C60** from acetone solutions, in which **C60** is barely soluble.^3^ Several additional reports focus on the formation of complexes in which fullerenes are embraced by the concave face of SubPcs in the solid state.^4^ No quantitative solution data were, however, available until titration and Job plot experiments were carried out using UV-Vis and fluorescence techniques.^5^ Shape complementarity alone was shown to be insufficient for a strong fullerene binding by SubPcs. Additionally, structural factors play an important role in the overall binding. The electronic nature of the SubPc and the incorporation of peripheral medium-sized alkyl chains influence both the stoichiometry and the binding strength in solution. Accordingly, while perfluorinated electron-deficient SubPcs do not interact appreciably with fullerenes, unsubstituted SubPcs form 1 : 1 complexes, albeit weakly. Electron-donating hexaalkylthio-substituted SubPcs give jellyfish-like 2 : 1 complexes with **C60** (or **C70**) with binding constants as large as 10^5^ M^−1^. More recently, Nielsen and co-workers described a new design of SubPc-based fullerene receptors. It features two curved SubPcs appropriately pre-organized through a linker to accommodate a spherical fullerene in its pseudo-cavity.^6^ This tweezer-like SubPc dimer forms strong 1 : 1 complexes with **C60** and **C70** with association constants in the order of 10^3^ M^−1^, showing a slightly stronger binding in the case of the latter.

Two SubPc units that share a common benzene give rise to unusual SubPc-fused dimers with unique curved π-extended topologies. They exist as both *syn*- and *anti*-isomers with either hemicircular or S-like shapes, respectively.^7^ The binuclear character of the SubPc dimers provides them with two functionalizable axial positions. In this context, an example of a perfluorinated SubPc *syn*-dimer doubly connected through its axial positions to a fullerene was recently described.^8^ Such a double connection through the convex face of the macrocycle forced the docking of the **C60** at a relatively fixed position. Consequently, through-space interactions between the SubPc-dimer and the electron-accepting **C60** were formed.

**Scheme 1:**
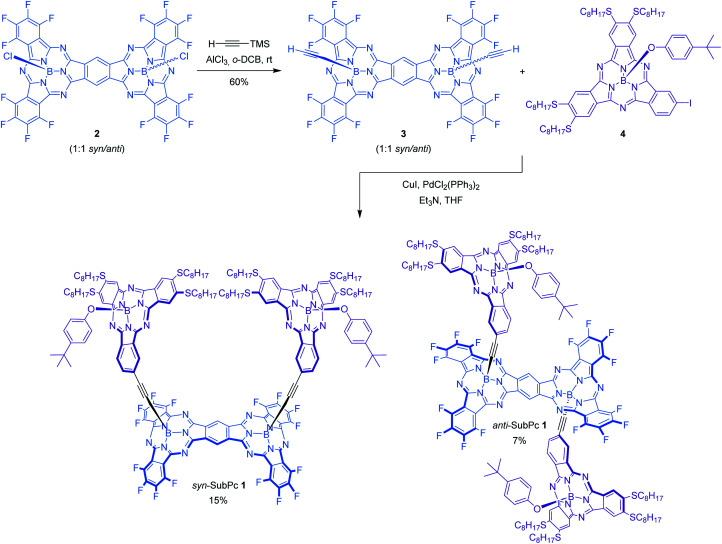
Synthesis of SubPc–SubPc dimer hybrids *syn*-SubPc **1** and *anti*-SubPc **1**.

In this work, a novel photoactive pre-organized molecular SubPc-based receptor *syn*-SubPc **1** (Scheme 1) has been designed and synthesized for the selective recognition of fullerenes.

Its design is based on two electron-donating tetraoctylthio-substituted SubPcs attached through rigid ethynyl spacers to the boron atoms of one of the faces of a SubPc-fused dimer rigid platform, moulding a well-defined cavity. Arranging two topologically and electronically complementary host sites in a pre-organized conformation is likely to reduce the entropy and the deformation associated with the formation of supramolecular assemblies.^6,9^ Besides, the introduction of thioether alkyl chains is expected to strengthen the complexation of fullerenes through additional van der Waals interactions as well as hydrophobic effects.^5^ Axial substitution of the boron atom of the two complexing SubPc monomers with a bulky 4-*tert*-butylphenoxy group was envisioned to enforce the desired face-to-face conformer suitable for fullerene complexation (*vide infra*). In addition, to integrate all SubPcs into single ensembles suited for probing intramolecular energy/electron transfer processes, electron-withdrawing fluorine substituents were introduced at the SubPc dimer. Binding of different fullerenes, namely **C60** and **C70**, has been analyzed by means of UV-vis absorption and fluorescence titrations. Because of the complementary absorption and electronic properties of the constituents of the two SubPc and SubPc fused dimers, *syn*-SubPc **1** is well suited for probing energy/electron transfer processes.^10^

## Results and discussion

### Synthesis and characterization of receptor *syn*-SubPc **1**

The synthesis of *syn*-SubPc **1** was based on the preparation of bis(ethynyl)boron-functionalized dimer-SubPc **3** and subsequent double Sonogashira coupling reaction with unsymmetrical octylthio-substituted monomer-SubPc **4** (Scheme 1). Axial substitution of a 1 : 1 *syn*/*anti* mixture of dimer-SubPc **2** with 5 equivalents of ethynyltrimethylsilane and 4 equivalents of AlCl_3_ yielded **3** as a 1 : 1 *syn*/*anti* mixture in 60% yield. The small size and low polarity of the axial ethynyl substituents precluded the separation of the two topoisomers by chromatographic work-up on silica gel. Indeed, identical ^1^H-NMR and UV-vis features were recorded when each topoisomer was synthesized individually and characterized by starting from isomerically pure *syn*-dimer-SubPc **2** or *anti*-dimer-SubPc **2**. Finally, Pd-catalyzed cross-coupling reaction of **3** with monomer-SubPc **4** led to the formation of *syn*-SubPc **1** and *anti*-SubPc **1**, in 15% and 7% yields, respectively, after purification by column chromatography on silica gel and size-exclusion chromatography.

^1^H-NMR spectra of both topoisomers in CDCl_3_ support the proposed structure (see the ESI, Fig. S3 and S4†), exhibiting several diagnostic singlet chemical shifts around 10.50–10.45 ppm in *syn*-SubPc **1** and 10.27–10.20 ppm in *anti*-SubPc **1**. They correspond to the protons of the central benzene ring of the dimeric unit. The appearance of more than one singlet may indicate the presence of two possible diastereomers (*rac* and *meso*) for each topoisomer. In addition, the signals corresponding to the octylthio-substituted SubPc fragments are shifted upfield significantly with respect to the unsymmetrical TMS-ethynyl-substituted SubPc reference in both *syn*-SubPc **1** and *anti*-SubPc **1**. This is due to the influence of the anisotropic ring current of the aromatic system of the SubPc dimer. The assignment of the two topoisomers to a specific *syn* or *anti* configuration was based on variable temperature ^1^H-NMR experiments in CD_2_Cl_2_ at low temperatures (298–188 K) of each fraction (Fig. S5†).§§While the computationally optimized molecular model of topoisomer *syn*-**1** showed impeding full rotation around single B_Dimer_–(C≡C) and (C≡C)–C_SubPc_ bonds, implying going through an intermediate structural conformation where the axial groups of SubPc units are nearly in contact, in topoisomer *anti*-**1**, the orientation of both axial SubPc units towards opposite sides of the SubPc dimer moiety would not affect the free rotation around the mentioned bonds.

### Computational studies

Molecular structures were pre-optimized using the Dreiding Force Field^11^ and promising conformers were further refined at the B3LYP^12^/def2-SVP^13,14^ level of theory including Grimme's D3 correction with Becke–Johnson damping^15^ to account for dispersion interactions. The calculations were performed with the Gaussian 16 ^16^ software package. *t*-Bu groups and alkyl chains were replaced by hydrogen to reduce computational cost.

**Fig. 1:**
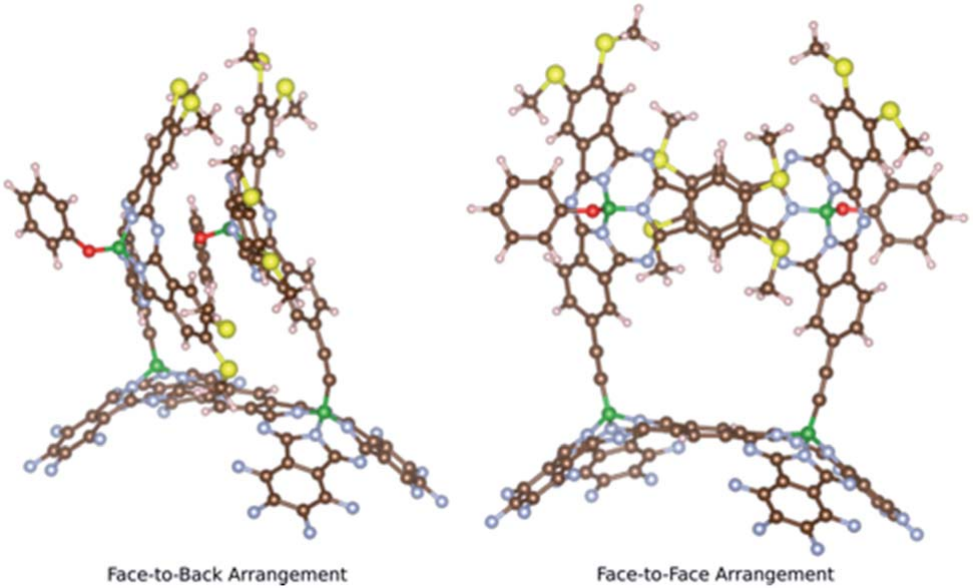
Two computationally optimized structures of *syn*-SubPc **1**.

Two main conformers were found for *syn*-SubPc **1** (Fig. 1); on the one hand, a face-to-back orientation, in which the π-acidic core of one SubPc monomer interacts with the electron-rich phenyl-substituent of the other to yield a slip-stacked arrangement^17,18^ and, on the other hand, a slightly less stable conformer, in which the two SubPc monomers are oriented in a face-to-face geometry.

**Fig. 2:**
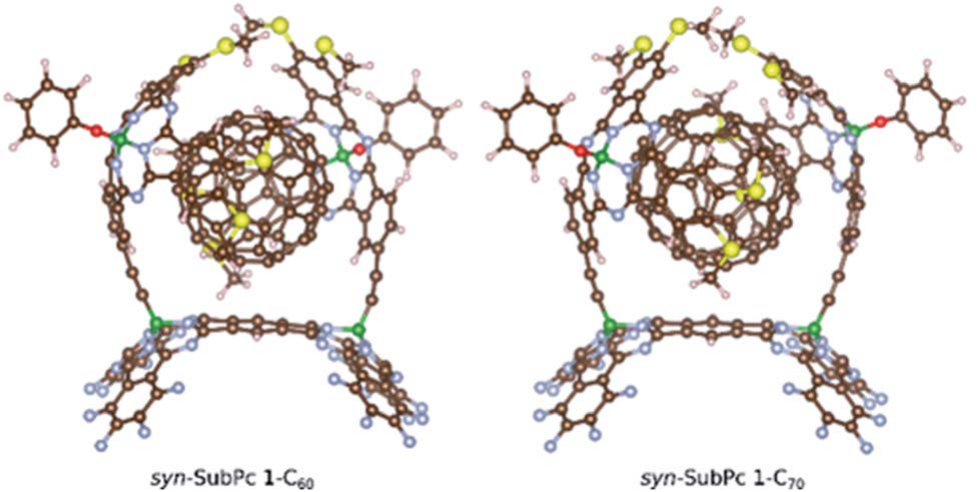
Computed structures of the *syn*-SubPc **1**–fullerene supramolecular complexes.

Furthermore, we optimized the complexes of *syn*-SubPc **1** with **C60** and **C70** (Fig. 2). The two SubPc units orient themselves to form a suitable cavity for the fullerenes. For **C60**/**C70**, the distances between them and the phenyl rings are approximately 3.1 Å, which is in good agreement with previous computational studies.^19^**C60** shows no significant orientational preference, whereas **C70** prefers to maximize the ball-socket contact area by exposing its equatorial region to the SubPcs.^19^

### Steady-state absorption and fluorescence spectroscopy

**Fig. 3:**
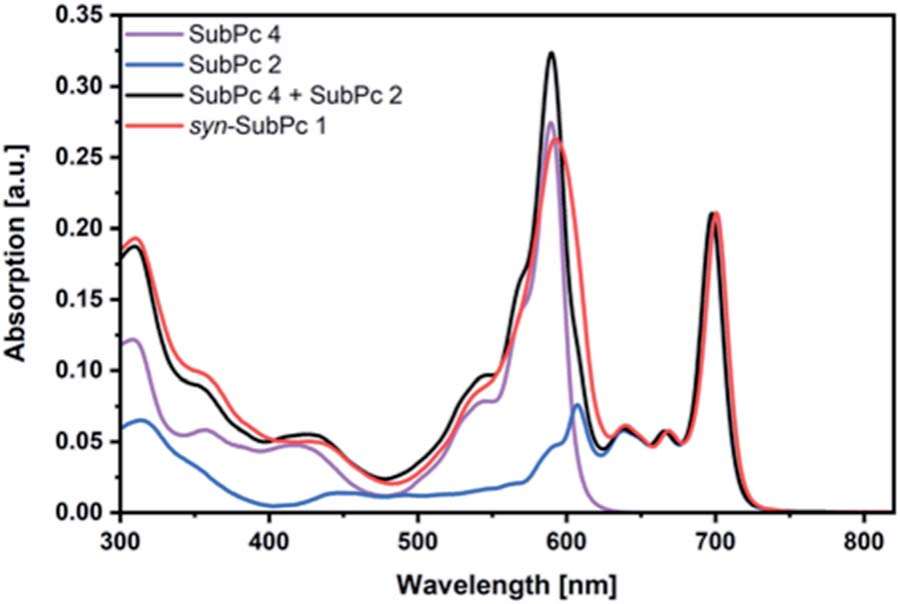
Steady-state absorption spectra of *syn*-SubPc **1** (1.5 × 10^−6^ M), dimer-SubPc **2** (1.5 × 10^−6^ M) and monomer-SubPc **4** (3.0 × 10^−6^ M) in toluene. The black spectrum relates to that of a 1 : 2 mixture of **2** and **4**.

Initially, steady-state absorption spectroscopy of SubPcs **1–4** was carried out in toluene (Fig. 3). Monomer-SubPc **4** features the characteristic B-band absorption at 310 nm and an intense Q-band absorption at 589 nm. Relative to unsubstituted SubPcs, the Q-band absorption is red-shifted due to electronic interactions with the electron-rich sulphur at the periphery.^5,7*b*,20^ Additional absorption features that evolve in the 360 to 430 nm region were attributed to n–π transitions from the lone pair of the sulphur to the π-system of the SubPc.^5,7*b*^ As previously reported,^7*b*^ four Q-band absorptions are noted in the case of dimer-SubPc **2**: 607, 638, 666, and 698 nm. Their presence relates to a loss of degeneracy of the frontier orbitals because the monomer is substituted unsymmetrically in the fused dimer. At first glance, the absorption spectrum of *syn*-SubPc **1** appears to be the linear combination of the individual building blocks. Notably, however, the absorptions of the dimer-subunit are red-shifted by a few nanometers, while the Q-band absorptions of the monomer-subunits are subject to an overall broadening. Changing the solvent polarity only impacts the absorption spectra of *syn*-SubPc **1**, but not that of dimer-SubPc **2** or that of monomer-SubPc **4**. We note slight red-shifted Q-band absorptions of the monomer-subunits and broadened Q-band absorptions of the dimer-subunit on going from toluene to chlorobenzene and benzonitrile (Fig. S6†). Throughout the solvent changes, the Q-band ratios between the monomer- and dimer-subunits increase favouring the latter in the more polar solvents.

**Fig. 4:**
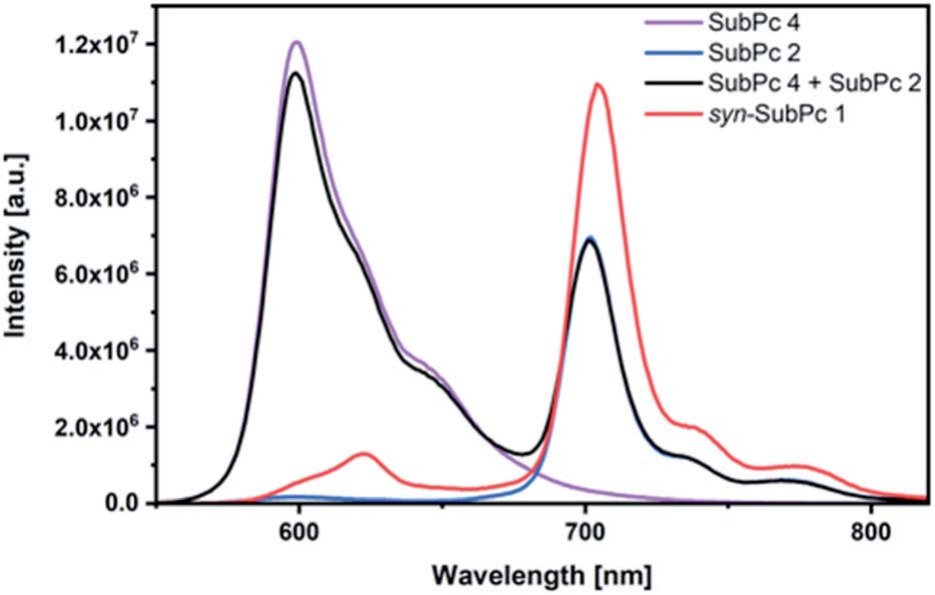
Steady-state fluorescence spectra of *syn*-SubPc **1** (1.5 × 10^−6^ M), dimer-SubPc **2** (1.5 × 10^−6^ M) and monomer-SubPc **4** (3.0 × 10^−6^ M) in toluene. The black spectrum relates to that of a 1 : 2 mixture of **2** and **4**. *λ*_ex_ = 530 nm.

Next, we turned our attention to steady-state fluorescence spectroscopy (Fig. 4). To account for inner filter effects, especially throughout the titrations, we used eqn (1) to correct the fluorescence spectra.^21^1
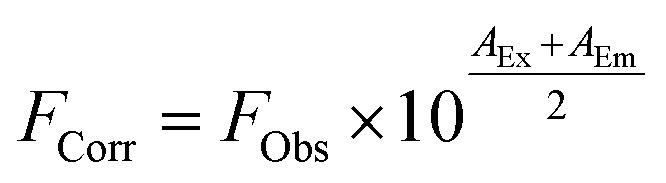


Using 530 nm photoexcitation, monomer-SubPc **4** fluoresces with a maximum at 599 nm and dimer-SubPc **2** at 702 nm. Intriguingly, the fluorescence in the monomer region is dramatically quenched when *syn*-SubPc **1** is photoexcited at 530 nm. In contrast, the fluorescence in the dimer region is intensified compared to the mixture of monomers and dimers. This led us to conclude efficient energy transfer from the monomer-subunit singlet excited state (2.09 eV) to the energetically lower-lying dimer-subunit singlet excited state (1.77 eV).¶¶The singlet excited state energies were derived from the respective low-energy absorption and high-energy fluorescence maxima. Triplet excited state energies were estimated to be 1.41 and 0.80 eV for monomer-SubPc **4** and dimer-SubPc **2**, respectively, from the phosphorescence spectra in a MeTHF glass matrix (Fig. S8 and S9†).

While the fluorescence quantum yields (Table 1) of monomer-SubPc **4** and dimer-SubPc **2** are negligibly affected by the solvent polarity, *syn*-SubPc **1** is barely fluorescent in chlorobenzene and benzonitrile (Fig. S7†). This suggests charge transfer as a non-radiative deactivation pathway, especially in polar solvents.

**Table:** Fluorescence quantum yields of monomer-SubPc **4**, dimer-SubPc **2**, and *syn*-SubPc **1** in toluene, chlorobenzene, and benzonitrile. The fluorescence of *syn*-SubPc **1** after 550 nm excitation is dual with contributions stemming from a monomeric and a dimeric regime

Compound	Excitation	Toluene	PhCl	PhCN
**4**	550 nm^a^	0.13	0.12	0.12
**2**	640 nm^b^	0.29	0.27	0.22
*syn*-SubPc **1**	550 nm^c^	0.01/0.11	0.01/<0.01	0.01/<0.01
	640 nm^b^	0.04	<0.01	<0.01

a *Vs.* F_12_SubPc in toluene (0.40).^22^

b *Vs.* ZnPc in toluene (0.34).^23^

c *Vs.* F_12_SubPc in toluene (0.40)^22^/SubPc **2** in toluene (0.29).

### Complexation experiments with **C60** and **C70**

**Fig. 5:**
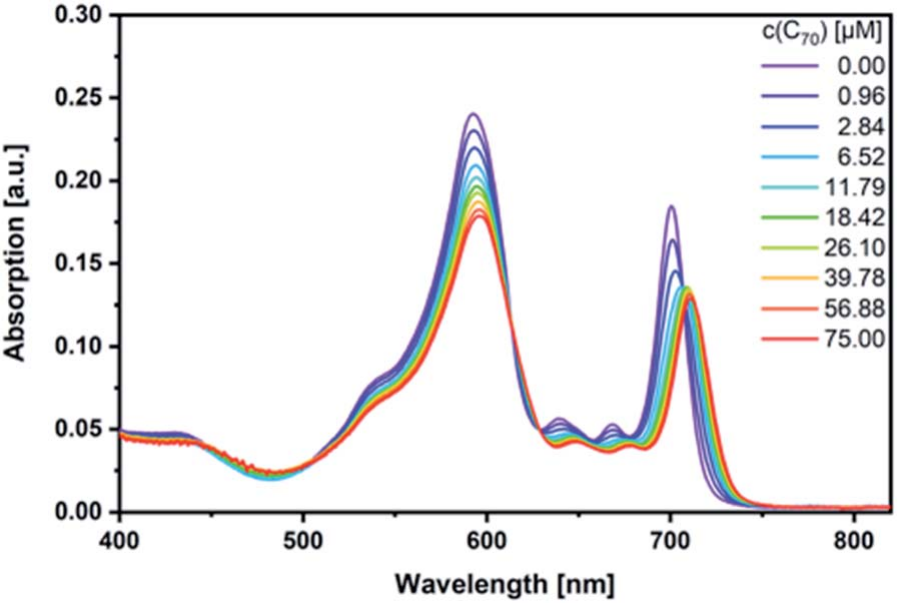
Steady-state absorption spectra of *syn*-SubPc **1** (1.5 × 10^−6^ M) in toluene with increasing amount of **C70** (0–75 μM).

Next, fullerene complexation was tested with the different SubPcs. Despite the fact that complexes between fullerenes and electron-rich SubPcs have been previously reported,^5,24^ we failed to observe any interactions between monomer-SubPc **4** and **C60** or **C70** under our experimental conditions.||||It should be annotated that a minute decrease of the host fluorescence was observed. However, such quenching most probably stems from inner filter effects, which cannot be fully accounted by the correction method we applied in this study.^41^ Similarly, no evidence was found for any interactions between dimer-SubPc **2** and **C60** or **C70**. A likely rationale is the electron accepting nature of both dimer-SubPc **2** and fullerenes.|| A completely different picture was, however, found when *syn*-SubPc **1** was titrated with **C60** and **C70**. Significant changes in the absorption spectrum suggest that electronic communication exists already in the ground state. For example, we noted for **C70** a shift of the monomer-subunit Q-band absorption from 593 to 596 nm (Fig. 5) and an intensity decrease of around 30%. The presence of **C70** has an even stronger impact on the Q-band absorption of the dimer-subunit: a 10 nm bathochromic shift and an intensity decrease.

Similar, albeit less pronounced observations are made with **C60** rather than **C70** (Fig. S11†). From the overall decrease in absorption, we derived binding constants of 3.1 × 10^4^ M^−1^ for **C60** (Fig. S12†) and 1.7 × 10^5^ M^−1^ for **C70** (Fig. S13†) using a non-linear 1 : 1 binding model.^25,26^

**Fig. 6:**
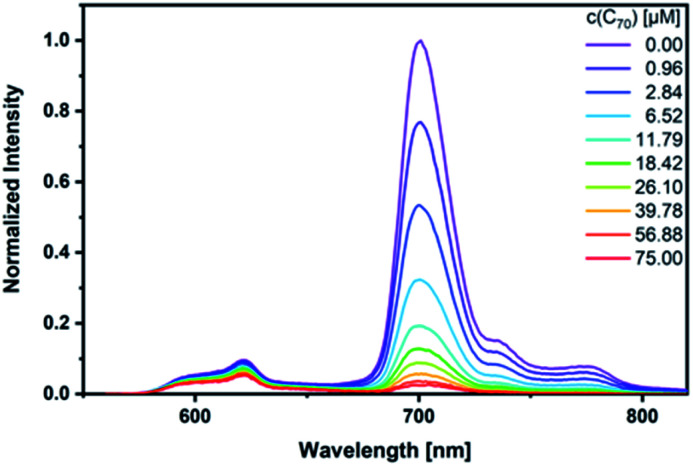
Steady-state fluorescence spectra of *syn*-SubPc **1** (1.5 × 10^−6^ M) after 440 nm excitation in toluene with increasing amount of **C70** (0–75 μM).

**Fig. 7:**
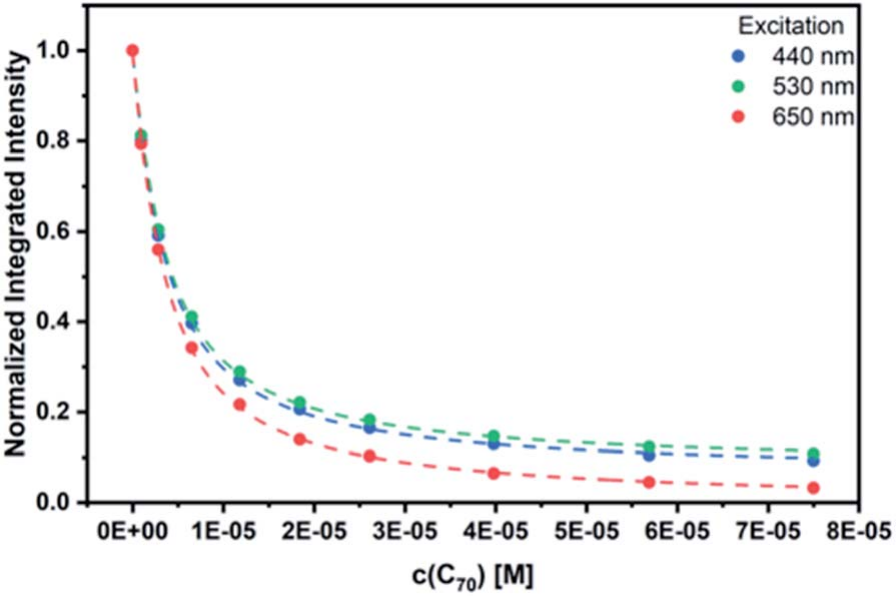
Binding isotherms obtained during the titration of *syn*-SubPc **1** with **C70** by means of steady-state fluorescence spectroscopy.

As a complement, excited-state interactions were characterized by fluorescence measurements using 440, 530, and 650 nm excitation wavelengths. **C60** (Fig. S14†) and/or **C70** (Fig. 6) concentrations as low as 0.96 μM result already in appreciable *syn*-SubPc **1** fluorescence quenching. In excellent agreement with the absorption assays, binding constants of 3.0 × 10^4^ M^−1^ (Fig. S15†) and 3.3 × 10^5^ M^−1^ were determined (Fig. 7) with **C60** and **C70**, respectively.

Job's method of continuous variation^27^ was used to verify the formation of 1 : 1 complexes. For *syn*-SubPc **1–C70**, the intensity of the 715 nm absorption as a function of molar ratio resembles a perfect parabola with a maximum at 0.5 (Fig. S17†). In the case of *syn*-SubPc **1–C60**, the underlying absorption changes were not sufficient to construct a reasonable Job plot. Instead, we used the overall decrease of the dimer-subunit fluorescence upon 640 nm excitation; again, a maximum at 0.5 was found (Fig. S16†). In summary, we conclude that the unique topology of *syn*-SubPc **1** is essential for efficient **C60** or **C70** binding.

### Electrochemistry and charge separated state energies

To evaluate the thermodynamic driving force for possible electron transfer in *syn*-SubPc **1–C60**/*syn*-SubPc **1–C70**, we recorded square-wave voltammograms (Table 2) in *o*-dichlorobenzene.

Reductions and oxidations of monomer-SubPc **4**, dimer-SubPc **2**, and *syn*-SubPc **1**, **C60**, and **C70** obtained by square wave voltammetry in *o*-dichlorobenzene. M: monomer-subunit; D: dimer-subunit

**Table:** 

	*E* ^M^_Red_ [V]	*E* ^M^_Ox_ [V]	*E* ^D^_Red_ [V]	*E* ^D^_Ox_ [V]
**4**	−1.53	0.54	—	—
**2**	—	—	−1.01	0.75
*syn*-SubPc **1**	−1.69	0.55	−1.03	0.76

**Table:** 

	*E* ^Fullerene^_Red_ [V]	*E* ^Fullerene^_Ox_ [V]
**C60**	−1.10	1.30
**C70**	−1.06	1.24

With the oxidation and reduction potentials on hand, we calculated the charge separated state energies using Weller eqn (2).^28^2
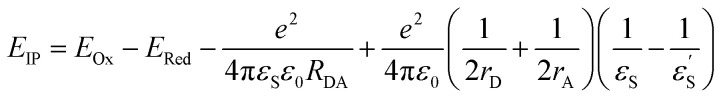
here, *E*_Ox_ and *E*_Red_ refer to the oxidation and reduction potentials, respectively, in the solvent with a dielectric constant 
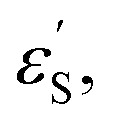
 and *ε*_S_ is the dielectric constant of the solvent used for the time-resolved measurement. The spherical radii of the electron donor *r*_D_ and acceptor *r*_A_ are determined from the DFT calculations to be 5.9 Å for the SubPc-monomer (SubPc_M_), 9.3 Å for the SubPc-fused dimer (SubPc_D_), 4.3 Å for **C60**, and 4.7 Å for **C70**. The center-to-center electron donor–acceptor distance *r*_DA_ = 11.7 Å in *syn*-SubPc **1** were calculated as a mean value from the two conformers. In *syn*-SubPc **1–C60**/*syn*-SubPc **1–C70**, the mean electron donor–acceptor distances (*r*_DA_) are 5.7 and 6.0 Å, respectively. All of the calculated charge separated state energies are listed in Table 3. Please note that the charge separated state energies for *syn*-SubPc **1–C60**/*syn*-SubPc **1–C70** should be treated with caution, since the Weller equation is based on the assumption that the ion pairs are solvent separated. For close contact ion pairs, such as at the van-der-Waals limits of ≈3 Å, this assumption fails and appreciable deviations are expected.^29,30^ Nevertheless, these values are used in the following as a rough first-order approximation for the charge separated states.

**Table:** Charge separated state energies in toluene, chlorobenzene, and benzonitrile estimated through the Weller equation

Solvent	SubPc_M_˙^+^–SubPc_D_˙^−^	SubPc_M_˙^+^–**C60**˙^−^	SubPc_M_˙^+^–**C70**˙^−^
Toluene	1.70 eV	1.51 eV	1.53 eV
PhCl	1.52 eV	1.42 eV	1.41 eV
PhCN	1.41 eV	1.37 eV	1.34 eV

### Time correlated single photon counting

**Fig. 8:**
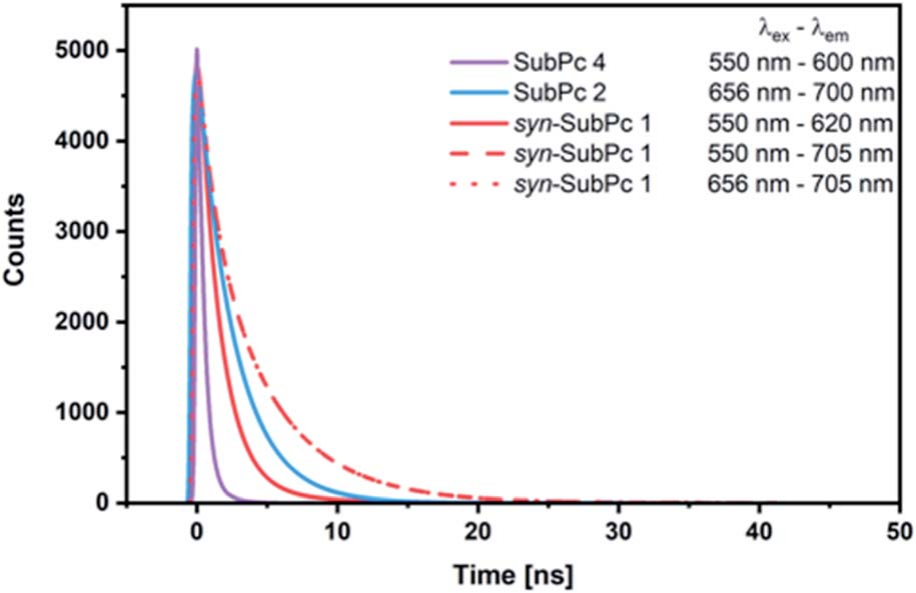
Fits of fluorescence decays obtained by the time correlated single photon counting method of monomer-SubPc **4** (1.5 × 10^−6^ M), dimer-SubPc **2** (1.5 × 10^−6^ M), and *syn*-SubPc **1** (1.5 × 10^−6^ M) in argon purged toluene. The excitation and emission wavelengths were as given in the legend.

Our time-resolved excited-state investigations started with time correlated single photon counting (TCSPC) experiments (Fig. 8). Monomer-SubPc **4** was excited at 550 nm and the corresponding fluorescence was measured at 600 nm. Bi-exponential decays with lifetimes of (0.4 ± 3.0 × 10^−3^) and (1.1 ± 1.4 × 10^−2^) ns, which turned out to be solvent independent, were the best fits to the raw data.^31^ Singlet excited state lifetimes of SubPcs are, however, typically in the range of 2.1 ns.^32,33^ The presence of thioethers in monomer-SubPc **4** exerts a noticeable shortening of the singlet excited state lifetimes;^5,20^ as a heavy atom effect enhances the inter-system crossing. The additional iodine substituent in the periphery is likely to amplify the aforementioned effects and, in turn, shortens the singlet excited state lifetime even further to a value of 0.4 ns. For dimer-SubPc **2**, the solvent independent fluorescence decay at 700 nm upon 656 nm laser excitation is strictly mono-exponential. Here, a singlet excited state lifetime of (2.6 ± 2.4 × 10^−3^) ns is in sound agreement with the literature.^8^ 550 nm photoexcitation of *syn*-SubPc **1** in toluene yields a bi-exponential decay with lifetimes of (1.5 ± 3.9 × 10^−3^) and (4.8 ± 5.1 × 10^−2^) ns. From wavelength-dependent analyses, we conclude that the 1.5 ns lifetime component relates to that of the monomer-subunit, and the 4.8 ns one to that of the dimer-subunit. Using 656 nm as the excitation wavelength gave similar results.

### Transient-absorption pump-probe spectroscopy

Deactivation pathways after photoexcitation were examined by femtosecond and nanosecond transient-absorption pump-probe experiments. In particular, 550 nm laser excitation of monomer-SubPc **4** (Fig. S18†) in toluene leads to an intense ground-state bleaching at 595 nm, which matches the features seen in the steady-state absorption spectrum. In addition, we noted singlet–singlet absorption maxima at 487 and 684 nm.

On a timescale of 0.5 ns, intersystem crossing gives rise to a long-lived triplet excited state with triplet–triplet absorption maxima at 491 and 635 nm. No appreciable changes are noted for chlorobenzene or benzonitrile (Fig. S19 and S20†). The raw data were analyzed by means of global target analysis (Fig. S30–S32†) neglecting, however, any internal conversion due to the rigidity of monomer-SubPc **4**.^34^ In this context, the fluorescence quantum yields were used to calculate the fluorescence rate constants as 1.9 × 10^8^, 1.9 × 10^8^, and 2.1 × 10^8^ s^−1^ and the intersystem crossing rates as 1.3 × 10^9^, 1.4 × 10^9^, and 1.6 × 10^9^ s^−1^ in toluene, chlorobenzene, and benzonitrile, respectively.

Directly after the 656 nm laser excitation of dimer-SubPc **2** in toluene (Fig. S21†), strong ground state bleaching was noted at 611, 641, and 706 nm. In the nIR region, the broad and dominant singlet–singlet absorption is centered at 931 nm. As time progresses, intersystem crossing sets in and leads to the interconversion into the triplet excited state with its absorption maximum at 1059 nm. Essentially similar features were observed in chlorobenzene and benzonitrile (Fig. S22 and S23†). With the help of global target analysis (Fig. S33–S35†), we corroborated that, upon photoexcitation, a fast relaxation process yields the singlet excited state. Considering that dimer-SubPc **2** is rigid, we postulate that internal conversion from a higher-lying singlet excited state plays only a minor role.^34^ Major deactivation pathways of the singlet excited state are fluorescence and intersystem crossing. In light of this, we used the fluorescence quantum yields to determine 1.2 × 10^8^, 1.1 × 10^8^, and 9.2 × 10^7^ s^−1^ as the fluorescence rate constants and 2.9 × 10^8^, 3.1 × 10^8^, and 3.3 × 10^8^ s^−1^ as the intersystem-crossing rate constants in toluene, chlorobenzene, and benzonitrile, respectively.

**Fig. 9:**
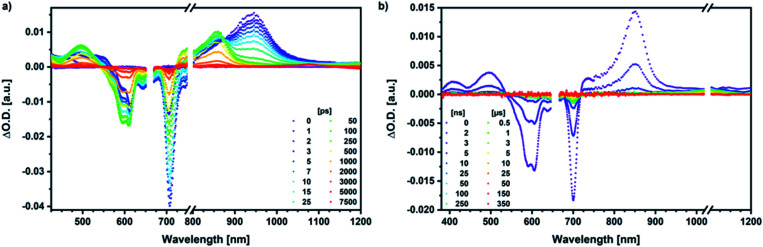
(a) Femtosecond differential absorption spectra of *syn*-SubPc **1** (1.5 × 10^−5^ M) in argon purged chlorobenzene at delay times between 0 and 7500 ps after 656 nm laser excitation (200 nJ) at room temperature. (b) Nanosecond differential absorption spectra of *syn*-SubPc **1** (1.5 × 10^−5^ M) in argon purged chlorobenzene at delay times between 0 and 350 μs after 656 nm laser excitation (450 nJ) at room temperature.

**Fig. 10:**
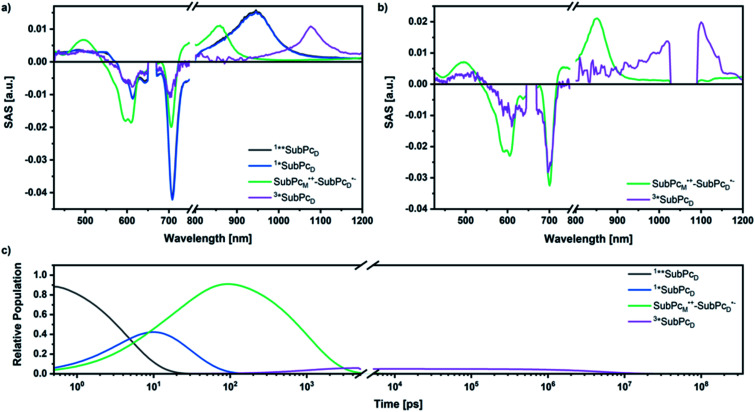
SAS obtained by global target analysis of *syn*-SubPc **1** in PhCl after 656 nm laser excitation of (a) femtosecond and (b) nanosecond transient absorption data. (c) Calculated population profiles (the break marks the crossing from the femtosecond to the nanosecond setup).

**Fig. 11:**
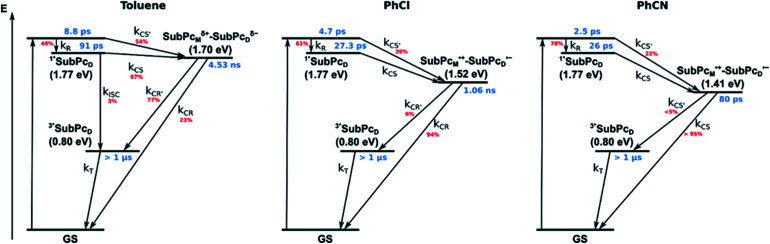
Energy diagrams of *syn*-SubPc **1** in toluene, PhCl, and PhCN illustrating the excited state deactivation after 656 nm laser excitation at room temperature. Lifetimes and scaling parameters were obtained by global target analysis of the transient absorption data.

Our investigation of *syn*-SubPc **1** started with excitation of the dimer-subunit at 656 nm in chlorobenzene (Fig. 9). Early on, we note ground-state bleaching solely from the dimer-subunit and a broad maximum in the nIR at 956 nm. As such, the singlet excited state of the dimer-subunit, that is, ^1^*SubPc_D_, is exclusively populated. Its fast decay fails to yield any triplet excited state. Instead, an intermediate state with maxima at 497 and 860 nm and additional ground state bleaching at 596 nm is noted. Important is the fact that reduction of dimer-SubPc **2** with Cp_2_Co in THF generated the same nIR signature (Fig. S10†). Considering this information, we postulate the formation of the SubPc_M_˙^+^–SubPc_D_˙^−^ charge separated state. Within a few nanoseconds the ground state and the triplet excited state with its nIR absorption maximum at around 1080 nm are formed. In more polar benzonitrile (Fig. S24†), the features of the charge separated state reach a maximum within 30 ps and fully decay after 400 ps without the appearance of any appreciable triplet excited state fingerprints at, for example, around 1080 nm. In less polar toluene (Fig. S25†), the charge separated state features maximize after several hundred ps and decay on the ns time-scale. Notably, residual ^1^*SubPc_D_ singlet excited state features are seen to decay on the same timescale. At this point, we postulate the formation of a charge transfer state, rather than a fully charge separated state. We further evaluated the transient absorption data by means of global target analysis. For chlorobenzene and benzonitrile, we postulate the following mechanism: first, higher lying singlet excited states ^1^**SubPc_D_ of the dimer-subunit are populated. They either relax to the lowest singlet excited state ^1^*SubPc_D_ or directly undergo charge separation to yield the SubPc_M_˙^+^–SubPc_D_˙^−^ charge separated state. Such a hole transfer dominates the deactivation of ^1^*SubPc_D_. Once the charge separated state is formed, it decays predominantly to the ground state and to a lesser extent to the triplet excited state. In toluene, a slightly different picture involves the formation of the SubPc_M_^*δ*+^–SubPc_D_^*δ*−^ charge transfer state rather than the fully charge separated state, which cannot be sufficiently stabilized in this unpolar medium. Please, note that an equilibrium between ^1^*SubPc_D_ and SubPc_M_^*δ*+^–SubPc_D_^*δ*−^ cannot be excluded. In such cases, the species associated spectra (SAS) resemble a superposition of the two states. Within the error of the Weller equation, the two excited states are practically isoenergetic. Further support of such an equilibrium comes from steady-state fluorescence spectroscopy. The shape and energetic position of the 702 nm fluorescence band bear strong resemblance to the fluorescence of dimer-SubPc **2** and may therefore stem from a dimer-centered local excited state, rather than the charge-transfer state. Considering the rather slow charge separation, we also accounted for intersystem crossing in our model. The resulting lifetimes and scaling parameters are visualized in the energy diagrams of Fig. 11. The corresponding species-associated spectra (chlorobenzene: Fig. 10; benzonitrile: Fig. S36;† toluene: Fig. S37†) are in sound agreement with the proposed model. The intrinsic lifetime of the relaxed SubPc_D_ singlet excited state decreases from 90 ps in toluene to 26 ps in PhCN. Hence, charge separation occurs faster in polar solvents and follows the trend expected for the normal regime of the Marcus parabola.^35^ On the other hand, charge recombination is slow in toluene, with an intrinsic lifetime of the charge transfer state of 4.53 ns. However, this particular lifetime may be virtually elongated by aforementioned excited state equilibrium. The fully charge separated states in PhCl and PhCN deactivate with intrinsic lifetimes of 1.06 ns and 80 ps, respectively, and, hence, the overall trend follows the behavior known for the Marcus inverted region.^35^

Next, we probed the energy transfer from SubPc_M_ to SubPc_D_ in *syn*-SubPc **1** by means of 550 nm laser excitation. In line with the spectral features of the monomer-subunit singlet excited state, the singlet excited state of the dimer-subunit and the charge separated state are discernible even at early timescales. Therefore, we hypothesize that the ^1^*SubPc_M_ deactivation must be faster than the time resolution of our experimental setup and, in turn, hampers any meaningful kinetic analysis.

**Fig. 12:**
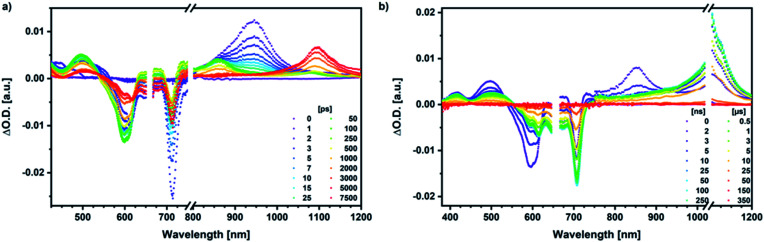
(a) Femtosecond differential absorption spectra of *syn*-SubPc **1** (1.5 × 10^−5^ M) and **C60** (3.0 × 10^−4^ M) in argon purged chlorobenzene at delay times between 0 and 7500 ps after 656 nm laser excitation (200 nJ) at room temperature. (b) Nanosecond differential absorption spectra of *syn*-SubPc **1** (1.5 × 10^−5^ M) and **C60** (3.0 × 10^−4^ M) in argon purged chlorobenzene with delay times between 0 and 350 μs after 656 nm laser excitation (450 nJ) at room temperature.

We rounded off our transient absorption pump probe studies with the excitation of *syn*-SubPc **1–C60** at 656 nm (Fig. 12). We used 20 equivalents of **C60** to ensure the complete complexation of *syn*-SubPc **1** (>90%). Immediately after photo-excitation, we note the features of the dimer-subunit singlet excited state – *vide supra*. Importantly, a faster decay is noted relative to the measurements in the absence of **C60** and the intensity of the radical anion band of the dimer-subunit is significantly decreased. In light of the above, we considered a parallel and a sequential scenario: in the context of the former, competition results in a suppression of the population. The latter, on the other hand, implies a faster deactivation into another state. Intriguingly, between 10 and 1000 ps the ground state bleaching mainly relates to that of the monomer-subunit, rather than that of the dimer-subunit. This implies that an intermediate state is formed, which does not involve the dimer-subunit of *syn*-SubPc **1**. Close inspection of the nIR region reveals maxima at 865 and 1080 nm. According to the literature, the latter is a fingerprint absorption of **C60**˙^−^.^36^ These findings imply the formation of the SubPc_M_˙^+^–**C60**˙^−^ charge separated state from the SubPc_M_˙^+^–SubPc_D_˙^−^ charge separated state. Interconversion of SubPc_M_˙^+^–**C60**˙^−^ yields either the ground state or the triplet excited state centered on the dimer-subunit, whose presence is validated by the 1093 nm maximum. Similar spectral changes are observed in benzonitrile (Fig. S26†) and in toluene, while any absorption features of SubPc_D_˙^−^ remain spectroscopically invisible (Fig. S27†).

**Fig. 13:**
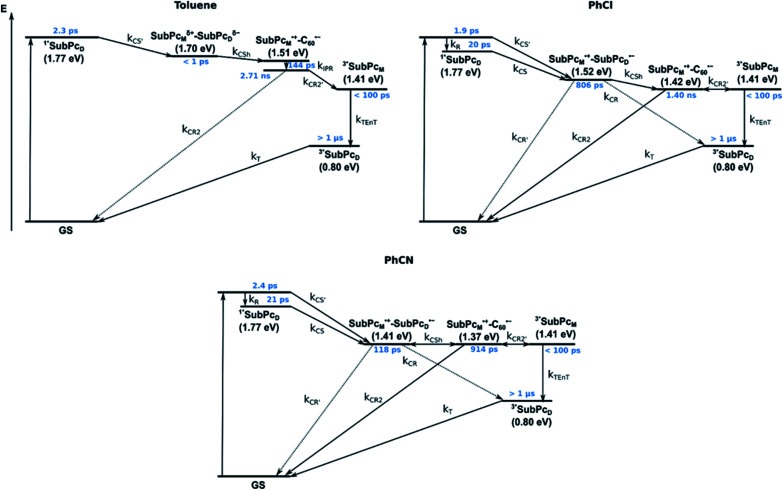
Energy diagrams of *syn*-SubPc **1–C60** in toluene, PhCl, and PhCN illustrating the excited state deactivation after 656 nm laser excitation at room temperature. Lifetimes were obtained by global analysis of the transient absorption data. Solid lines represent major pathways and dotted lines, minor ones.

From these observations, we infer the following excited state deactivation in chlorobenzene. The initially populated ^1^**SubPc_D_ either relaxes to ^1^*SubPc_D_ or undergoes charge separation to yield the SubPc_M_˙^+^–SubPc_D_˙^−^ charge separated state. ^1^*SubPc_D_ also deactivates *via* charge separation. Our experiments lack, however, any evidence for energy transfer, that is, formation of the **C60** singlet excited state (2.0 eV).^37^ The SubPc_M_˙^+^–SubPc_D_˙^−^ charge separated state undergoes a charge shift to yield the intermolecular SubPc_M_˙^+^–**C60**˙^−^ charge separated state. Notably, **C60** is a poorer electron acceptor than dimer-SubPc **2**. The proximity of **C60** and the monomer-subunit and, in turn, the gain in Coulomb energy due to the decrease in the electron donor–acceptor distance constitute the driving force for the charge shift process. From there, charge recombinations to afford either the ground state or the triplet excited state of the monomer-subunit, ^3^*SubPc_M_ (1.41 eV), are feasible, but not to the **C60** triplet excited state (1.6 eV).^37,38^ We did not, however, observe any ^3^*SubPc_M_ in our experiments. We propose that once the triplet excited state of the monomer-subunit is populated, rapid triplet–triplet energy transfer to the triplet excited state of the dimer-subunit takes place. Benzonitrile stands out: here, SubPc_M_˙^+^–SubPc_D_˙^−^, SubPc_M_˙^+^–**C60**˙^−^, and ^3^*SubPc_M_ are reasonably close in energy to allow an excited state equilibrium. An opposite reactivity is observed for non-polar toluene. Signatures of the charge separated state were not observed at all. We postulate that once the charge separated state is formed, it directly transforms into the more stable SubPc_M_˙^+^–**C60**˙^−^ charge separated state, which itself decays. In short, first recombination to ^3^*SubPc_M_ and successive triplet–triplet energy transfer are observed in all solvents. All processes are summarized in Fig. 13 together with the excited state lifetimes computed by fitting the raw data by means of global lifetime analysis.**The complexity of the processes and the presence of free hosts/guests render a profound global target analysis extraordinarily challenging. Anyway, global analysis of the raw data offers an adequate estimation of the excited state lifetimes. In polar PhCl and PhCN, the intrinsic lifetimes of the SubPc_D_ singlet excited state, relaxed and unrelaxed, remain almost unaffected by the presence of **C60**. Charge separation and charge recombination rates are, however, faster in PhCN than in PhCl.

**Fig. 14:**
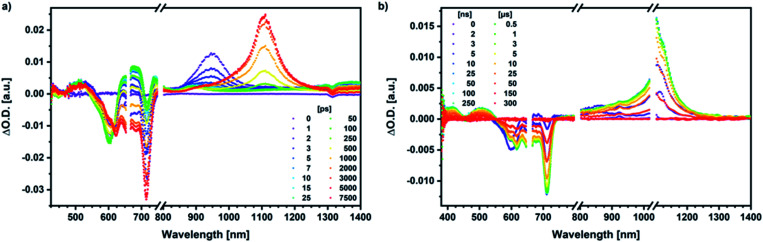
(a) Femtosecond differential absorption spectra of *syn*-SubPc **1** (1.5 × 10^−5^ M) and **C70** (3.0 × 10^−4^ M) in argon purged PhCl at delay times between 0 and 7500 ps after 656 nm laser excitation (300 nJ) at room temperature. (b) Nanosecond differential absorption spectra of *syn*-SubPc **1** (1.5 × 10^−5^ M) and **C70** (3.0 × 10^−4^ M) in argon purged PhCl at delay times between 0 and 350 μs after 656 nm laser excitation (300 nJ) at room temperature.

Similar processes are observed for *syn*-SubPc **1–C70** (Fig. 14). Following 656 nm laser excitation, we note the features of the singlet excited state of the dimer-subunit ^1^*SubPc_D_. Within less than 10 ps, these characteristics disappear and only an absorption maximum at 510 nm and a broad absorption between 630 and 710 nm are left behind. Furthermore, transient maxima at 890 and 1380 nm are discernible in the nIR. In line with the literature, the latter relates to the absorption of the **C70**˙^−^ radical anion.^39^ It exists for several hundreds of ps. At the same time, the ground-state bleaching intensity in the monomer-subunit region is at its maximum and that of the dimer-subunit at its minimum. This leads us to propose the formation of the SubPc_M_˙^+^–**C70**˙^−^ charge separated state. As the SubPc_M_˙^+^–**C70**˙^−^ charge separated state decays, features of ^3^*SubPc_D_, that is, ground-state bleaching at 715 nm and an intense nIR band at 1110 nm, increase. In comparison to the complex with **C60**, all excited state processes occur faster in the corresponding case with **C70**.

**Fig. 15:**
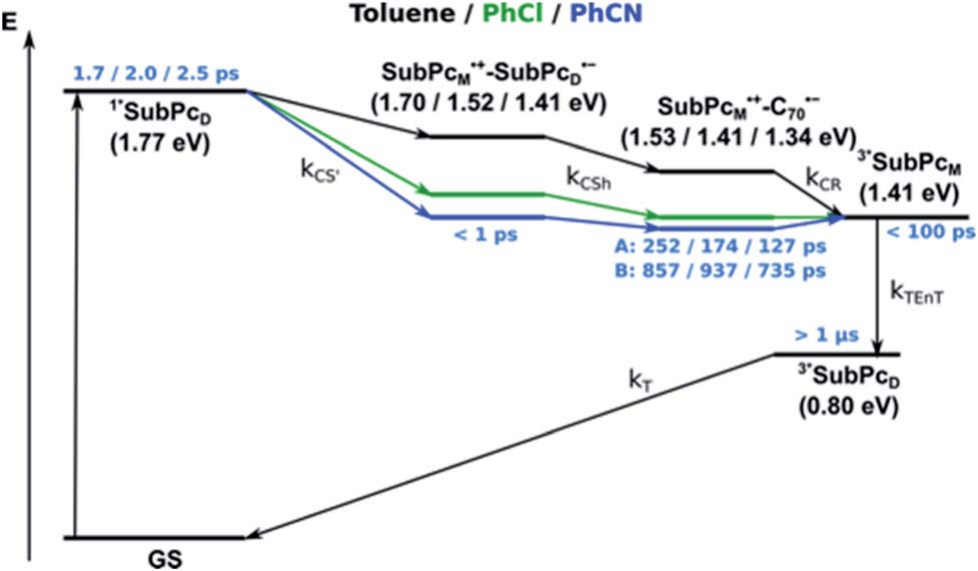
Energy diagrams of *syn*-SubPc **1–C70** in toluene, PhCl, and PhCN illustrating the excited state deactivation after 656 nm laser excitation at room temperature. Lifetimes were obtained by global analysis of the transient absorption data. Two species (A and B) with similar evolution associated spectra (EAS) were obtained for SubPc_M_˙^+^–**C70**˙^−^. (In toluene it is the SubPc_M_^*δ*+^–SubPc_D_^*δ*−^, rather than the fully separated radical ion pair.)

In summary (Fig. 15), the singlet excited state of the dimer-subunit is formed and undergoes charge separation to afford the SubPc_M_˙^+^–SubPc_D_˙^−^ charge separated state, rather than energy transfer to the higher lying **C70** singlet excited state (1.9 eV).^33^ The latter is subject to a fast charge shift to yield the SubPc_M_˙^+^–**C70**˙^−^ charge separated state, which hampers its spectroscopic observation.††††At this point, it has also to be considered that the SubPc_M_˙^+^–**C70**˙^−^ charge separated state may form directly from the ^1^*SubPc_D_ singlet excited state. In such a scenario, it has to be assumed that the excitation energy is partially distributed through the ethynyl spacer towards the monomer subunit. By means of global lifetime analysis,* we obtained two lifetimes for the SubPc_M_˙^+^–**C70**˙^−^ charge separated state, which are denoted as A and B in Fig. 15. A and B are likely to be two different orientations of **C70** or relate to a relaxation process of SubPc_M_˙^+^–**C70**˙^−^. Evidence for charge recombination to afford the triplet excited state of the monomer-subunit with subsequent triplet energy transfer to yield the final ^3^*SubPc_D_ comes from the spectroscopic data, even though the thermodynamics suggest an endergonic reaction in benzonitrile. However, these charge separated state energies come with a great caveat. Due to uphill thermodynamics, charge recombination to the **C70** triplet excited state (1.53 eV)^38,40^ is not expected.

## Conclusions

In summary, we report the synthesis of *syn*-SubPc **1**, in which an electron poor SubPc-dimer is connected to two electron-rich SubPc-monomers by means of a double Sonogashira reaction. The intrinsic pre-organized geometry of *syn*-SubPc **1** forms a pseudocavity of the size of fullerenes. Formation of 1 : 1 complexes with both **C60** and **C70** with association constants as high as 10^5^ M^−1^ was demonstrated using different spectroscopic techniques.

*syn*-SubPc **1** on its own is barely fluorescent in polar solvents due to fast charge separation to afford an intramolecular SubPc_M_˙^+^–SubPc_D_˙^−^ charge separated state. This state decays mainly to the ground state *via* a charge recombination of 1.06 ns in chlorobenzene and 80 ps in benzonitrile. In apolar toluene, however, transfer of charge density was observed rather than a separation of charges, which transforms into the triplet excited state of the SubPc-dimer.

In the presence of **C60** or **C70**, the intramolecular SubPc_M_˙^+^–SubPc_D_˙^−^ charge separated state undergoes a charge shift to yield the intermolecular SubPc_M_˙^+^–**C60**˙^−^/SubPc_M_˙^+^–**C70**˙^−^ charge separated state. Charge recombination yields, in the latter case, the SubPc-monomer triplet excited state, which subsequently undergoes triplet energy transfer to the SubPc-dimer triplet excited state.

## Conflicts of interest

There are no conflicts to declare.

## Supplementary Material

SC-011-D0SC00059K-s001
