# Supplementary material for: Photoactive preorganized subphthalocyanine-based molecular tweezers for selective complexation of fullerenes
Source: Chem Sci. 2020 Feb 28;11(13):3448–59. doi: 10.1039/d0sc00059k (PMC8515455; doi:10.1039/d0sc00059k)
Supplement: SC-011-D0SC00059K-s001 [file SC-011-D0SC00059K-s001.pdf]

Electronic Supplementary Material (ESI) for Chemical Science.
This journal is © The Royal Society of Chemistry 2020

Supporting Information

Photoactive Preorganized Subphthalocyanine-based Molecular Tweezers for Selective Complexation of Fullerenes

Germán Zango,[‡a] Marcel Krug,[‡b] Swathi Krishna,[b] Víctor Mariñas,[a] Timothy Clark,[b] M. Victoria Martinez-Diaz,[a,d] Dirk M. Guldi[b] and Tomás Torres[a,c,d]

[a]Department of Organic Chemistry, Universidad Autónoma de Madrid, c/Francisco Tomás y Valiente 7, Cantoblanco, 28049 Madrid, Spain.
[b]Friedrich-Alexander-Universität Erlangen-Nürnberg, Department of Chemistry and Pharmacy & Interdisciplinary Center for Molecular Materials (ICMM), Egerlandstrasse 3, Erlangen 91058, Germany.
[c]IMDEA-Nanociencia, c/Faraday 9, Campus de Cantoblanco, 28049 Madrid, Spain.
[d]Institute for Advanced Research in Chemical Sciences (IAdChem), Universidad Autónoma de Madrid, 28049 Madrid, Spain.

Table of Contents

1. Materials and General Methods 1
2. Synthetic procedures and molecular characterization of compounds 1, 3 and 4 3
3. Steady-State Absorption and Emission Spectroscopy 10
4. Titration Experiments 12
5. Femtosecond Transient Absorption Spectroscopy 15
6. Global Target Analysis 20
7. Calculated Structures 24
8. References 51

1. Materials and General Methods

All chemical reagents were purchased from Merck, TCI Europe N.V., Alfa Aesar Acros Organics or Fluka Chemie and used without further purification. Solvents were purchased from Carlo Erba Reagents, Scharlab, Carl Roth and Sigma Aldrich. "Synthetic grade" solvents were used for chemical reactions and column chromatography purifications and "anhydric grade" for reactions under dry conditions. Additionally, some solvents were further dried by distillation with Na/benzophenone (THF), or with previously activated molecular sieves (3 or 4 Å), or with a solvent purifying system by Innovative Technology Inc. HPLC grade solvents were employed for the spectroscopic characterization and anhydrous MeTHF was used for cryogenic glass matrices. MD-4-PS. 4,5-diiodophthalonitrile,[1] 4,5-dichlorophthalonitrile[2] and 4,5-dioctylthiophthalonitrile[3] were prepared using a described procedure. The synthesis and characterization of SubPc dimers *syn*-**2** and *anti*-**2** has been described in the literature.[4]

Axial substitution reaction of a 1:1 syn/anti mixture of dimer-SubPc 2 with 5 equivalents of ethynyltrimethylsilane and 4 equivalents of AlCl3 yielded 3 as a 1:1 syn/anti mixture in 60% yield. The small size and low polarity of the ethynyl axial substituents precluded the separation of both topoisomers by chromatographic work up on silica gel. Indeed, identical 1H-NMR and UV-vis features were recorded when each topoisomer was individually synthesized and characterized by starting from isomerically pure dimeric syn-SubPc dimer 2 or anti-SubPc dimer 2. Finally, Pd-catalyzed crossed-coupling reaction of 3 with monomer-SubPc 4 led to the formation of syn-SubPc 1 and anti-SubPc 1, in 15% and 7% yields, respectively, after purification by column chromatography on silica gel

The monitoring of the reactions was carried out by TLC, employing aluminum sheets coated with silica gel type 60 F_{254} (0.2 mm thick, E. Merck). The analysis of the TLCs was carried out with an UV lamp of 254 and 365 nm. Purification and separation of the synthesized products was performed by column chromatography, using silica gel (230-400 mesh, 0.040-0.063 mm, Merck). Size exclusion chromatography was performed using Bio-Beads S-X1 (200-400 mesh, Bio-Rad).

^{1}H NMR and ^{13}C NMR spectra were recorded on a Bruker AC-300 (300 MHz) or a Bruker DRX-500 (500 MHz) either in the Organic Chemistry Department or in Servicio Interdepartamental de Investigación (SIdI, Interdepartmental Investigation Service) at the Autónoma University of Madrid. The temperature was actively controlled at 298 K. Chemical shifts are measured in ppm relative to the correspondent deuterated solvent.

MS and HR-MS were recorded in Servicio Interdepartamental de Investigación (SIdI, Interdepartmental Investigation Service) at the Autónoma University of Madrid, using a VG-AutoSpec spectrometer for EI and FAB-MS and a Bruker Reflex III spectrometer, with a nitrogen laser operating at 337 nm, for MALDI-TOF. The different matrixes employed are indicated for each spectrum.

Electrochemical measurements were performed on an Autolab PGStat 30 equipment using a three electrode configuration system. The measurements were carried out using freshly distilled THF solutions containing 0.1 M tetrabutylammonium hexafluorophosphate ($TBAPF_6$) and a

concentration of approximately 10^{-4} M of the corresponding compound. A glassy carbon electrode (3 mm diameter) was used as the working electrode, and a platinum wire and an $Ag/AgNO_3$ (in CH_3CN) electrode were employed as the counter and the reference electrodes, respectively. Ferrocene (Fc) was used as an external reference and all the potentials were given relative to the Fc/Fc^+ couple. Scan rate was 100 mV s^{-1} unless otherwise specified.

Steady state absorption spectra were obtained using a PerkinElmer Lambda 2 dual beam absorption spectrometer with a scan rate of 480 nm/min or a Varian Cary 5000. Steady state emission spectra were recorded using a Horiba Jobin Yvon FluoroMax-3 emission spectrometer with 2.5 nm spectral bandwidth and 0.25 s integration time. Phosphorescence spectra were obtained with a Fluorolog-3 setup and a Symphony InGaAs detector. Second order diffraction signals were suppressed by a 780 nm longpass-filter after the sample. Cooling the samples to 80 K was achieved with liquid nitrogen cooled Oxford Instruments Optistat DN cryostat. Time-correlated single-photon counting was performed on a Horiba Jobin Yvon FluoroLog-3 setup. The excitation light source was a SuperK Extreme EXB-6 laser from NKT-Photonics and the detector a Hamamatsu MCP photomultiplier (R33809U-50). Fit and lifetime analysis was performed with the TCSPC software provided with the instrument. Samples were contained in 10 x 10 mm quartz cuvettes and degassed with argon for 15 min in the case of TCSPC measurements. Chemical reduction experiments were executed in a Jacomex: GB Campus (GP) Glovebox and measurements were performed in wax-sealed cuvettes.

Femtosecond and nanosecond transient absorption studies were performed with the HELIOS (0 to 8000 ps) or EOS system (1 ns to 350 µs) from Ultrafast Systems. The laser source was a Clark MXR CPA2110 and CPA2101 Ti:Sapphire amplifier with a pulsed 1 kHz output centered at 775 nm with 150 fs pulse width. The excitation pulses of 550 and 656 nm were generated with a NOPA and small spectral width was ensured by a band-pass filter (FWHM 10 nm). The white light for the femtosecond experiments was generated by focusing the 775 nm fundamental output into a 2 mm (vis: 420 – 760 nm) or 1 cm (NIR: 800 – 1500 nm) Sapphire disk. In the case of nanosecond experiments, the white light (370 – 1600 nm) came from a supercontinuum laser source with 2 kHz repetition rate and a pulse width of approximately 1 ns. Samples were contained in 2 x 10 mm optical glass cuvettes and purged with argon for 15 min. Global Target Analysis of transient absorption data was performed with the GloTarAn software.[5]

Gaussian 16[6] was used to perform theoretical treatment of the compounds. Geometry optimizations were performed on the B3LYP[7]/def2-SVP[8,9] level of theory with GD3-BJ[10] to account for dispersion interactions. To improve the computational efficiency, octyl- and *t*-butyl groups were treated as methyl groups.

2. Synthetic procedures and molecular characterization of compounds 1, 3 and 4

2.1. Synthetic procedures

Subphthalocyanine dimer **3**, mixture of *syn* and *anti* topoisomers

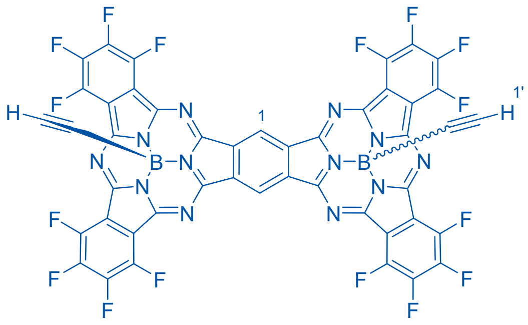

A 10 mL Schlenk flask was charged with dimer **2** (1:1 mixture of *syn* and *anti*, 160 mg, 0.150 mmol), ethynyltrimethylsilane (104 µL, 0.750 mmol) and *o*-dichlorobenzene (8 mL). The resulting solution was deoxygenated via three Freeze-Pump-Thaw cycles, $AlCl_3$ (80 mg, 0.600 mmol) was added and the suspension stirred at 25 °C for 30 min under argon atmosphere. Pyridine (0.1 mL) was added and solvent was removed by rotary evaporation. The dark blue reaction slurry was dissolved in CH_2Cl_2 and passed through a short silica plug. The solvent was removed by vacuum distillation and the resulting solid was subjected to column chromatography on silica gel using toluene/heptane 5:1 as an eluent to afford 94 mg (0.090 mmol) of compound **3** as a blue solid. Overall yield: 60% (*syn*/*anti* 1:1 mixture).

^{1}H-NMR (300 MHz, $CDCl_3$): δ (ppm) = 10.39 (s, 2H; H_1), 1.10 (s, 4H; $H_{1'}$).

MS (MALDI-TOF, DCTB): *m/z* = 1050.1 $[M]^+$.

HRLSI-MS: *m/z* Calcd for $[C_{46}H_4B_2F_{16}N_{12}]$: 1050.0622; Found: 1050.0608.

UV-vis ($CHCl_3$): λ_{max} (nm) (log ε ($dm^3\ mol^{-1}\ cm^{-1}$)) = 692 (5.0), 662 (4.5), 636 (4.5), 605 (4.6), 592 (sh), 441 (4.0), 320 (4.6), 278 (4.5).

Subphthalocyanine **4**

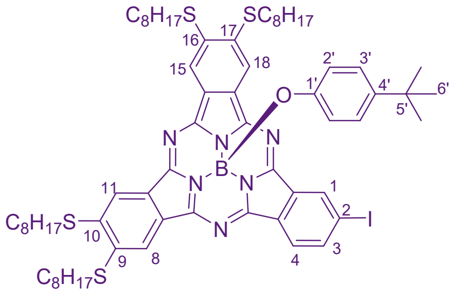

To a 25 ml round-bottom two-neck flask equipped with a reflux condenser and magnetic stirrer, a 1.0 M solution of BCl_3 in *p*-xylene (3 mL) was added to a mixture of 4-iodophthalonitrile (255 mg, 1.00 mmol) and 4,5-dioctylthiophthalonitrile (833 mg, 2.00 mmol), under argon atmosphere. The mixture was stirred and heated to reflux for 45 min. The crude was cooled down to room temperature and flushed with argon. After evaporation of *p*-xylene, 4-*tert*-butylphenol (750 mg, 5.00 mmol) was added. The mixture was dissolved in *ca.* 3 mL of dry toluene and refluxed for 4 hours. After cooling down to room temperature, the excess of phenol was removed by washing the crude with a 3:1 MeOH/water solution. The resulting dark solid was subjected to column chromatography on silica gel using heptane/EtOAc from 15:1 to 5:1 as an eluent to afford 271 mg (0.220 mmol) of compound **4** as a greenish oil. Yield: 22%.

^{1}H-NMR (300 MHz, $CDCl_3$): δ (ppm) = 9.17 (s, 1H; H_1), 8.61-8.56 (m, 4H; H_8, H_{11}, H_{15}, H_{18}), 8.52 (d, J_o = 8.3 Hz, 1H; H_4), 8.14 (dd, J_o = 8.3 Hz, J_m = 1.5 Hz, 1H; H_3), 6.76 (d, J_o = 8.7 Hz, 2H; $H_{3'}$), 5.31 (d, J_o = 8.7 Hz, 2H; $H_{2'}$), 3.33-3.15 (m, 8H; $S\underline{CH_2}$), 1.93-1.79 (m, 8H; $SCH_2\underline{CH_2}$), 1.67-1.51 (m, 8H; $S(CH_2)_2\underline{CH_2}$), 1.46-1.24 (m, 32H; $S(CH_2)_3(\underline{CH_2})_4$), 1.08 (s, 9H; $H_{6'}$), 0.93-0.85 (m, 12H; $\underline{CH_3}$).

MS (MALDI-TOF, DCTB): *m/z* = 1246.3 $[M]^+$, 1097.3 $[M - \text{axial group}]^+$.

UV-vis ($CHCl_3$): λ_{max} (nm) (log ε ($dm^3\ mol^{-1}\ cm^{-1}$)) = 589 (4.9), 544 (sh), 414 (4.5), 355 (4.6), 302 (4.8), 284 (4.8).

SubPc-SubPc dimer *syn*-SubPc **1**

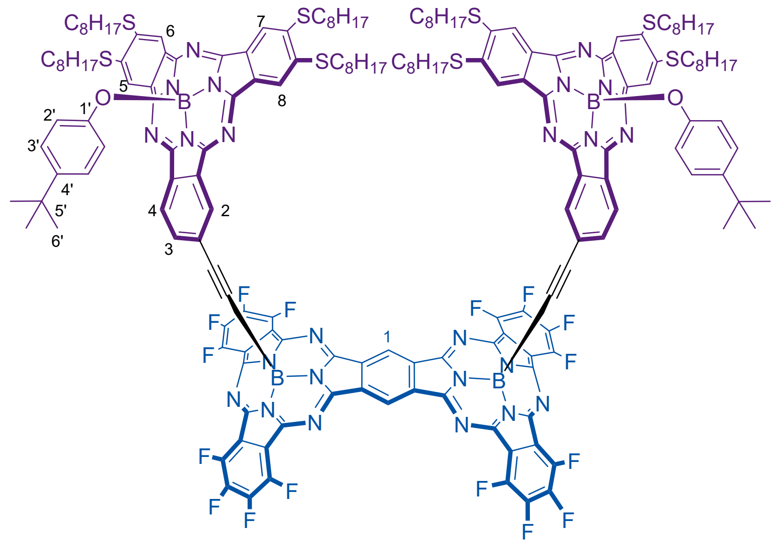

A 10 mL Schlenk flask was charged with SubPc dimer **3** (1:1 mixture of *syn* and *anti*, 50 mg, 0.048 mmol), CuI (1.9 mg, 0.010 mmol), $PdCl_2(PPh_3)_2$ (7.0 mg, 0.010 mmol), dry THF (4 mL) and triethylamine (1 mL) and the suspension was deoxygenated via three Freeze-Pump-Thaw cycles. SubPc **4** (125 mg, 0.100 mmol) was added and the suspension stirred at 25 °C for 1 h under argon atmosphere. The reaction slurry was dissolved in CH_2Cl_2 and passed through a short silica plug. The solvent was removed by vacuum distillation and the resulting solid was subjected to column chromatography on silica gel using $CHCl_3$/heptane 2:1 as an eluent and purification to obtain a pure fraction of dimer *syn*-**1** and a second fraction containing a mixture of dimers *syn*-SubPc **1**, *anti*-SubPc **1** and side products. By collecting the pure fractions of dimer *syn*-SubPc **1** obtained from several successive chromatography columns, a total amount of 24 mg (0.0073 mmol) of dimer *syn*-SubPc **1** was obtained as a dark purple solid. Yield: 15%.

^{1}H-NMR (300 MHz, $CDCl_3$): δ (ppm) = 10.50-10.45 (m, 2H; H_1), 8.49 (s, 4H; H_6, H_7), 8.44 (s, 2H; H_5), 8.40 (s, 2H; H_8), 8.37 (d, J_o = 8.8 Hz, 2H; H_4), 8.22 (d, J_m = 1.2 Hz, 2H; H_2), 7.17 (dd, J_o = 8.8 Hz, J_m = 1.2 Hz, 2H; H_3), 6.64 (d, J_o = 8.7 Hz, 4H; $H_{3'}$), 5.15 (d, J_o = 8.7 Hz, 4H; $H_{2'}$), 3.28-3.03 (m, 16H; $S\underline{CH_2}$), 1.89-1.72 (m, 16H; $SCH_2\underline{CH_2}$), 1.60-1.46 (m, 16H; $S(CH_2)_2\underline{CH_2}$), 1.40-1.20 (m, 64H; $S(CH_2)_3(\underline{CH_2})_4$), 0.99 (s, 18H; $H_{6'}$), 0.92-0.79 (m, 24H; $\underline{CH_3}$).

MS (MALDI-TOF, DCTB): *m/z* = 3288.3 $[M]^+$, 3139.3 $[M - \text{SubPc axial group}]^+$.

HRLSI-MS: *m/z* Calcd for $[C_{178}H_{178}B_4F_{16}N_{24}O_2S_8]$: 3288.2513; Found: 3288.2500.

UV-vis ($CHCl_3$): λ_{max} (nm) (log ε ($dm^3\ mol^{-1}\ cm^{-1}$)) = 696 (5.1), 664 (4.6), 635 (4.6), 594 (5.2), 543 (sh), 434 (4.5), 355 (sh), 308 (5.1), 281 (5.1).

SubPc-SubPc dimer *anti*-SubPc **1**

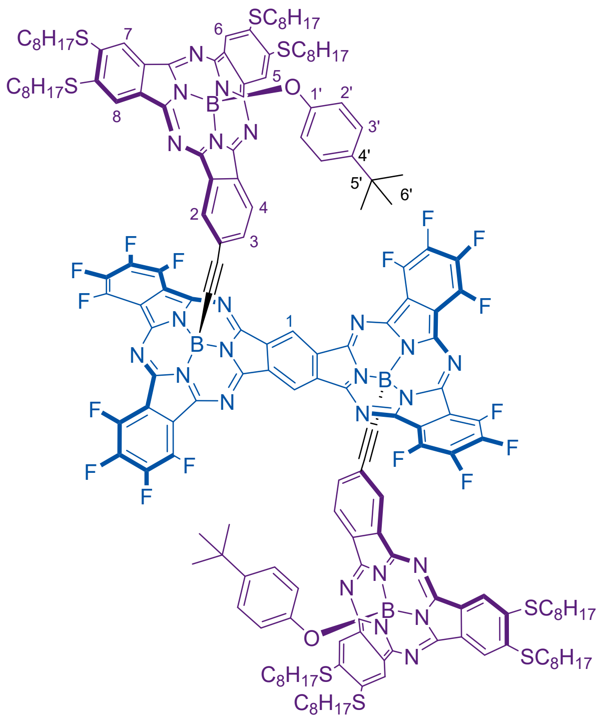

A mixture of dimers *syn*-SubPc **1**, *anti*-SubPc **1** and side products was subjected to size exclusion chromatography using THF as eluent to render 11 mg (0.0033 mmol) of dimer *anti*-SubPc **1** as a dark purple solid. Yield: 7%.

^{1}H-NMR (300 MHz, $CDCl_3$): δ (ppm) = 10.27-10.20 (m, 2H; H_1), 8.53-8.30 (m, 10H; H_4, H_5, H_6, H_7, H_8), 8.16 (s, 2H; H_2), 7.08 (d, J_o = 8.8 Hz, 2H; H_3), 6.62 (d, J_o = 8.5 Hz, 4H; $H_{3'}$), 5.14 (d, J_o = 8.5 Hz, 4H; $H_{2'}$), 3.29-3.05 (m, 16H; S$\underline{CH_2}$), 1.89-1.72 (m, 16H; $SCH_2\underline{CH_2}$), 1.60-1.45 (m, 16H; $S(CH_2)_2\underline{CH_2}$), 1.41-1.20 (m, 64H; $S(CH_2)_3(\underline{CH_2})_4$), 0.98 (s, 18H; $H_{6'}$), 0.92-0.79 (m, 24H; $\underline{CH_3}$).

MS (MALDI-TOF, DCTB): *m/z* = 3288.3 [M]$^+$, 3139.3 [M - SubPc axial group]$^+$, 2143.6 [M - SubPc unit]$^+$, 1144.6 [SubPc-ethynyl]$^+$, 1010.6 [SubPc-ethynyl- axial group + OH]$^+$.

HRLSI-MS: *m/z* Calcd for [$C_{178}H_{178}B_4F_{16}N_{24}NaO_2S_8$]: 3311.2410; Found: 3311.2296.

UV-vis ($CHCl_3$): λ_{max} (nm) (log ε ($dm^3\ mol^{-1}\ cm^{-1}$)) = 696 (5.1), 666 (4.6), 637 (4.6), 594 (5.2), 544 (sh), 434 (4.5), 353 (sh), 311 (5.1), 279 (5.1).

2.2. NMR spectra

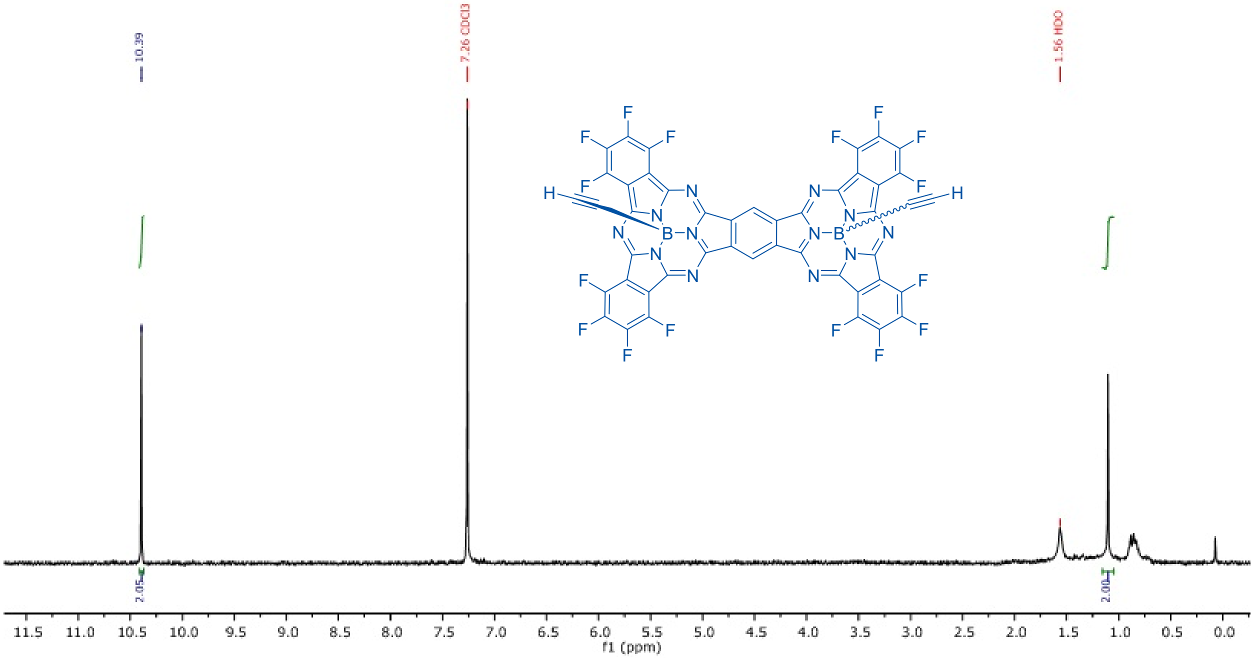

Figure S1: ^{1}H-NMR spectrum of SubPc **3** in $CDCl_3$ (300 MHz, 298 K).

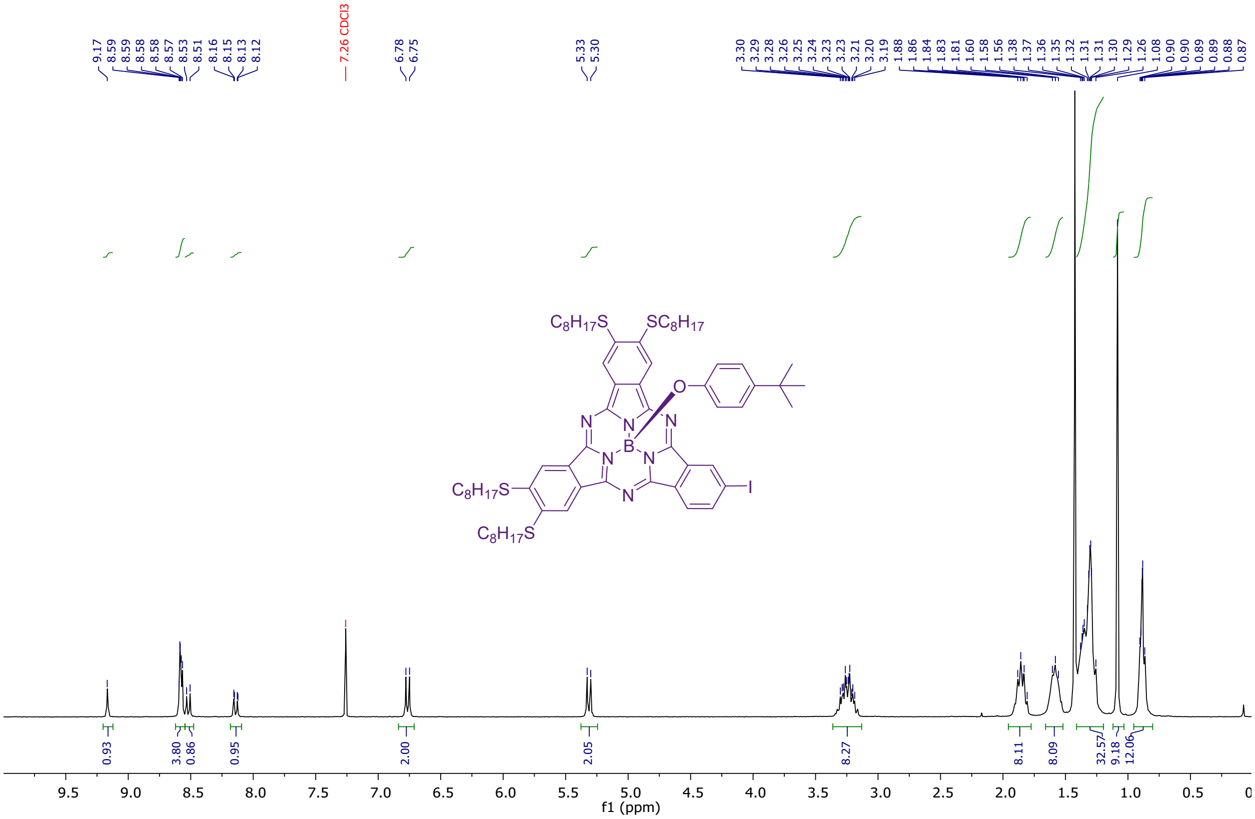

Figure S2: ^{1}H-NMR spectrum of SubPc **4** in $CDCl_3$ (300 MHz, 298 K).

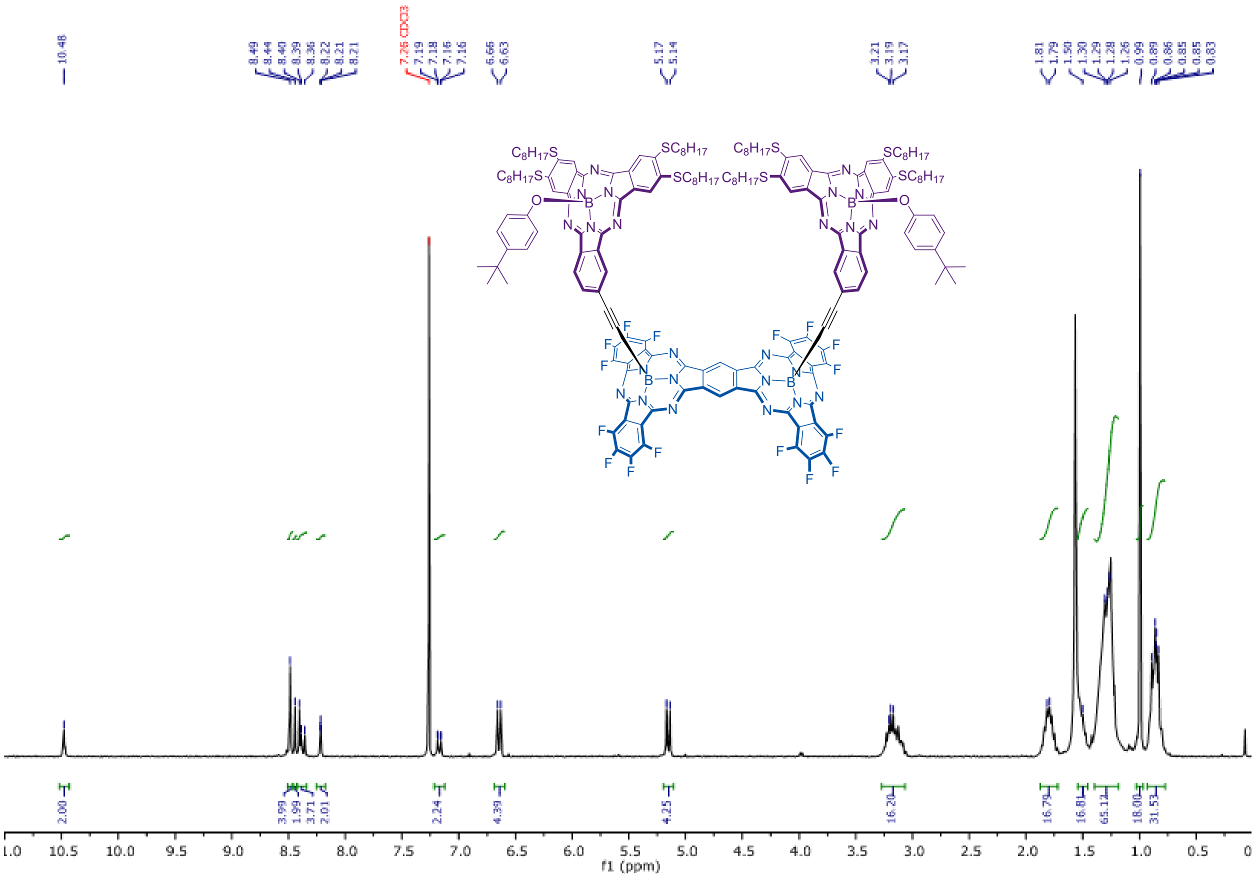

Figure S3: ^{1}H-NMR spectrum of *syn*-SubPc **1** in $CDCl_3$ (300 MHz, 298 K).

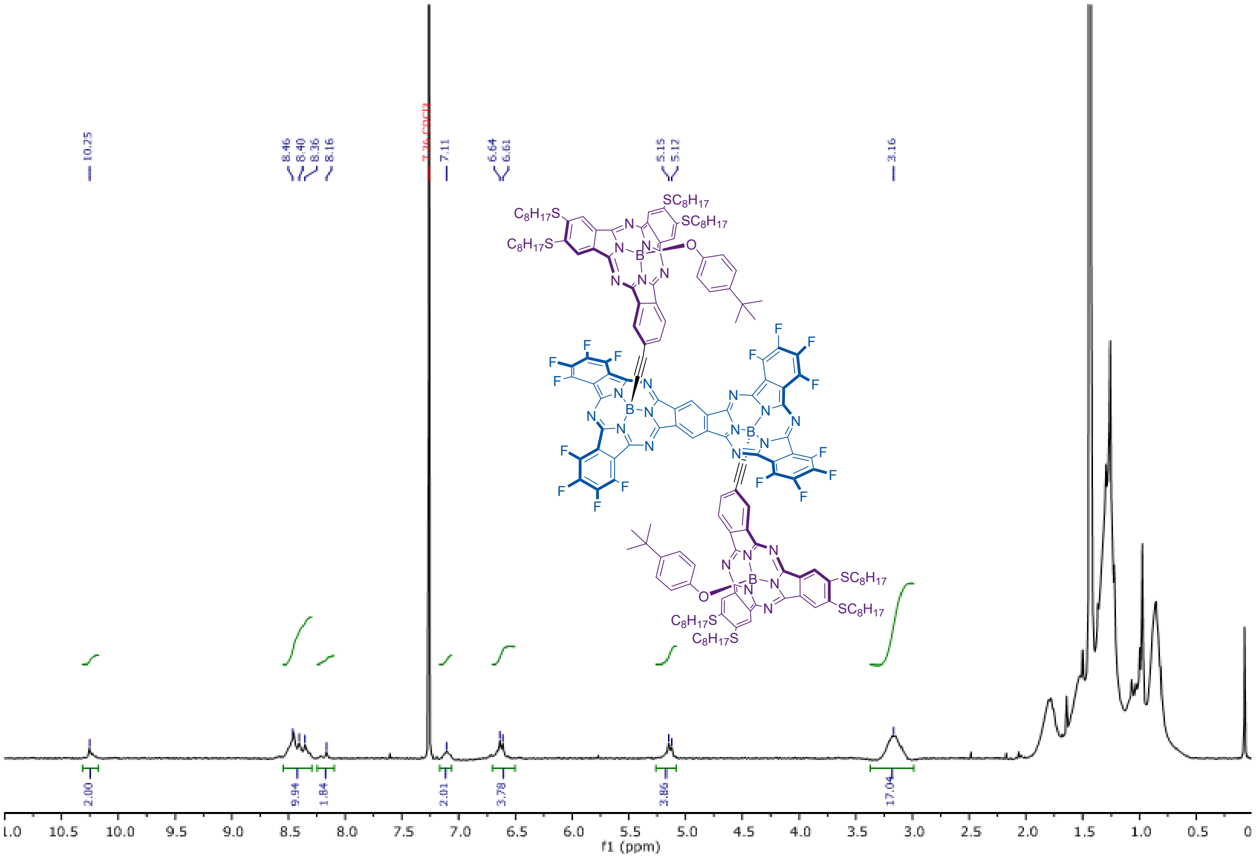

Figure S4: ^{1}H-NMR spectrum of *anti*-SubPc **1** in $CDCl_3$ (300 MHz, 298 K).

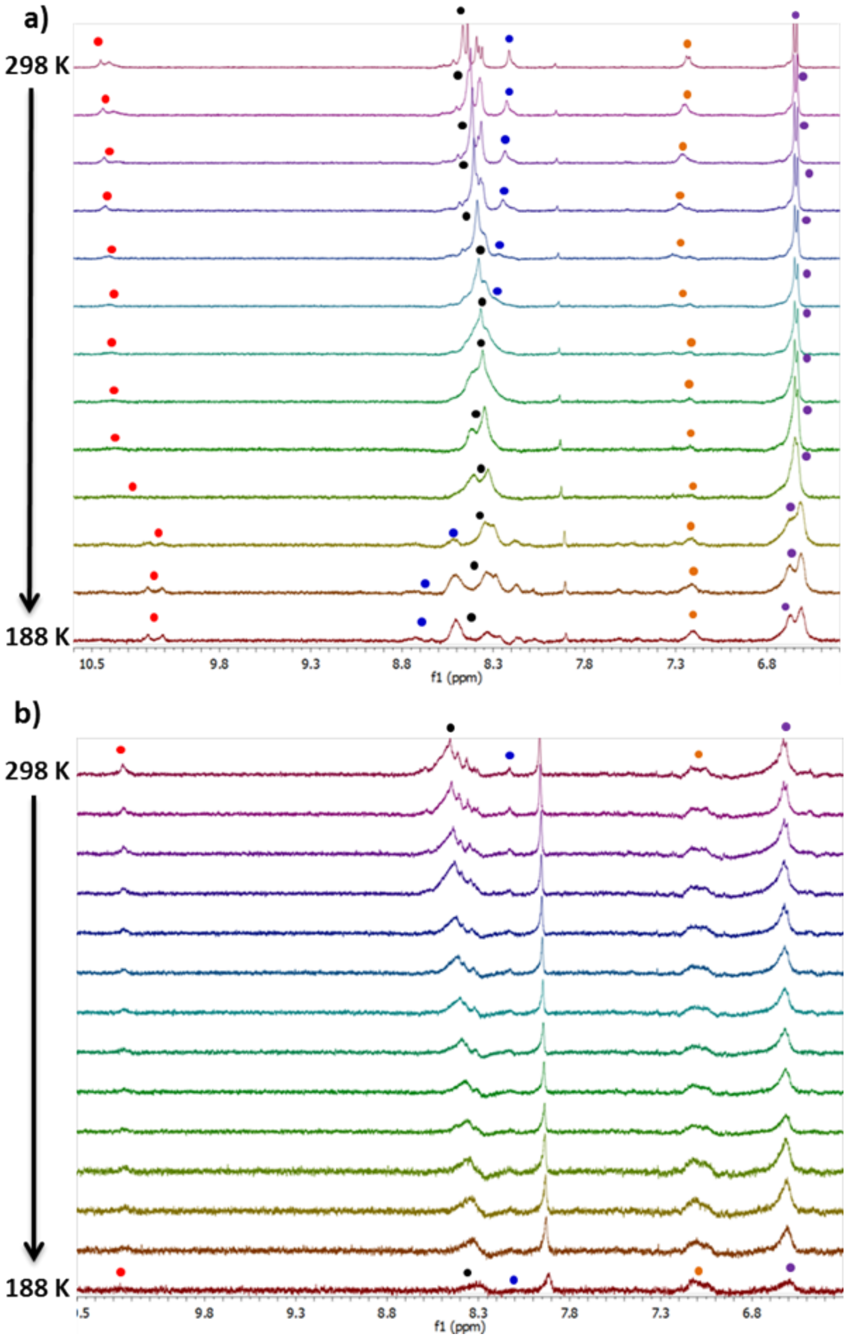

Figure S5: Partial ^{1}H-NMR spectra at variable temperature in CD_2Cl_2 (300 MHz, 298-188 K) of compounds a) *syn*-SubPc **1** and b) *anti*-SubPc **1**.

3. Steady-State Absorption and Emission Spectroscopy

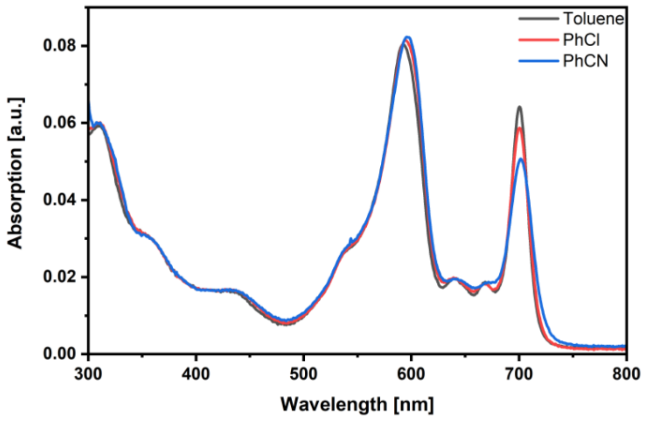

Figure S6: Steady-state absorption spectra of *syn*-SubPc **1** in toluene, PhCl and PhCN.

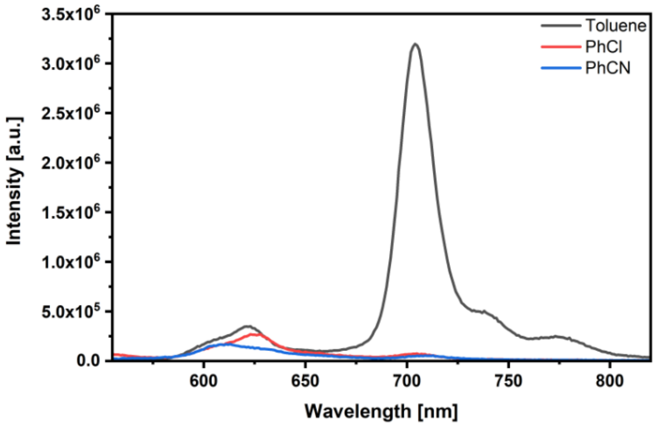

Figure S7: Steady-State emission spectra of *syn*-SubPc **1** in toluene, PhCl and PhCN.

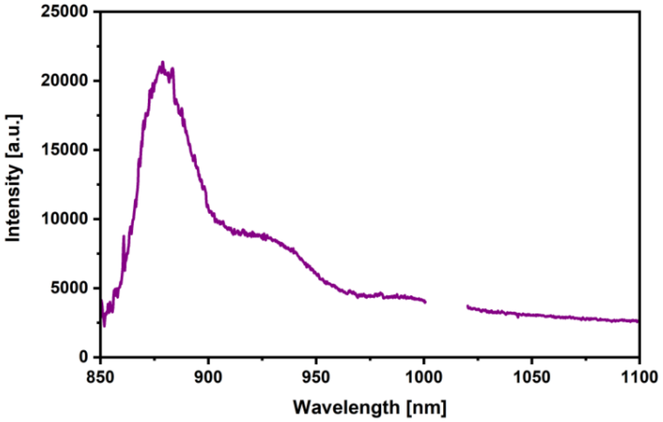

Figure S8: Phosphorescence spectrum of SubPc 4 in MeTHF glass matrix at 100 K after 550 nm excitation.

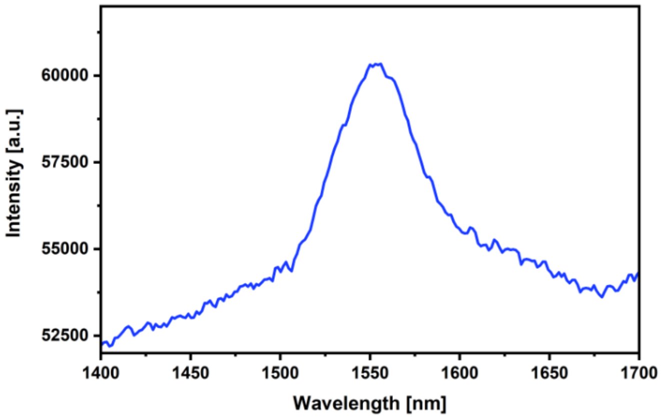

Figure S9: Phosphorescence spectrum of SubPc **2** in MeTHF glass matrix at 100 K after 640 nm excitation.

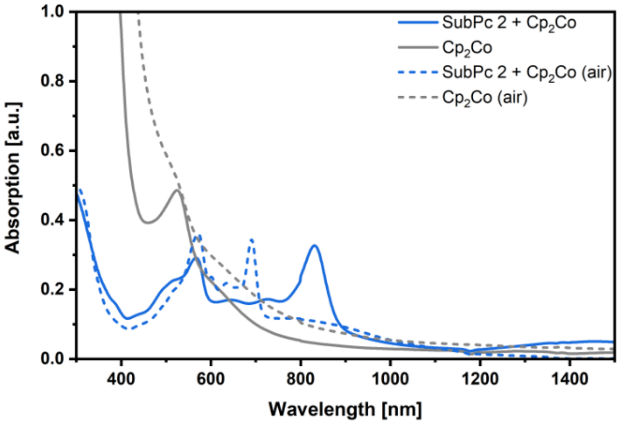

Figure S10: Absorption spectra of the SubPc **2** radical anion obtained after chemical reduction in dry THF (blue solid). The re-oxidation with air shows the reversibility of this process (blue dashed). Neither pristine Cp_2Co (grey solid), nor its oxidized form (grey dashed) show a feature in the nIR at 830 nm.

4. Titration Experiments

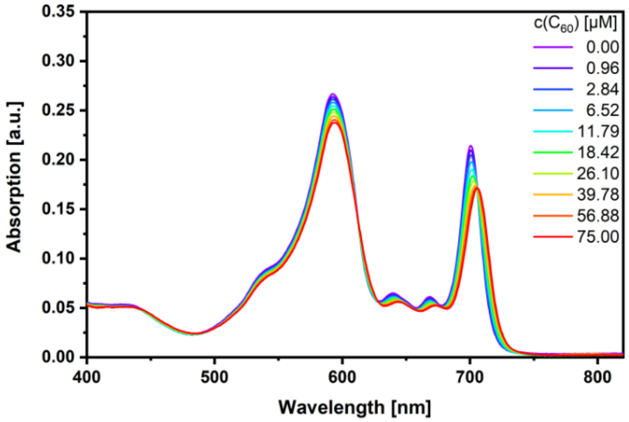

Figure S11: Steady-state absorption spectra of *syn*-SubPc **1** (1.5×10^{-6} M) in toluene with increasing amount of C_{60} (0 – 75 μM).

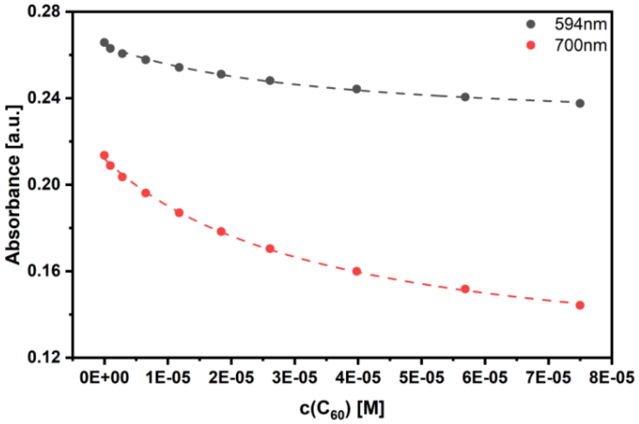

Figure S12: Binding isotherms obtained during the titration of *syn*-SubPc **1** with C_{60} by means of steady-state absorption spectroscopy.

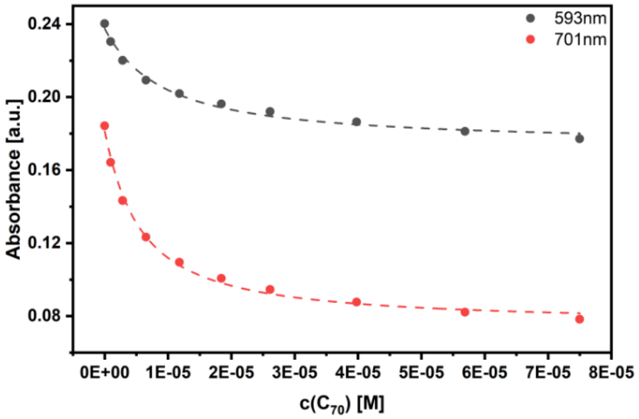

Figure S13: Binding isotherms obtained during the titration of *syn*-SubPc **1** with C_{70} by means of steady-state absorption spectroscopy.

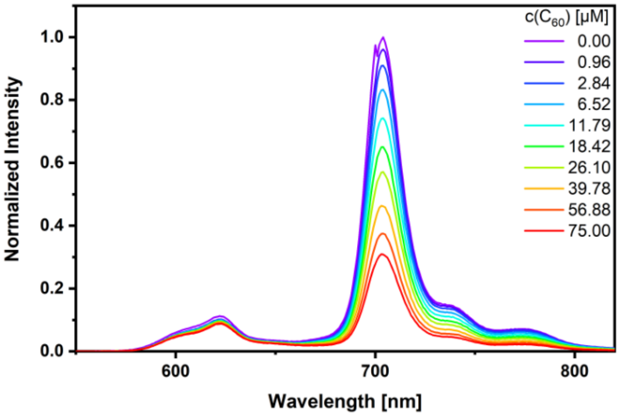

Figure S14: Steady-state fluorescence spectra of *syn*-SubPc **1** (1.5 x 10^{-6} M) after 440 nm excitation in toluene with increasing amount of C_{60} (0 – 75 µM).

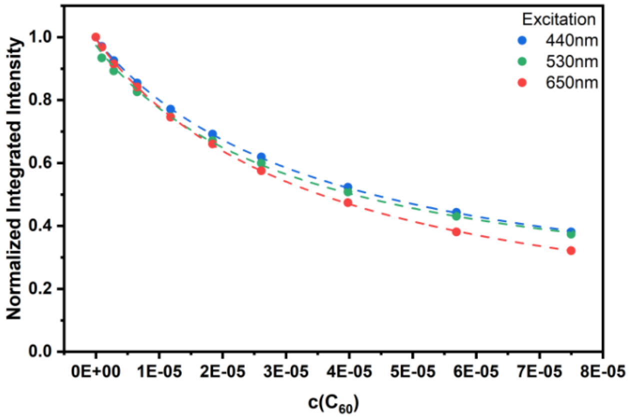

Figure S15: Binding isotherms obtained during the titration of *syn*-SubPc **1** with C_{60} by means of steady-state fluorescence spectroscopy.

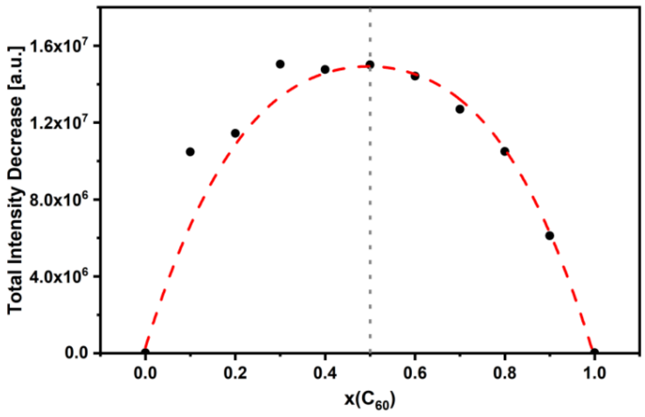

Figure S16: Plot of the SubPc dimer fluorescence decrease as a function of x(C_{60}). The data was fitted by a pseudo-Voigt function and the grey line highlights the maximum at x(C_{60}) = 0.5.

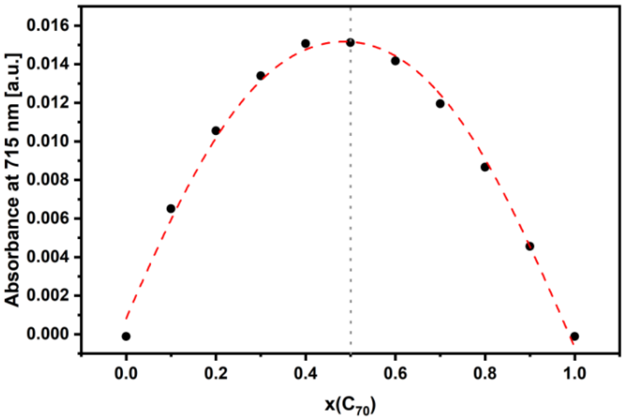

Figure S17: Plot of the absorbance at 715 nm as a function of x(C_{70}). The data was fitted by a pseudo-Voigt function and the grey line highlights the maximum at x(C_{70}) = 0.5.

5. Femtosecond Transient Absorption Spectroscopy

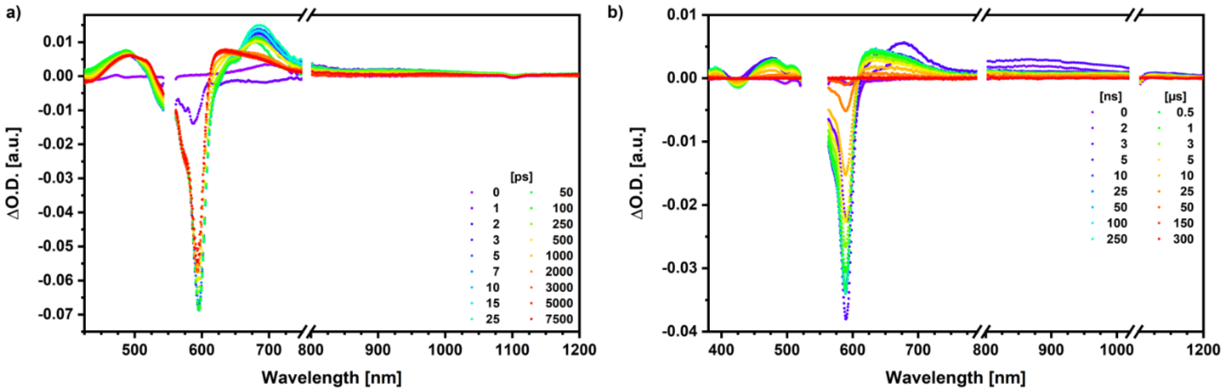

Figure S18: Femtosecond differential absorption spectra of SubPc **4** (1.5 x 10^{-5} M) in argon purged toluene at delay times between 0 and 7500 ps after 550 nm laser excitation (500 nJ) at room temperature. b) Nanosecond differential absorption spectra of SubPc **4** (1.5 x 10^{-5} M) in argon purged toluene at delay times between 0 and 300 µs after 550 nm laser excitation (500 nJ) at room temperature.

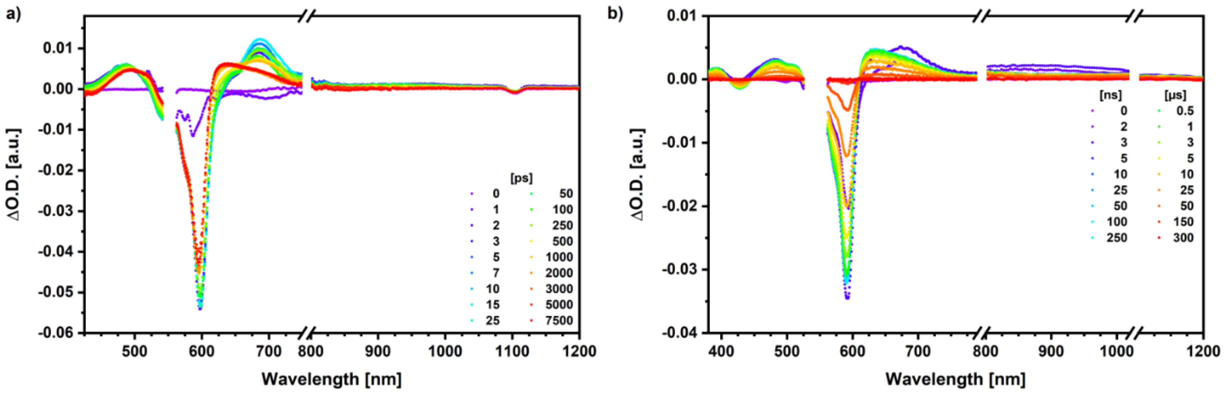

Figure S19: Femtosecond differential absorption spectra of SubPc **4** (1.5 x 10^{-5} M) in argon purged PhCl at delay times between 0 and 7500 ps after 550 nm laser excitation (500 nJ) at room temperature. b) Nanosecond differential absorption spectra of SubPc **4** (1.5 x 10^{-5} M) in argon purged PhCl at delay times between 0 and 300 µs after 550 nm laser excitation (500 nJ) at room temperature.

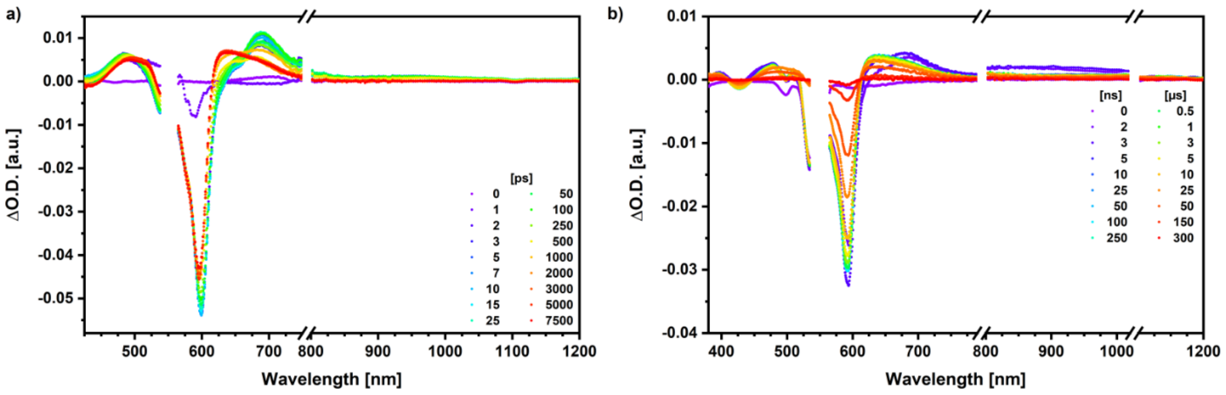

Figure S20: Femtosecond differential absorption spectra of SubPc **4** (1.5 x 10^{-5} M) in argon purged PhCN at delay times between 0 and 7500 ps after 550 nm laser excitation (500 nJ) at room temperature. b) Nanosecond differential absorption spectra of SubPc

4 (1.5 x 10^{-5} M) in argon purged PhCN at delay times between 0 and 300 µs after 550 nm laser excitation (500 nJ) at room temperature.

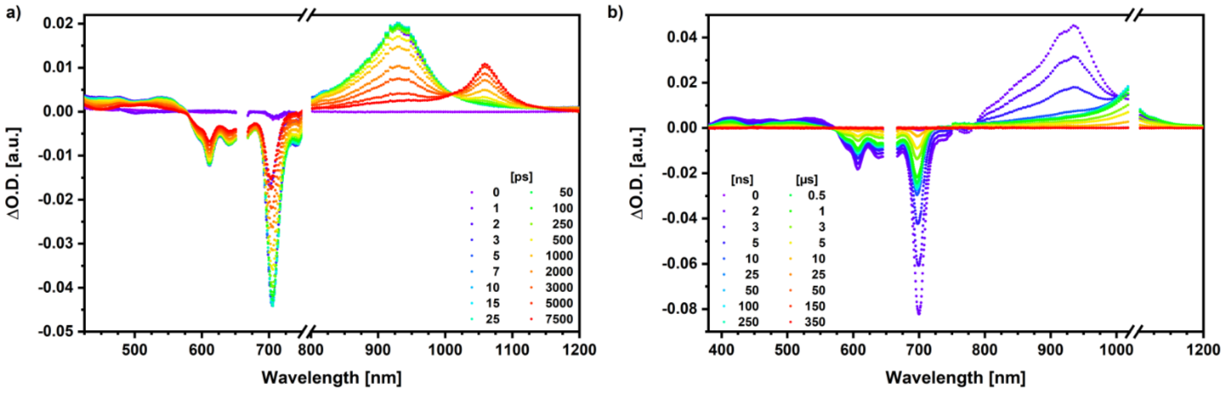

Figure S21: Femtosecond differential absorption spectra of SubPc **2** (1.5 x 10^{-5} M) in argon purged toluene at delay times between 0 and 7500 ps after 656 nm laser excitation (200 nJ) at room temperature. b) Nanosecond differential absorption spectra of SubPc **2** (1.5 x 10^{-5} M) in argon purged toluene at delay times between 0 and 350 µs after 656 nm laser excitation (450 nJ) at room temperature.

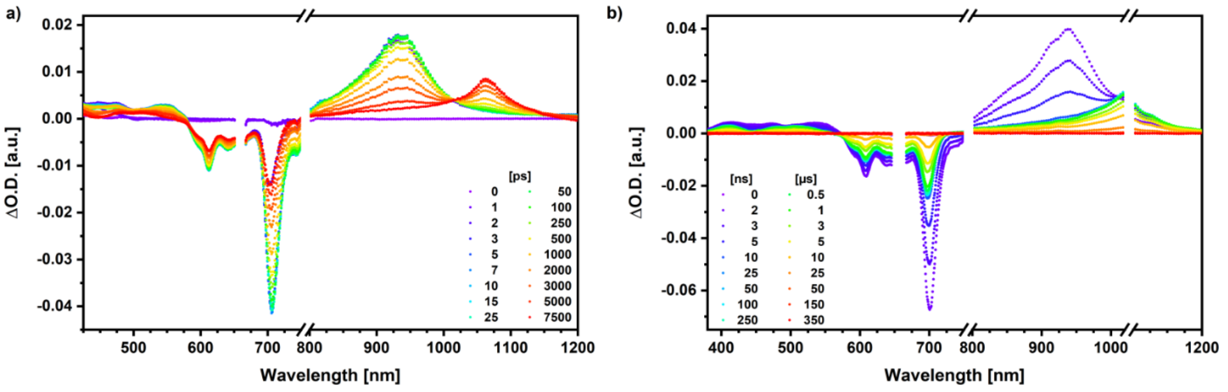

Figure S22: Femtosecond differential absorption spectra of SubPc **2** (1.5 x 10^{-5} M) in argon purged PhCl at delay times between 0 and 7500 ps after 656 nm laser excitation (200 nJ) at room temperature. b) Nanosecond differential absorption spectra of SubPc **2** (1.5 x 10^{-5} M) in argon purged PhCl at delay times between 0 and 350 µs after 656 nm laser excitation (450 nJ) at room temperature.

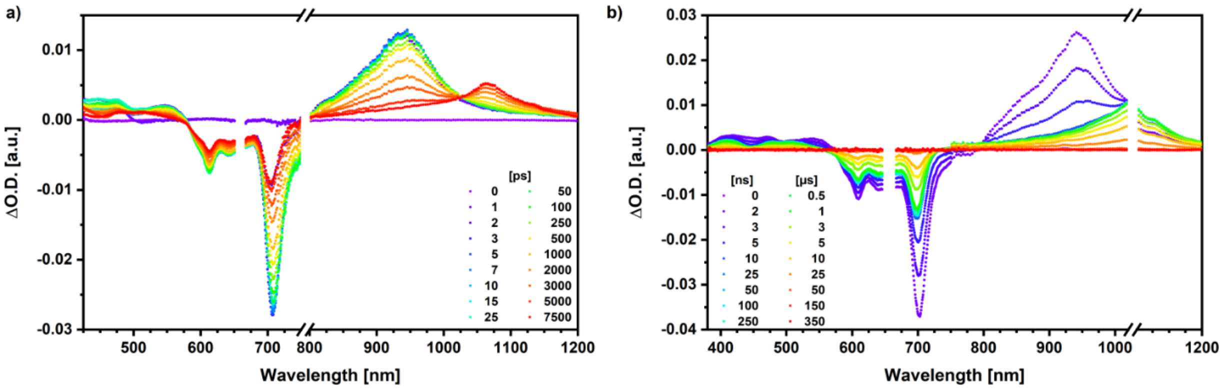

Figure S23: Femtosecond differential absorption spectra of SubPc **2** (1.5 x 10^{-5} M) in argon purged PhCN at delay times between 0 and 7500 ps after 656 nm laser excitation (200 nJ) at room temperature. b) Nanosecond differential absorption spectra of SubPc **2** (1.5 x 10^{-5} M) in argon purged PhCN at delay times between 0 and 350 µs after 656 nm laser excitation (450 nJ) at room temperature.

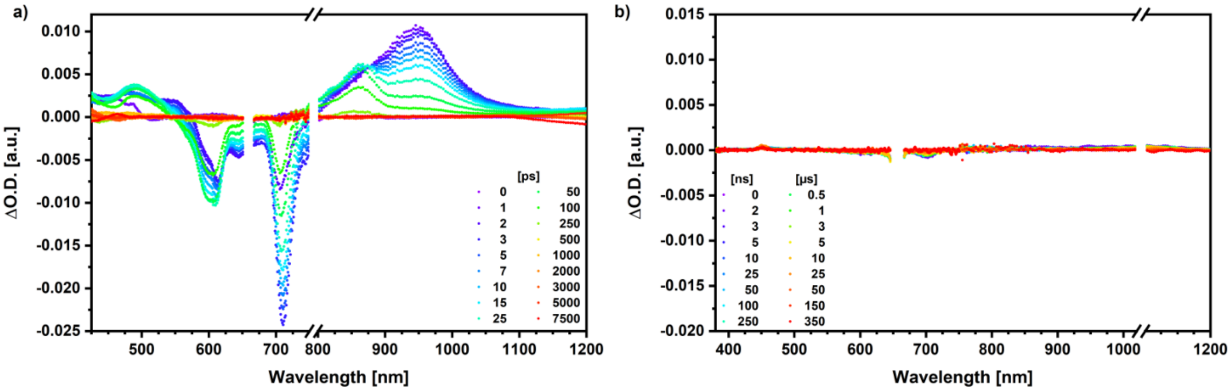

Figure S24: Femtosecond differential absorption spectra of *syn*-SubPc **1** (1.5 x 10^{-5} M) in argon purged PhCN at delay times between 0 and 7500 ps after 656 nm laser excitation (200 nJ) at room temperature. b) Nanosecond differential absorption spectra of *syn*-SubPc **1** (1.5 x 10^{-5} M) in argon purged PhCN at delay times between 0 and 350 µs after 656 nm laser excitation (450 nJ) at room temperature.

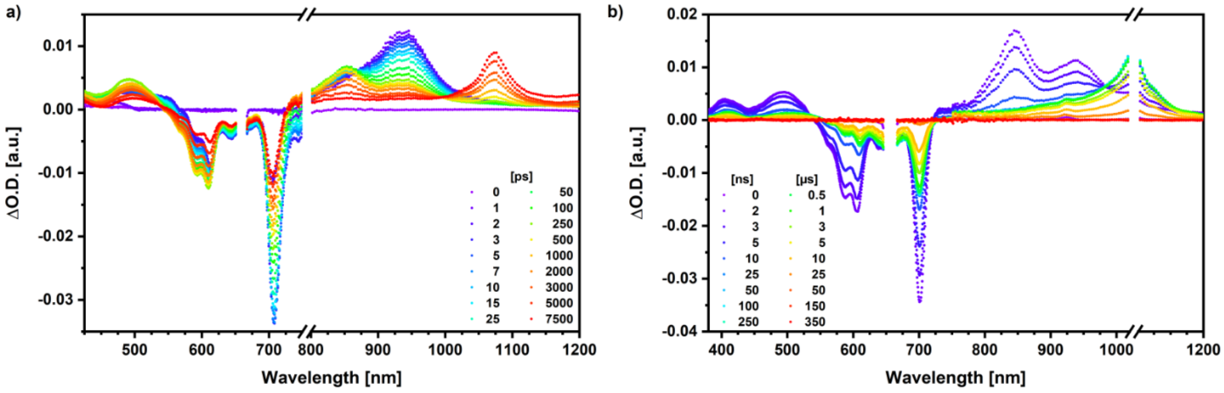

Figure S25: Femtosecond differential absorption spectra of *syn*-SubPc **1** (1.5 x 10^{-5} M) in argon purged toluene at delay times between 0 and 7500 ps after 656 nm laser excitation (200 nJ) at room temperature. b) Nanosecond differential absorption spectra of *syn*-SubPc **1** (1.5 x 10^{-5} M) in argon purged toluene at delay times between 0 and 350 µs after 656 nm laser excitation (450 nJ) at room temperature.

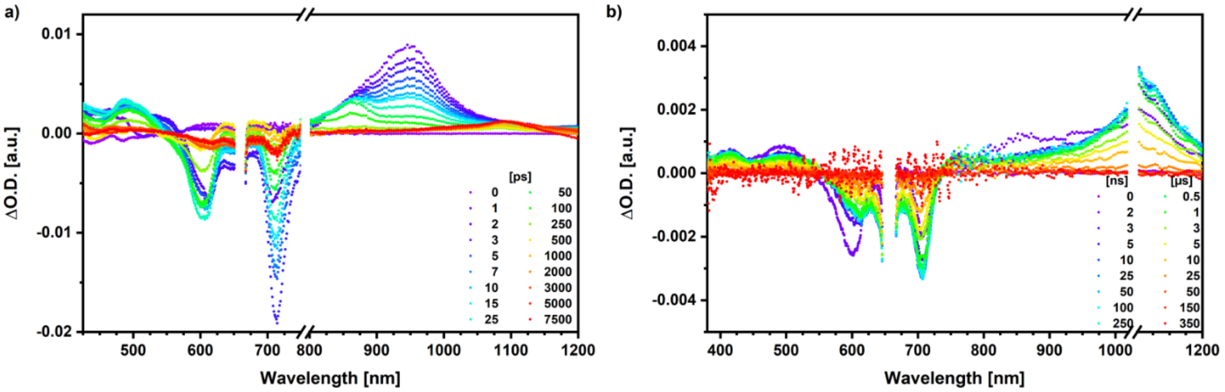

Figure S26: Femtosecond differential absorption spectra of *syn*-SubPc **1** (1.5×10^{-5} M) and C_{60} (3.0×10^{-4} M) in argon purged PhCN at delay times between 0 and 7500 ps after 656 nm laser excitation (200 nJ) at room temperature. b) Nanosecond differential absorption spectra of *syn*-SubPc **1** (1.5×10^{-5} M) and C_{60} (3.0×10^{-4} M) in argon purged PhCN at delay times between 0 and 350 µs after 656 nm laser excitation (450 nJ) at room temperature.

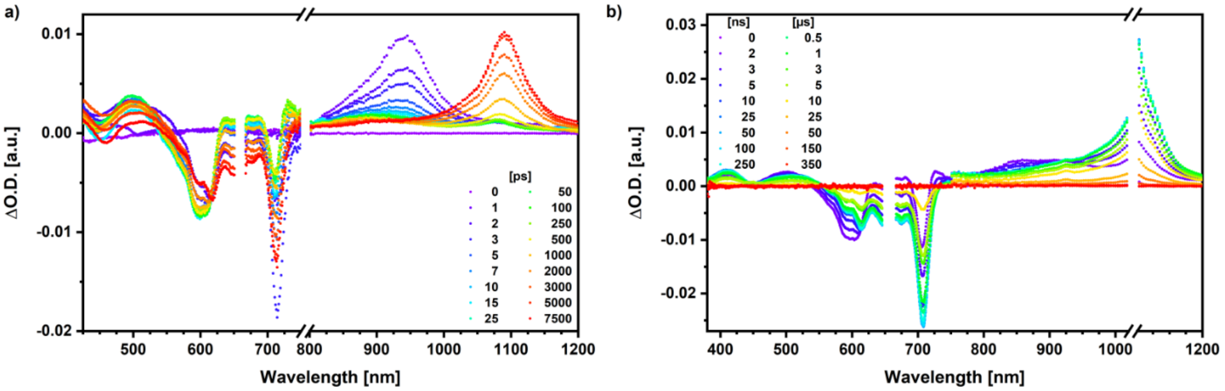

Figure S27: Femtosecond differential absorption spectra of *syn*-SubPc **1** (1.5×10^{-5} M) and C_{60} (3.0×10^{-4} M) in argon purged toluene at delay times between 0 and 7500 ps after 656 nm laser excitation (200 nJ) at room temperature. b) Nanosecond differential absorption spectra of *syn*-SubPc **1** (1.5×10^{-5} M) and C_{60} (3.0×10^{-4} M) in argon purged Toluene at delay times between 0 and 350 µs after 656 nm laser excitation (450 nJ) at room temperature.

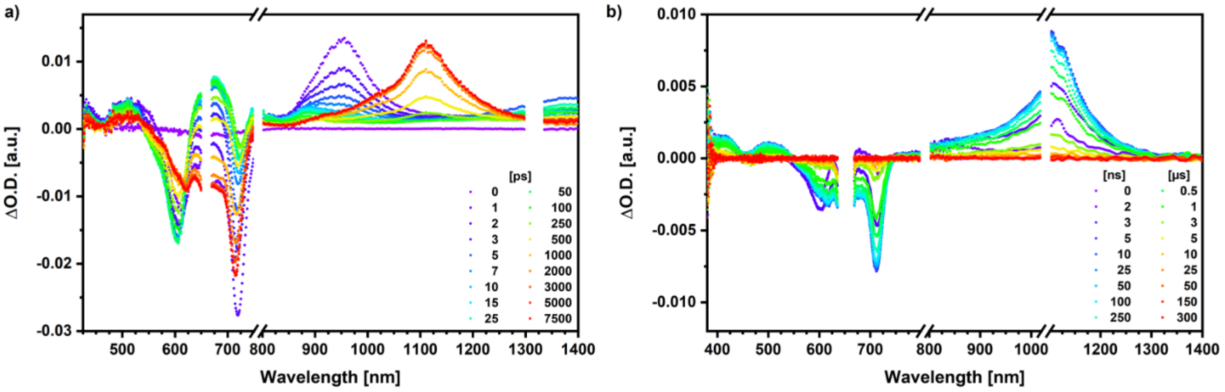

Figure S28: Femtosecond differential absorption spectra of *syn*-SubPc **1** (1.5×10^{-5} M) and C_{70} (3.0×10^{-4} M) in argon purged PhCN at delay times between 0 and 7500 ps after 656 nm laser excitation (300 nJ) at room temperature. b) Nanosecond differential absorption spectra of *syn*-SubPc **1** (1.5×10^{-5} M) and C_{70} (3.0×10^{-4} M) in argon purged PhCN at delay times between 0 and 350 µs after 656 nm laser excitation (300 nJ) at room temperature.

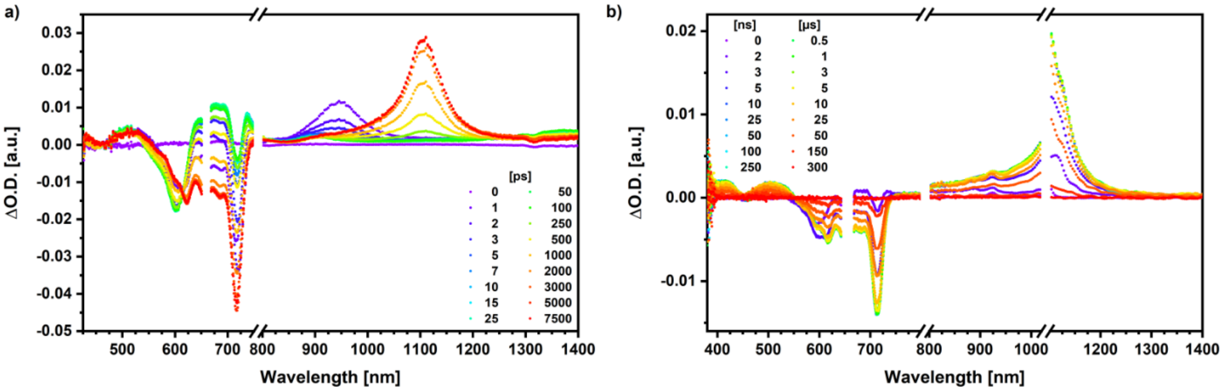

Figure S29: Femtosecond differential absorption spectra of *syn*-SubPc **1** (1.5 x 10^{-5} M) and C_{70} (3.0 x 10^{-4} M) in argon purged toluene at delay times between 0 and 7500 ps after 656 nm laser excitation (300 nJ) at room temperature. b) Nanosecond differential absorption spectra of *syn*-SubPc **1** (1.5 x 10^{-5} M)) and C_{70} (3.0 x 10^{-4} M) in argon purged toluene at delay times between 0 and 350 µs after 656 nm laser excitation (300 nJ) at room temperature.

6. Global Target Analysis

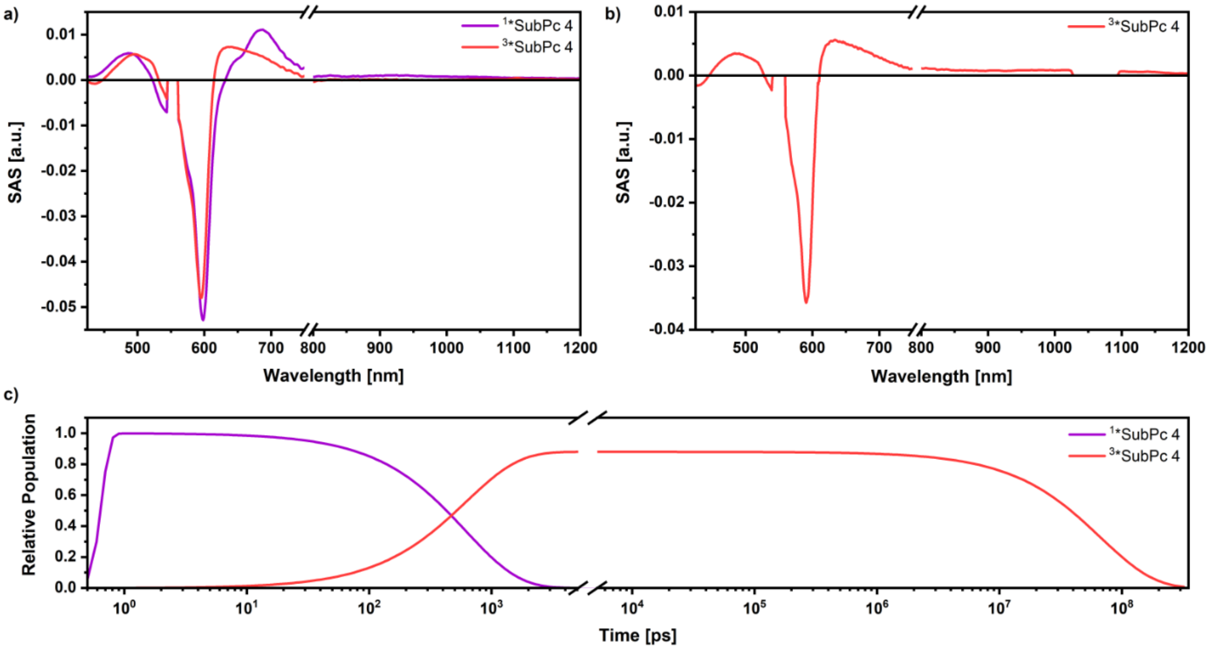

Figure S30: SAS obtained by Global Target Analysis of SubPc **4** in toluene after 550 nm laser excitation of a) femtosecond and b) nanosecond transient absorption data. c) Calculated population profiles (The break marks the crossing from the femtosecond to the nanosecond setup).

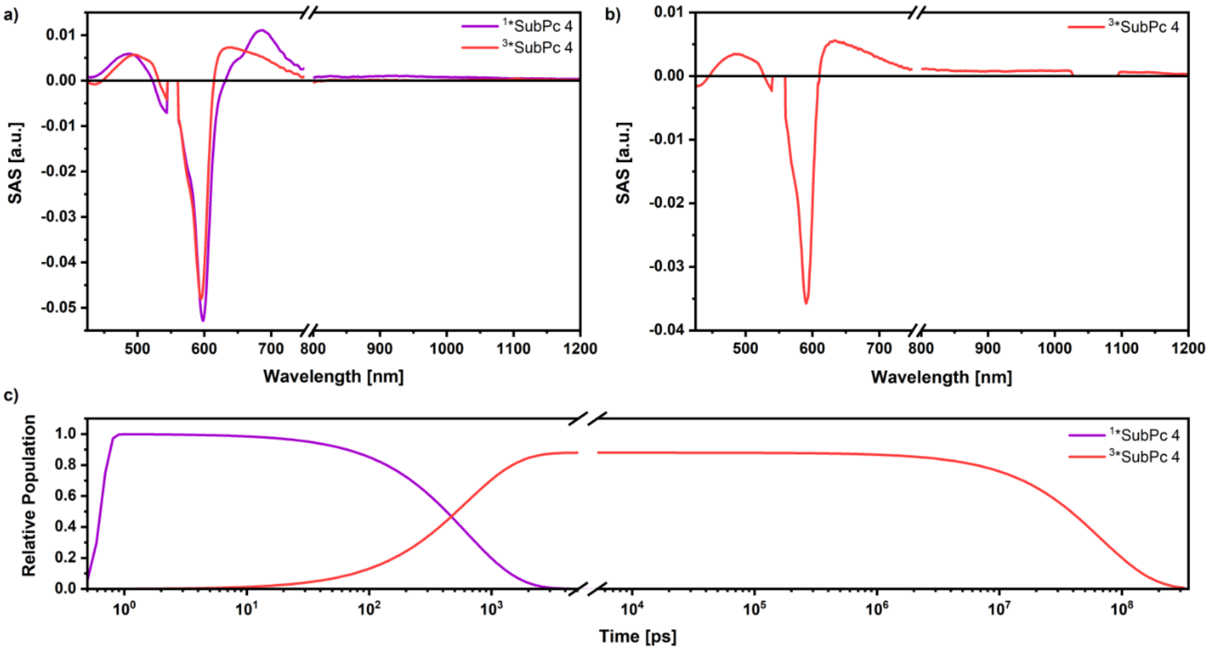

Figure S31: SAS obtained by Global Target Analysis of SubPc **4** in PhCl after 550 nm laser excitation of a) femtosecond and b) nanosecond transient absorption data. c) Calculated population profiles (The break marks the crossing from the femtosecond to the nanosecond setup).

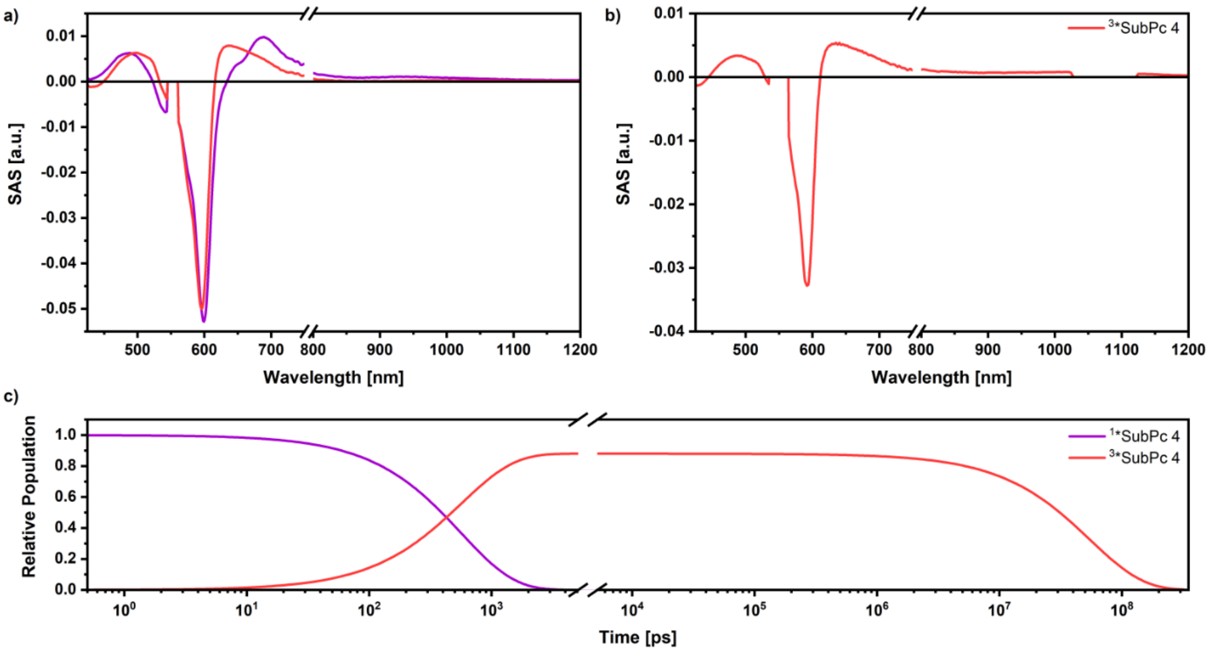

Figure S32: SAS obtained by Global Target Analysis of SubPc **4** in PhCN after 550 nm laser excitation of a) femtosecond and b) nanosecond transient absorption data. c) Calculated population profiles (The break marks the crossing from the femtosecond to the nanosecond setup).

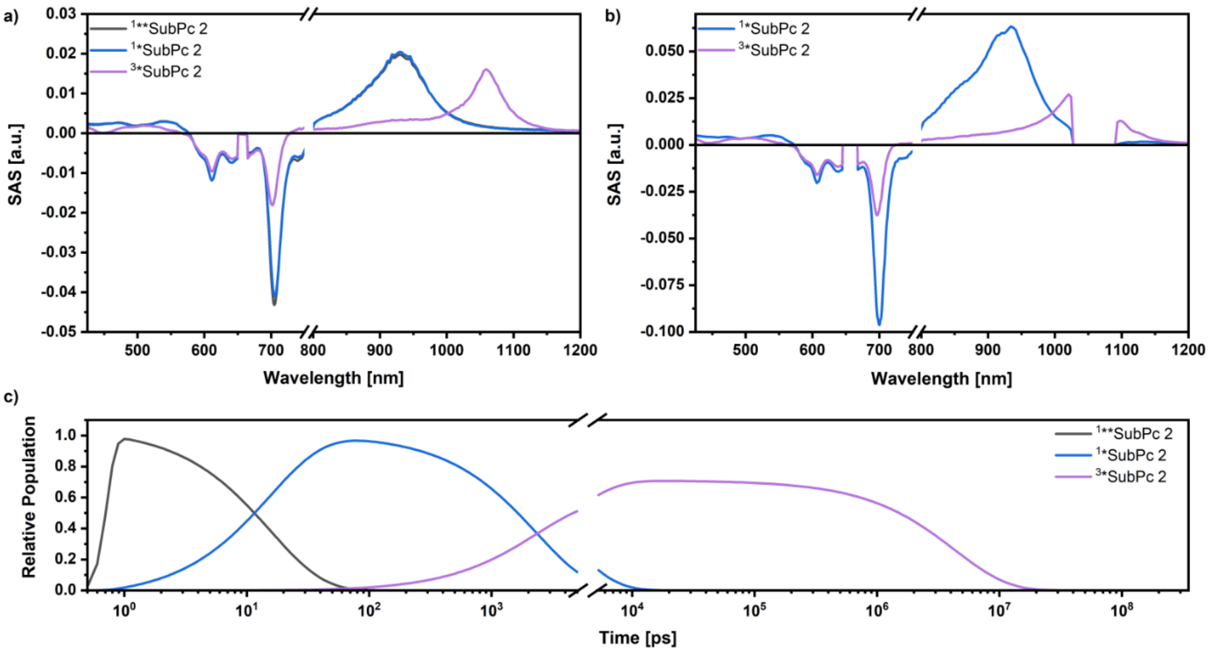

Figure S33: SAS obtained by Global Target Analysis of SubPc **2** in toluene after 656 nm laser excitation of a) femtosecond and b) nanosecond transient absorption data. c) Calculated population profiles (The break marks the crossing from the femtosecond to the nanosecond setup).

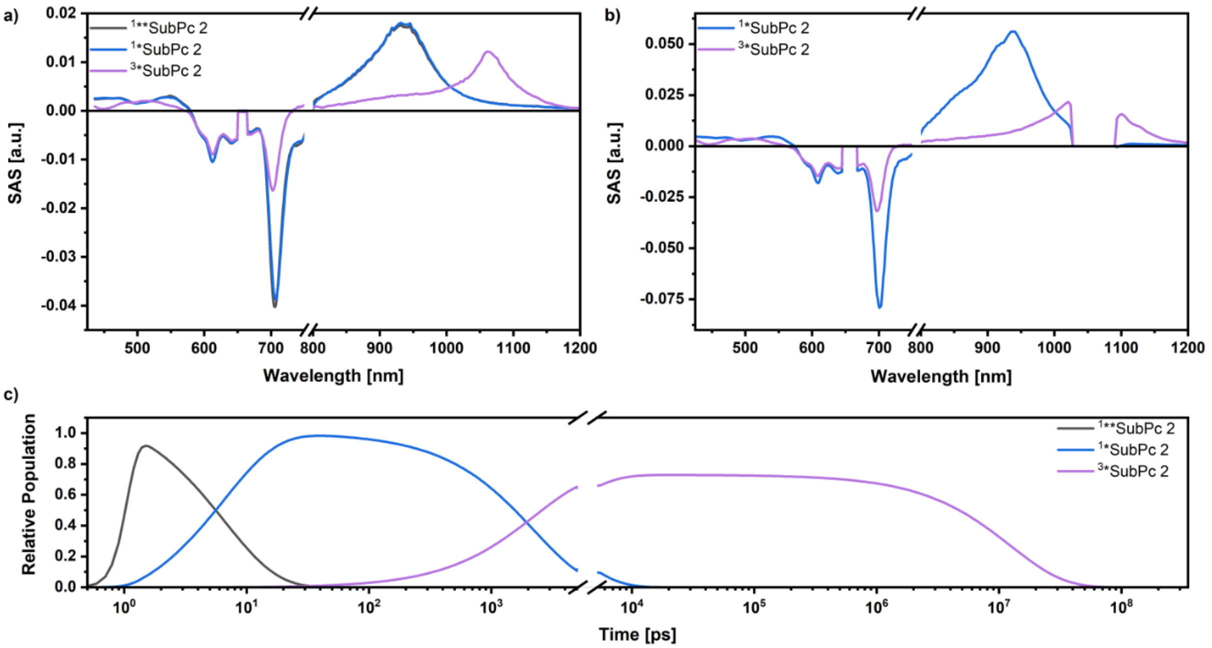

Figure S34: SAS obtained by Global Target Analysis of SubPc **2** in PhCl after 656 nm laser excitation of a) femtosecond and b) nanosecond transient absorption data. c) Calculated population profiles (The break marks the crossing from the femtosecond to the nanosecond setup).

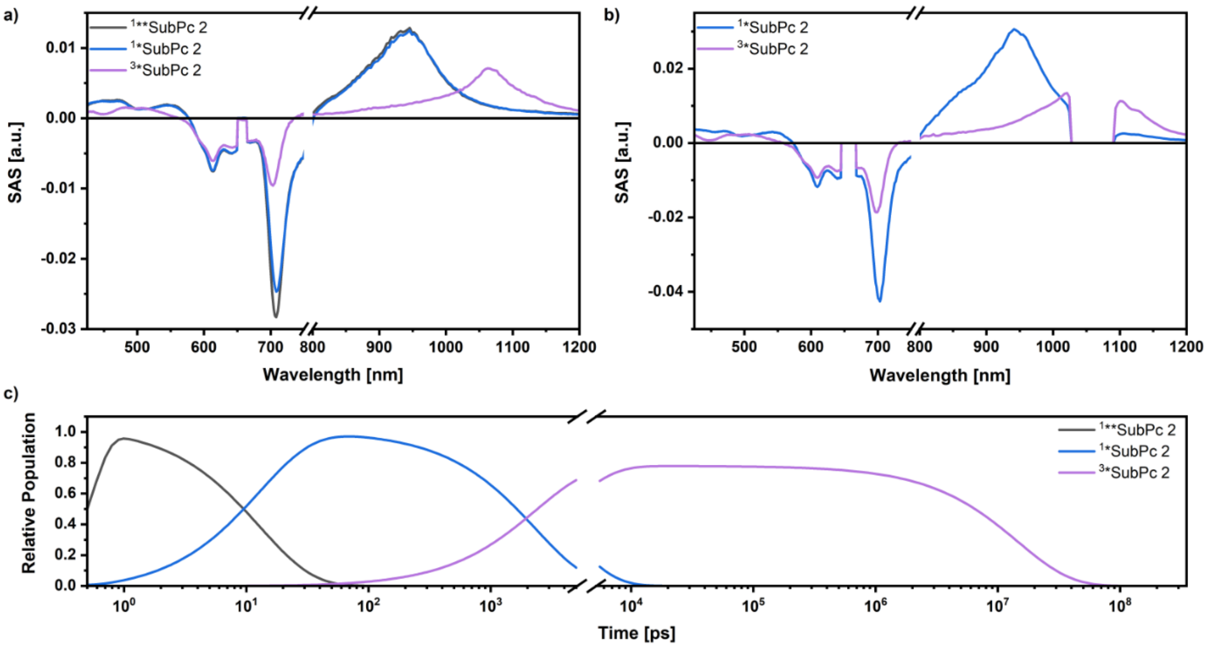

Figure S35: SAS obtained by Global Target Analysis of SubPc **2** in PhCN after 656 nm laser excitation of a) femtosecond and b) nanosecond transient absorption data. c) Calculated population profiles (The break marks the crossing from the femtosecond to the nanosecond setup).

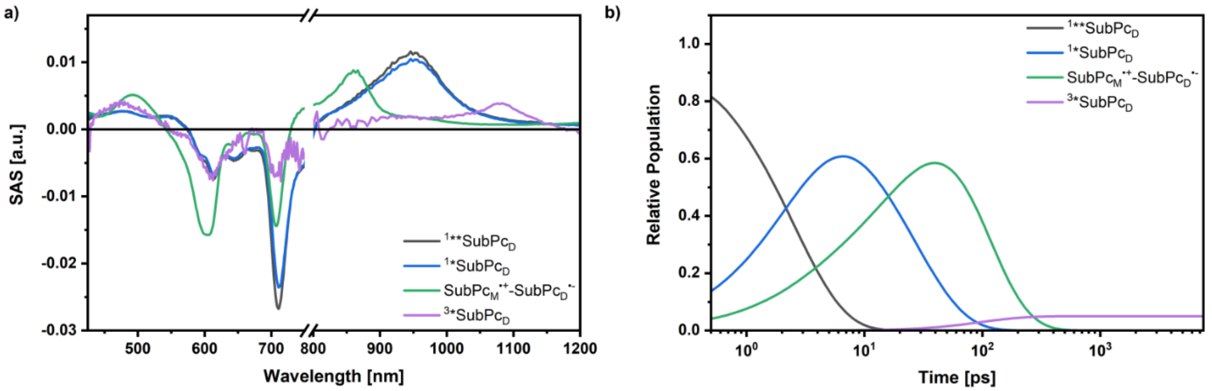

Figure S36: a) SAS obtained by Global Target Analysis of *syn*-SubPc **1** in PhCN after 656 nm laser excitation of femtosecond transient absorption data. b) Calculated population profiles.

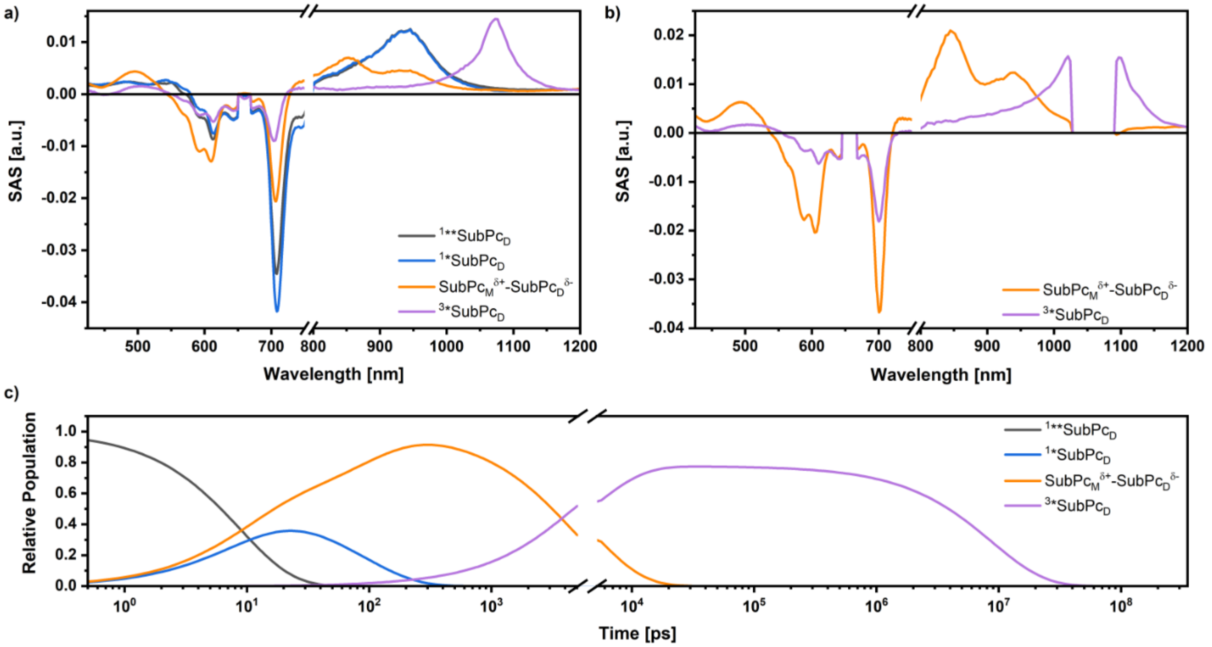

Figure S37: SAS obtained by Global Target Analysis of *syn*-SubPc **1** in toluene after 656 nm laser excitation of a) femtosecond and b) nanosecond transient absorption data. c) Calculated population profiles (The break marks the crossing from the femtosecond to the nanosecond setup).

7. Calculated Structures

SubPc 4

71

I	6.662621000	-2.370062000	-1.681211000
C	0.601659000	-2.364520000	3.922113000
C	-0.519112000	-1.574164000	3.629855000
C	-1.806297000	-2.097306000	3.819455000
C	-1.966633000	-3.400759000	4.293848000
C	-0.850211000	-4.191756000	4.581681000
C	0.432412000	-3.666933000	4.396318000
O	-0.357624000	-0.306847000	3.153931000
B	-0.290326000	-0.126510000	1.730643000
N	-1.497579000	-0.579493000	0.958286000
C	-2.514102000	0.272984000	0.622270000
N	-2.386524000	1.602746000	0.705637000
C	-1.173937000	2.100598000	0.981156000
N	-0.120656000	1.307562000	1.350060000
C	-0.586328000	3.397415000	0.664222000
C	0.832099000	3.245069000	0.719011000
C	1.104973000	1.855471000	1.068548000
C	-1.576172000	-1.802188000	0.343704000
C	-2.906407000	-1.844239000	-0.254497000
C	-3.489448000	-0.551962000	-0.083600000
N	2.195845000	1.107115000	0.875814000
N	-0.500817000	-2.568888000	0.143684000
C	0.700915000	-2.047779000	0.424988000
N	0.845700000	-0.835070000	1.041604000
C	2.042152000	-0.220596000	0.791699000
C	2.025836000	-2.378254000	-0.085030000
C	2.864598000	-1.236705000	0.142052000
C	-1.152341000	4.604577000	0.249053000
C	-0.317976000	5.674538000	-0.084695000
C	1.110372000	5.520862000	-0.030119000
C	1.671061000	4.300849000	0.357448000
C	-3.572492000	-2.829176000	-0.985363000
C	-4.831016000	-2.549760000	-1.525651000
C	-5.417494000	-1.248752000	-1.354886000
C	-4.732641000	-0.257403000	-0.646746000
C	4.183981000	-1.210997000	-0.321159000
C	4.656723000	-2.342615000	-0.983407000
C	3.843407000	-3.473051000	-1.212661000
C	2.520379000	-3.488697000	-0.776149000
S	-0.933399000	7.257275000	-0.595672000

C	-2.731520000	7.031844000	-0.534039000
S	2.084818000	6.931896000	-0.479957000
C	3.784424000	6.326323000	-0.298135000
S	-7.012974000	-0.988157000	-2.084056000
C	-7.376621000	0.736332000	-1.657646000
S	-5.773330000	-3.738620000	-2.443180000
C	-4.699436000	-5.199839000	-2.436744000
H	1.596822000	-1.941289000	3.772986000
H	-2.668724000	-1.468314000	3.590977000
H	-2.973200000	-3.801326000	4.438829000
H	1.311024000	-4.276681000	4.621820000
H	-2.236113000	4.686530000	0.192370000
H	2.748655000	4.150619000	0.383816000
H	-3.095620000	-3.798185000	-1.119743000
H	-5.144104000	0.742498000	-0.522501000
H	4.808944000	-0.331714000	-0.162964000
H	4.253378000	-4.335340000	-1.740011000
H	1.879180000	-4.351407000	-0.966242000
H	-8.365735000	0.938606000	-2.090987000
H	-6.644260000	1.426930000	-2.100309000
H	-7.426172000	0.883023000	-0.568955000
H	-0.979088000	-5.211338000	4.951867000
H	4.424528000	7.177557000	-0.567701000
H	3.992941000	5.491182000	-0.982344000
H	4.000363000	6.030392000	0.738632000
H	-3.154501000	7.998698000	-0.839538000
H	-3.075615000	6.796042000	0.483420000
H	-3.067785000	6.256214000	-1.237303000
H	-5.252253000	-5.966828000	-2.996325000
H	-4.516653000	-5.563899000	-1.415397000
H	-3.744787000	-5.003956000	-2.946017000

SubPc 2

76

N	5.167028000	1.182124000	1.017708000
B	4.807705000	-0.000003000	1.855161000
N	3.316419000	-0.000003000	1.889733000
C	2.579753000	1.148450000	1.783450000
N	3.116100000	2.307898000	1.392551000
C	4.374481000	2.294811000	0.942975000
C	6.177667000	1.144346000	0.094575000
N	6.734005000	-0.000003000	-0.309893000
C	6.177667000	-1.144351000	0.094573000

N	5.167027000	-1.182129000	1.017707000
C	2.579752000	-1.148455000	1.783448000
N	3.116099000	-2.307903000	1.392549000
C	4.374480000	-2.294817000	0.942973000
C	6.208378000	2.482353000	-0.484196000
C	5.081703000	3.200980000	0.045689000
C	1.186077000	0.723724000	1.862115000
C	1.186077000	-0.723729000	1.862124000
C	6.208376000	-2.482358000	-0.484199000
C	5.081705000	-3.200987000	0.045692000
C	7.024278000	3.068362000	-1.452034000
C	6.735802000	4.367189000	-1.876704000
C	5.632288000	5.070774000	-1.357930000
C	4.794591000	4.489854000	-0.403563000
C	0.000074000	1.456590000	1.832438000
C	-1.185913000	0.723732000	1.861988000
C	-1.185914000	-0.723735000	1.861996000
C	0.000072000	-1.456594000	1.832456000
C	7.024270000	-3.068364000	-1.452042000
C	6.735795000	-4.367192000	-1.876711000
C	5.632287000	-5.070780000	-1.357931000
C	4.794594000	-4.489862000	-0.403559000
C	-2.579593000	-1.148455000	1.783223000
N	-3.316249000	-0.000001000	1.889621000
C	-2.579592000	1.148455000	1.783228000
N	-3.115975000	2.307877000	1.392373000
C	-4.374422000	2.294803000	0.942934000
N	-5.166946000	1.182132000	1.017770000
B	-4.807515000	0.000001000	1.855130000
N	-5.166949000	-1.182127000	1.017768000
C	-4.374425000	-2.294799000	0.942926000
N	-3.115977000	-2.307875000	1.392362000
C	-6.177735000	-1.144341000	0.094768000
N	-6.734135000	0.000005000	-0.309619000
C	-6.177733000	1.144349000	0.094770000
C	-5.081642000	3.200900000	0.045575000
C	-6.208497000	2.482336000	-0.484023000
C	-5.081653000	-3.200897000	0.045574000
C	-6.208508000	-2.482330000	-0.484020000
C	-4.794458000	4.489693000	-0.403885000
C	-5.632281000	5.070610000	-1.358133000
C	-6.736011000	4.367111000	-1.876579000
C	-7.024543000	3.068360000	-1.451732000
C	-4.794474000	-4.489690000	-0.403887000

C	-5.632302000	-5.070606000	-1.358133000
C	-6.736032000	-4.367105000	-1.876574000
C	-7.024559000	-3.068352000	-1.451726000
F	5.397551000	6.302501000	-1.789081000
F	7.505375000	4.958919000	-2.779745000
F	3.756884000	5.175240000	0.054870000
F	8.063601000	2.430693000	-1.970048000
F	8.063591000	-2.430694000	-1.970061000
F	7.505365000	-4.958920000	-2.779757000
F	5.397549000	-6.302507000	-1.789081000
F	3.756889000	-5.175249000	0.054876000
F	-3.756656000	-5.175047000	0.054343000
F	-5.397541000	-6.302278000	-1.789435000
F	-7.505742000	-4.958843000	-2.779493000
F	-8.064017000	-2.430717000	-1.969520000
F	-8.064002000	2.430729000	-1.969527000
F	-7.505717000	4.958853000	-2.779499000
F	-5.397517000	6.302283000	-1.789434000
F	-3.756640000	5.175048000	0.054349000
Cl	-5.596463000	0.000003000	3.529520000
Cl	5.596798000	-0.000001000	3.529325000
H	0.000093000	2.546687000	1.789171000
H	0.000091000	-2.546691000	1.789194000

***syn*-SubPc 1 (face-to-face)**
218

B	5.048014000	-4.732675000	-0.807586000
N	5.235927000	-3.256758000	-0.561390000
C	5.408491000	-2.740591000	0.689814000
N	5.765380000	-3.501278000	1.729720000
C	6.103036000	-4.771874000	1.480418000
N	5.916317000	-5.357745000	0.259689000
C	6.964559000	-5.693213000	2.213376000
C	7.359857000	-6.726455000	1.295638000
C	6.734318000	-6.424246000	0.012815000
C	5.363461000	-2.349440000	-1.571065000
C	5.383800000	-1.044498000	-0.915354000
C	5.399356000	-1.291105000	0.511634000
N	7.040336000	-6.820968000	-1.225833000
N	5.654565000	-2.716538000	-2.823269000
C	5.972041000	-3.998623000	-3.035698000
N	5.830275000	-4.965678000	-2.077786000

C	6.658467000	-6.042988000	-2.242616000
C	6.785891000	-4.613920000	-4.077851000
C	7.215124000	-5.892366000	-3.582167000
C	7.487533000	-5.662717000	3.506029000
C	8.380823000	-6.663715000	3.893338000
C	8.767607000	-7.675827000	2.994681000
C	8.268932000	-7.707813000	1.690603000
C	5.428757000	0.248505000	-1.435396000
C	5.433495000	-0.247781000	1.436036000
C	8.096009000	-6.676258000	-4.327638000
C	8.531542000	-6.203020000	-5.567004000
C	8.111136000	-4.950120000	-6.052624000
C	7.246579000	-4.145234000	-5.308372000
F	8.877862000	-6.674342000	5.123156000
F	9.616652000	-8.608052000	3.406519000
F	9.351252000	-6.935298000	-6.309524000
B	5.047358000	4.733139000	0.809341000
N	5.235595000	3.257352000	0.562605000
C	5.366927000	2.350152000	1.571897000
N	5.661727000	2.717519000	2.823157000
C	5.978939000	3.999842000	3.034535000
N	5.833424000	4.966776000	2.077052000
C	6.795661000	4.615756000	4.074071000
C	7.222401000	5.894495000	3.576984000
C	6.661376000	6.044677000	2.239212000
C	5.404493000	2.741280000	-0.689127000
C	5.396889000	1.291800000	-0.510891000
C	5.386042000	1.045196000	0.916149000
N	7.039473000	6.822887000	1.221191000
N	5.757514000	3.502188000	-1.730197000
C	6.095062000	4.773028000	-1.482007000
N	5.911804000	5.358807000	-0.260709000
C	6.729822000	6.425900000	-0.016466000
C	6.953598000	5.694959000	-2.217724000
C	7.351070000	6.728512000	-1.301280000
C	7.260601000	4.147443000	5.303133000
C	8.126945000	4.952972000	6.044607000
C	8.544909000	6.206155000	5.557613000
C	8.105094000	6.679039000	4.319625000
C	8.258188000	7.710505000	-1.699157000
C	8.752747000	7.678825000	-3.004808000
C	8.363835000	6.666406000	-3.902201000
C	7.472489000	5.664787000	-3.512030000
F	8.568310000	4.545576000	7.227359000

F	9.599821000	8.611636000	-3.419370000
F	8.856966000	6.677347000	-5.133589000
F	7.137016000	4.719177000	-4.378606000
F	8.654825000	8.671467000	-0.877011000
F	6.896653000	2.967336000	5.785475000
F	7.154142000	-4.717372000	4.373700000
F	8.663646000	-8.668466000	0.867171000
F	8.521207000	-7.854952000	-3.895808000
F	8.548446000	-4.542376000	-7.236761000
F	6.880253000	-2.965373000	-5.789510000
C	3.540564000	-5.164233000	-0.778093000
C	3.539510000	5.163616000	0.784197000
C	2.347024000	5.364992000	0.613710000
C	2.348510000	-5.366244000	-0.605382000
B	-4.327615000	-4.238709000	2.334661000
N	-5.479716000	-4.774084000	1.548961000
C	-6.636329000	-4.064728000	1.395469000
N	-6.696760000	-2.745167000	1.630795000
C	-5.547840000	-2.103904000	1.892869000
N	-4.374790000	-2.772640000	2.098480000
C	-5.160316000	-0.697800000	1.801045000
C	-3.729847000	-0.648038000	1.803520000
C	-3.246989000	-2.023223000	1.904353000
C	-5.405792000	-5.910392000	0.790832000
C	-6.758673000	-6.100617000	0.275031000
C	-7.531768000	-4.965568000	0.674734000
N	-2.056941000	-2.585612000	1.657625000
N	-4.238190000	-6.479289000	0.475446000
C	-3.111912000	-5.836416000	0.815169000
N	-3.126136000	-4.698811000	1.572142000
C	-2.022950000	-3.908200000	1.433329000
C	-1.742438000	-5.940103000	0.324334000
C	-1.051797000	-4.747648000	0.736985000
C	-3.051331000	6.519707000	-4.263528000
C	-4.284178000	5.879137000	-4.072693000
C	-5.474283000	6.605911000	-4.222148000
C	-5.427302000	7.960734000	-4.556719000
C	-4.198803000	8.601656000	-4.743773000
C	-3.013275000	7.874774000	-4.598235000
O	-4.324468000	4.560531000	-3.730481000
C	-5.907260000	0.476802000	1.728776000
C	-5.246550000	1.709693000	1.730358000
C	-3.814552000	1.758609000	1.705171000
C	-3.068387000	0.576270000	1.713048000

C	-7.314886000	-7.076420000	-0.551355000
C	-8.645796000	-6.952956000	-0.964027000
C	-9.426725000	-5.823802000	-0.545429000
C	-8.860994000	-4.842366000	0.272958000
C	0.286521000	-4.552136000	0.412707000
C	0.959295000	-5.541150000	-0.329384000
C	0.258222000	-6.697259000	-0.769645000
C	-1.082678000	-6.896297000	-0.455189000
B	-4.327350000	4.235916000	-2.327874000
N	-5.480746000	4.772091000	-1.544768000
C	-6.638134000	4.063473000	-1.393760000
N	-6.698925000	2.743959000	-1.629534000
C	-5.549870000	2.101936000	-1.889152000
N	-4.375884000	2.769939000	-2.091810000
C	-5.163390000	0.695558000	-1.796794000
C	-3.732919000	0.645035000	-1.795302000
C	-3.248973000	2.019898000	-1.895126000
C	-5.407605000	5.908334000	-0.786521000
C	-6.761454000	6.099488000	-0.273646000
C	-7.534503000	4.964930000	-0.674938000
N	-2.058958000	2.581647000	-1.646430000
N	-4.240259000	6.476695000	-0.468964000
C	-3.113687000	5.833190000	-0.806422000
N	-3.127318000	4.695166000	-1.562723000
C	-2.024612000	3.904247000	-1.422397000
C	-1.744526000	5.936914000	-0.314509000
C	-1.053781000	4.744050000	-0.725900000
C	-5.911197000	-0.478675000	-1.727571000
C	-5.251117000	-1.711931000	-1.727652000
C	-3.819257000	-1.761558000	-1.696960000
C	-3.072446000	-0.579642000	-1.702548000
C	-7.319028000	7.076205000	0.550771000
C	-8.651265000	6.954374000	0.959570000
C	-9.432182000	5.825787000	0.539295000
C	-8.864968000	4.843170000	-0.276663000
C	0.284567000	4.549101000	-0.401616000
C	0.957400000	5.539065000	0.339240000
C	0.256149000	6.695354000	0.778604000
C	-1.084872000	6.893844000	0.464143000
C	-5.480472000	-6.607872000	4.226320000
C	-4.289536000	-5.881893000	4.079646000
C	-3.057572000	-6.523251000	4.273500000
C	-3.021208000	-7.878305000	4.608445000
C	-4.207559000	-8.604396000	4.751216000

C	-5.435184000	-7.962692000	4.561143000
O	-4.328149000	-4.563278000	3.737263000
F	8.528075000	7.858022000	3.886408000
F	9.366455000	6.939046000	6.297494000
S	-6.095257000	3.261047000	1.831658000
C	-7.825549000	2.743219000	1.961533000
S	-3.070818000	3.367774000	1.710826000
C	-1.332861000	2.991162000	2.055491000
S	-11.090930000	-5.725626000	-1.155014000
C	-11.725503000	-4.226366000	-0.355407000
S	-9.449040000	-8.141603000	-2.006016000
C	-8.117563000	-9.313192000	-2.384878000
S	-6.100134000	-3.262752000	-1.833933000
C	-7.829855000	-2.744013000	-1.968152000
S	-3.076106000	-3.371073000	-1.698668000
C	-1.337771000	-2.995698000	-2.043114000
S	-11.098710000	5.730546000	1.142919000
C	-11.732261000	4.231013000	0.343041000
S	-9.456403000	8.144638000	1.998230000
C	-8.125259000	9.316034000	2.378859000
H	5.455514000	0.434420000	-2.510095000
H	5.463801000	-0.433684000	2.510643000
H	-2.133973000	5.939646000	-4.146285000
H	-6.426227000	6.092908000	-4.072822000
H	-6.359333000	8.520164000	-4.670864000
H	-2.048334000	8.366675000	-4.745104000
H	-6.992994000	0.412632000	1.717776000
H	-1.980948000	0.591240000	1.679039000
H	-6.697155000	-7.916784000	-0.862216000
H	-9.427309000	-3.966919000	0.583786000
H	0.812577000	-3.652474000	0.734327000
H	0.799541000	-7.442480000	-1.354794000
H	-1.612517000	-7.788384000	-0.793836000
H	-6.996933000	-0.413934000	-1.720438000
H	-1.985136000	-0.595241000	-1.664758000
H	-6.701360000	7.916156000	0.862864000
H	-9.431318000	3.968163000	-0.588693000
H	0.810827000	3.649358000	-0.722675000
H	0.797436000	7.441343000	1.362804000
H	-1.614747000	7.786256000	0.801874000
H	-6.431723000	-6.094264000	4.074673000
H	-2.139561000	-5.943795000	4.158394000
H	-2.056947000	-8.370825000	4.757686000
H	-6.367853000	-8.521496000	4.673110000

H	-12.777066000	4.147363000	0.672244000
H	-11.184668000	3.333307000	0.664984000
H	-11.707336000	4.316471000	-0.752929000
H	-4.165502000	9.661905000	-5.004470000
H	-11.175700000	-3.328901000	-0.674228000
H	-11.704541000	-4.313298000	0.740528000
H	-12.769035000	-4.141023000	-0.688191000
H	-4.175576000	-9.664638000	5.012106000
H	-7.971468000	2.073228000	2.820559000
H	-8.160003000	2.264267000	1.030395000
H	-8.398212000	3.665053000	2.119468000
H	-0.844911000	-3.969007000	-2.158943000
H	-0.873131000	-2.461929000	-1.203450000
H	-1.240567000	-2.419677000	-2.974150000
H	-8.166599000	-2.265205000	-1.037770000
H	-8.402663000	-3.665432000	-2.128074000
H	-7.973171000	-2.073491000	-2.827213000
H	-8.569012000	-10.056088000	-3.056655000
H	-7.755492000	-9.823117000	-1.480517000
H	-7.282089000	-8.820971000	-2.903241000
H	-0.839460000	3.964126000	2.171909000
H	-0.868404000	2.457519000	1.215638000
H	-1.236101000	2.414615000	2.986252000
H	-8.578055000	10.060192000	3.048326000
H	-7.760514000	9.824389000	1.474688000
H	-7.291437000	8.824127000	2.900172000

syn-SubPc 1 (face-to-back)

218

B	-4.538514000	-4.748977000	-1.237922000
N	-4.922889000	-3.323150000	-0.940286000
C	-5.504934000	-2.521445000	-1.872299000
N	-6.154596000	-3.031718000	-2.924677000
C	-6.315097000	-4.361275000	-2.968133000
N	-5.667375000	-5.215576000	-2.117627000
C	-7.338924000	-5.177581000	-3.611262000
C	-7.327019000	-6.462371000	-2.966252000
C	-6.293959000	-6.418449000	-1.937138000
C	-4.675389000	-2.735545000	0.260721000
C	-4.928492000	-1.311733000	0.044495000
C	-5.430116000	-1.173375000	-1.309933000
N	-6.117702000	-7.165521000	-0.843022000
N	-4.447915000	-3.461632000	1.361968000

C	-4.603544000	-4.789704000	1.270032000
N	-4.790235000	-5.433602000	0.078414000
C	-5.435998000	-6.634477000	0.176797000
C	-4.919269000	-5.786353000	2.286245000
C	-5.450850000	-6.935617000	1.604690000
C	-8.295857000	-4.888017000	-4.584011000
C	-9.221776000	-5.873937000	-4.930656000
C	-9.209813000	-7.132149000	-4.299282000
C	-8.271423000	-7.430123000	-3.308968000
C	-4.775000000	-0.208302000	0.882137000
C	-5.730386000	0.073481000	-1.855679000
C	-5.933093000	-8.022131000	2.335096000
C	-5.870851000	-7.976550000	3.729592000
C	-5.342403000	-6.854265000	4.396499000
C	-4.878210000	-5.751042000	3.679114000
F	-10.129572000	-5.635933000	-5.868098000
F	-10.106843000	-8.039313000	-4.662010000
F	-6.301808000	-9.003768000	4.449301000
B	-4.957696000	4.720691000	-0.070490000
N	-5.137748000	3.226970000	-0.184662000
C	-5.602477000	2.616916000	-1.310190000
N	-6.234310000	3.296351000	-2.273517000
C	-6.530282000	4.578932000	-2.036414000
N	-6.057716000	5.263891000	-0.947871000
C	-7.590060000	5.419055000	-2.582459000
C	-7.792461000	6.500862000	-1.659540000
C	-6.853843000	6.309128000	-0.559881000
C	-4.930968000	2.390245000	0.872662000
C	-5.024295000	1.042769000	0.317385000
C	-5.473678000	1.184715000	-1.051841000
N	-6.889208000	6.765723000	0.695223000
N	-4.922740000	2.830467000	2.135735000
C	-5.278074000	4.103335000	2.348679000
N	-5.453672000	4.996498000	1.330212000
C	-6.253609000	6.059030000	1.634739000
C	-5.835234000	4.761920000	3.525823000
C	-6.446799000	5.984349000	3.079507000
C	-8.420277000	5.277840000	-3.694565000
C	-9.431707000	6.217744000	-3.901098000
C	-9.630013000	7.278276000	-2.996496000
C	-8.821027000	7.420201000	-1.867300000
C	-7.147015000	6.788995000	3.977797000
C	-7.224269000	6.393513000	5.315282000
C	-6.626277000	5.196690000	5.751988000

C	-5.938995000	4.370227000	4.860072000
F	-10.222783000	6.123982000	-4.961862000
F	-7.865409000	7.148734000	6.197260000
F	-6.722782000	4.861461000	7.031636000
F	-5.398521000	3.243396000	5.302576000
F	-7.732151000	7.916078000	3.598734000
F	-8.269085000	4.284188000	-4.559416000
F	-8.342996000	-3.709695000	-5.190031000
F	-8.296378000	-8.620451000	-2.726944000
F	-6.439899000	-9.093451000	1.742527000
F	-5.291022000	-6.860438000	5.721429000
F	-4.383108000	-4.705733000	4.334187000
C	-3.074674000	-4.780689000	-1.801567000
C	-3.465387000	5.147895000	-0.293214000
C	-2.247922000	5.202137000	-0.196745000
C	-1.971692000	-4.348902000	-2.104562000
B	4.195695000	-0.594062000	-0.782527000
N	5.053043000	-0.226452000	-1.957288000
C	6.418059000	-0.298796000	-1.934913000
N	7.065256000	-0.984078000	-0.982836000
C	6.346260000	-1.699618000	-0.108771000
N	4.979163000	-1.622569000	-0.039390000
C	6.705109000	-2.892552000	0.649691000
C	5.487938000	-3.567410000	0.967364000
C	4.391996000	-2.766411000	0.443814000
C	4.529788000	0.011677000	-3.203763000
C	5.669710000	0.419736000	-4.016155000
C	6.850287000	0.215557000	-3.232269000
N	3.130609000	-3.111536000	0.164722000
N	3.287301000	-0.351562000	-3.540203000
C	2.593576000	-1.087368000	-2.662464000
N	3.079390000	-1.365849000	-1.416232000
C	2.500506000	-2.434214000	-0.800986000
C	1.403134000	-1.919104000	-2.791501000
C	1.331306000	-2.746836000	-1.617045000
C	3.758394000	5.808398000	4.244012000
C	4.679179000	4.751589000	4.198277000
C	6.050900000	5.029902000	4.110486000
C	6.492966000	6.353601000	4.065590000
C	5.575873000	7.408210000	4.107074000
C	4.208881000	7.129245000	4.199155000
O	4.241183000	3.461550000	4.225661000
C	7.933421000	-3.472921000	0.974343000
C	7.960660000	-4.710085000	1.625060000

C	6.734435000	-5.396661000	1.917292000
C	5.509164000	-4.826499000	1.566376000
C	5.760993000	0.879745000	-5.329873000
C	7.018829000	1.173571000	-5.865047000
C	8.202876000	0.960076000	-5.083003000
C	8.110577000	0.463695000	-3.778711000
C	0.268162000	-3.622427000	-1.418338000
C	-0.754170000	-3.661832000	-2.384670000
C	-0.644685000	-2.888202000	-3.574133000
C	0.426364000	-2.027921000	-3.786114000
B	3.970669000	2.819678000	2.966321000
N	5.135873000	2.712281000	2.031443000
C	5.956173000	1.619824000	2.029649000
N	5.617603000	0.468874000	2.627743000
C	4.365918000	0.349767000	3.090087000
N	3.506229000	1.414076000	3.140430000
C	3.522697000	-0.825968000	3.307230000
C	2.165398000	-0.381617000	3.246257000
C	2.185242000	1.066075000	3.090300000
C	5.343933000	3.580689000	0.994588000
C	6.580493000	3.124819000	0.366601000
C	6.985862000	1.926703000	1.039715000
N	1.226554000	1.919749000	2.716805000
N	4.416858000	4.458511000	0.594987000
C	3.190866000	4.361727000	1.124891000
N	2.911771000	3.473138000	2.128945000
C	1.604300000	3.089902000	2.190275000
C	1.886315000	4.817357000	0.650452000
C	0.898617000	4.017262000	1.316771000
C	3.798965000	-2.180482000	3.501760000
C	2.740753000	-3.088291000	3.610412000
C	1.387990000	-2.657725000	3.413392000
C	1.112936000	-1.296910000	3.246190000
C	7.307992000	3.591485000	-0.727126000
C	8.461636000	2.907939000	-1.129792000
C	8.907072000	1.758635000	-0.405204000
C	8.151172000	1.264653000	0.663560000
C	-0.450118000	4.145973000	1.016702000
C	-0.847858000	5.102756000	0.064983000
C	0.130115000	5.898836000	-0.588121000
C	1.487061000	5.750816000	-0.312390000
C	3.117903000	2.319739000	-1.427512000
C	2.769031000	1.278594000	-0.559300000
C	1.418781000	0.962602000	-0.356009000

C	0.428516000	1.664556000	-1.043108000
C	0.774732000	2.684720000	-1.933563000
C	2.119604000	3.015431000	-2.112066000
O	3.740703000	0.514613000	0.033142000
F	-9.043538000	8.423012000	-1.030085000
F	-10.601553000	8.149433000	-3.234328000
S	9.467673000	-5.514659000	2.101413000
C	10.729079000	-4.286395000	1.663050000
S	6.848035000	-6.956433000	2.758546000
C	5.208424000	-7.682243000	2.459329000
S	9.744086000	1.412955000	-5.830885000
C	10.971390000	0.726155000	-4.685234000
S	7.244044000	1.827357000	-7.498254000
C	5.540852000	2.040201000	-8.085281000
S	3.007329000	-4.800733000	4.015097000
C	4.583671000	-4.736620000	4.921319000
S	0.122840000	-3.901291000	3.421536000
C	-1.393380000	-2.940572000	3.150288000
S	10.423311000	0.993607000	-0.926728000
C	10.374365000	-0.612394000	-0.080450000
S	9.406765000	3.441734000	-2.540415000
C	8.101723000	4.044269000	-3.659050000
H	-4.436819000	-0.312749000	1.914151000
H	-6.107266000	0.178298000	-2.874153000
H	2.694273000	5.575128000	4.314474000
H	6.755569000	4.196813000	4.078266000
H	7.563636000	6.561998000	3.995677000
H	3.484644000	7.947123000	4.234479000
H	8.853041000	-2.957398000	0.710020000
H	4.563417000	-5.319561000	1.781003000
H	4.847733000	1.010083000	-5.907256000
H	8.996271000	0.321216000	-3.163035000
H	0.198042000	-4.214430000	-0.504186000
H	-1.448292000	-2.953256000	-4.309583000
H	0.490407000	-1.419695000	-4.690056000
H	4.832543000	-2.512960000	3.577761000
H	0.095263000	-0.927904000	3.132892000
H	6.974665000	4.485947000	-1.253251000
H	8.442312000	0.362685000	1.196230000
H	-1.190569000	3.514415000	1.508611000
H	-0.200266000	6.631741000	-1.326166000
H	2.232454000	6.351255000	-0.836696000
H	4.171028000	2.565487000	-1.563685000
H	1.165652000	0.156068000	0.332857000

H	-0.622165000	1.413605000	-0.880492000
H	2.397991000	3.827219000	-2.787626000
H	10.487797000	-0.509437000	1.008462000
H	11.231278000	-1.176220000	-0.474607000
H	9.436433000	-1.124044000	-0.335048000
H	5.925043000	8.442589000	4.070161000
H	11.946149000	0.940122000	-5.144825000
H	10.848201000	-0.361835000	-4.584375000
H	10.911915000	1.212350000	-3.703795000
H	-0.002942000	3.236108000	-2.465063000
H	10.572353000	-3.337384000	2.196425000
H	10.765298000	-4.108174000	0.578333000
H	11.684401000	-4.724130000	1.983414000
H	-2.220319000	-3.659958000	3.201395000
H	-1.400616000	-2.460403000	2.161273000
H	-1.539037000	-2.188713000	3.939094000
H	5.434530000	-4.537255000	4.257081000
H	4.715367000	-5.734465000	5.361503000
H	4.533723000	-3.985118000	5.721356000
H	5.014194000	1.078076000	-8.162879000
H	5.633339000	2.477157000	-9.089064000
H	4.974325000	2.730707000	-7.443841000
H	5.257371000	-8.695083000	2.882248000
H	5.000967000	-7.747347000	1.381768000
H	4.407991000	-7.121046000	2.960440000
H	8.559681000	4.068178000	-4.657181000
H	7.754969000	5.050382000	-3.385397000
H	7.263404000	3.334984000	-3.671824000

***syn*-SubPc 1-C_{60}**
278

B	-2.996238000	-5.183890000	1.939767000
N	-3.442196000	-3.895194000	1.285469000
C	-3.858921000	-3.846525000	-0.017770000
N	-4.158962000	-4.944471000	-0.717590000
C	-4.231525000	-6.104521000	-0.054403000
N	-3.841130000	-6.227503000	1.247354000
C	-4.968491000	-7.329343000	-0.345809000
C	-5.087279000	-8.056625000	0.888618000
C	-4.420490000	-7.267649000	1.918652000
C	-3.707525000	-2.757362000	1.993524000
C	-4.150870000	-1.776267000	1.012080000
C	-4.211543000	-2.452272000	-0.264810000

N	-4.529603000	-7.290008000	3.250316000
N	-3.797232000	-2.741067000	3.327509000
C	-3.827047000	-3.918705000	3.960942000
N	-3.613703000	-5.103637000	3.317247000
C	-4.201094000	-6.182990000	3.921268000
C	-4.387953000	-4.281876000	5.255539000
C	-4.625488000	-5.698827000	5.231024000
C	-5.595155000	-7.796621000	-1.500806000
C	-6.318603000	-8.989253000	-1.437379000
C	-6.435084000	-9.701530000	-0.228724000
C	-5.830527000	-9.235852000	0.940955000
C	-4.594468000	-0.465017000	1.168669000
C	-4.641158000	-1.801649000	-1.419665000
C	-5.255217000	-6.321622000	6.309026000
C	-5.625791000	-5.546517000	7.410319000
C	-5.391577000	-4.157899000	7.433543000
C	-4.782189000	-3.518081000	6.353244000
F	-6.907210000	-9.470495000	-2.524344000
F	-7.129708000	-10.831301000	-0.215513000
F	-6.205050000	-6.114573000	8.459370000
B	-6.241661000	2.924131000	-2.159141000
N	-5.728411000	1.648548000	-1.537130000
C	-5.524570000	0.500610000	-2.244157000
N	-6.007674000	0.330278000	-3.479197000
C	-6.846070000	1.257167000	-3.953479000
N	-7.090221000	2.440310000	-3.307363000
C	-7.895276000	1.149767000	-4.960514000
C	-8.817313000	2.226196000	-4.729117000
C	-8.321072000	2.978370000	-3.582585000
C	-5.595177000	1.510401000	-0.184904000
C	-5.004636000	0.191525000	0.011763000
C	-4.992675000	-0.459648000	-1.281393000
N	-8.967274000	3.782088000	-2.733801000
N	-6.197946000	2.330966000	0.682111000
C	-7.089734000	3.201352000	0.193000000
N	-7.257890000	3.398022000	-1.146763000
C	-8.466818000	3.920225000	-1.502201000
C	-8.227590000	3.868920000	0.816265000
C	-9.090119000	4.317274000	-0.243820000
C	-8.162241000	0.191434000	-5.938462000
C	-9.327518000	0.313644000	-6.697455000
C	-10.231566000	1.369100000	-6.470802000
C	-9.988698000	2.323217000	-5.480721000
C	-10.313895000	4.917124000	0.052427000

C	-10.672021000	5.088591000	1.391569000
C	-9.828082000	4.649196000	2.428845000
C	-8.608855000	4.028827000	2.148035000
F	-9.598287000	-0.568305000	-7.650599000
F	-11.820033000	5.674678000	1.704405000
F	-10.207446000	4.834808000	3.686245000
F	-7.843263000	3.615692000	3.149100000
F	-11.138101000	5.332160000	-0.898757000
F	-7.338642000	-0.821771000	-6.169050000
F	-5.515186000	-7.144643000	-2.652823000
F	-5.970652000	-9.925441000	2.063861000
F	-5.502030000	-7.623334000	6.318714000
F	-5.757331000	-3.463396000	8.502270000
F	-4.584401000	-2.204816000	6.398607000
C	-1.457175000	-5.449617000	1.887587000
C	-5.092444000	3.965971000	-2.374916000
C	-4.137008000	4.681697000	-2.117058000
C	-0.252665000	-5.616562000	1.789183000
B	6.447225000	-5.415634000	-1.312295000
N	7.107573000	-4.174995000	-0.742700000
C	7.528279000	-3.131495000	-1.530637000
N	7.221328000	-3.041403000	-2.828085000
C	6.325038000	-3.899305000	-3.325163000
N	5.829785000	-4.954681000	-2.601377000
C	5.408005000	-3.716318000	-4.440901000
C	4.304948000	-4.593670000	-4.227041000
C	4.559418000	-5.310592000	-2.985221000
C	7.024869000	-3.834528000	0.583957000
C	7.640239000	-2.526088000	0.703437000
C	7.950429000	-2.078837000	-0.612706000
N	3.684761000	-5.869122000	-2.145870000
N	6.188072000	-4.426237000	1.434655000
C	5.267226000	-5.249081000	0.927551000
N	5.265032000	-5.639792000	-0.386673000
C	4.011056000	-5.943048000	-0.851523000
C	3.936584000	-5.523149000	1.433819000
C	3.140493000	-5.940234000	0.320077000
C	0.015808000	10.043764000	1.989230000
C	1.102691000	9.410204000	2.608097000
C	2.384772000	9.967926000	2.501589000
C	2.574319000	11.147343000	1.778542000
C	1.492387000	11.778437000	1.157225000
C	0.214022000	11.223236000	1.268486000
O	0.914633000	8.251100000	3.301881000

C	5.348645000	-2.731219000	-5.428453000
C	4.187372000	-2.597672000	-6.191824000
C	3.071965000	-3.477872000	-5.971167000
C	3.146746000	-4.475156000	-4.996467000
C	7.675351000	-1.648188000	1.786315000
C	7.988636000	-0.309766000	1.563368000
C	8.296449000	0.152915000	0.237797000
C	8.295229000	-0.742531000	-0.836090000
C	1.753387000	-6.024778000	0.437929000
C	1.163028000	-5.703954000	1.671971000
C	1.970996000	-5.339401000	2.785675000
C	3.348254000	-5.236812000	2.670847000
B	1.049998000	7.014183000	2.589355000
N	2.398058000	6.769904000	1.956096000
C	3.391097000	6.064516000	2.586336000
N	3.146817000	5.299527000	3.655017000
C	1.870859000	5.111469000	4.019554000
N	0.831105000	5.821979000	3.473928000
C	1.260044000	3.963067000	4.676543000
C	-0.132794000	3.986764000	4.369623000
C	-0.363958000	5.146260000	3.518196000
C	2.659392000	7.008954000	0.632869000
C	4.046042000	6.623285000	0.431347000
C	4.520346000	6.067258000	1.657467000
N	-1.343117000	5.357308000	2.633544000
N	1.697283000	7.238319000	-0.261115000
C	0.431180000	7.063996000	0.130522000
N	0.090228000	6.819786000	1.436365000
C	-1.080550000	6.123042000	1.570341000
C	-0.731896000	6.697661000	-0.659176000
C	-1.675159000	6.098525000	0.238313000
C	1.835708000	2.824556000	5.246325000
C	1.041189000	1.704049000	5.491722000
C	-0.364713000	1.727776000	5.184763000
C	-0.940635000	2.879484000	4.643473000
C	4.824269000	6.568259000	-0.724084000
C	6.113933000	6.039881000	-0.651194000
C	6.642363000	5.602901000	0.609316000
C	5.820267000	5.564549000	1.741532000
C	-2.808503000	5.458418000	-0.251040000
C	-3.019959000	5.427099000	-1.641980000
C	-2.096597000	6.050888000	-2.522969000
C	-0.950913000	6.671861000	-2.041216000
C	9.185349000	-6.582666000	-0.010231000

C	7.929382000	-7.067003000	-0.402554000
C	7.313474000	-8.083653000	0.341073000
C	7.950133000	-8.606638000	1.468484000
C	9.200565000	-8.121969000	1.863923000
C	9.814984000	-7.111124000	1.118571000
O	7.305972000	-6.547298000	-1.498660000
F	-10.867107000	3.296078000	-5.285860000
F	-11.324597000	1.447211000	-7.218030000
S	3.965886000	-1.304115000	-7.381292000
C	5.424010000	-0.259335000	-7.114452000
S	1.598318000	-3.152256000	-6.895795000
C	0.391730000	-4.276553000	-6.140624000
S	8.515568000	1.897498000	0.053063000
C	8.626249000	2.122400000	-1.739438000
S	7.938791000	0.894229000	2.867181000
C	6.904598000	0.079903000	4.119181000
S	1.707953000	0.165404000	6.063315000
C	3.493655000	0.480357000	6.072362000
S	-1.259254000	0.214946000	5.411959000
C	-2.852645000	0.546729000	4.612147000
S	8.353237000	5.158318000	0.638472000
C	8.570467000	4.498436000	2.310112000
S	7.153030000	5.857381000	-2.079068000
C	5.986276000	6.110389000	-3.446885000
H	-4.615887000	0.023211000	2.142946000
H	-4.697535000	-2.313335000	-2.381159000
H	-0.976355000	9.598770000	2.085340000
H	3.220050000	9.465028000	2.992447000
H	3.576683000	11.575858000	1.699795000
H	-0.638219000	11.711581000	0.789132000
H	6.185866000	-2.045178000	-5.538554000
H	2.299053000	-5.119657000	-4.773684000
H	7.387256000	-2.014339000	2.769881000
H	8.472410000	-0.402890000	-1.854757000
H	1.134001000	-6.265799000	-0.426336000
H	1.475925000	-5.090502000	3.725428000
H	3.963372000	-4.894435000	3.504418000
H	2.910102000	2.810240000	5.416597000
H	-1.989048000	2.906101000	4.352036000
H	4.400147000	6.923268000	-1.661241000
H	6.176051000	5.157703000	2.685945000
H	-3.495788000	4.947140000	0.423898000
H	-2.284164000	6.000429000	-3.596566000
H	-0.215960000	7.105732000	-2.721017000

H	9.653638000	-5.794443000	-0.602578000
H	6.338015000	-8.453122000	0.019281000
H	7.464068000	-9.399522000	2.042645000
H	10.794242000	-6.728904000	1.417840000
H	9.601653000	4.122848000	2.342572000
H	7.888361000	3.656047000	2.483788000
H	8.439785000	5.281138000	3.070732000
H	1.644249000	12.700490000	0.591641000
H	7.739650000	1.709559000	-2.240283000
H	9.545388000	1.682800000	-2.152218000
H	8.631368000	3.211383000	-1.879900000
H	9.695609000	-8.532745000	2.746725000
H	6.355514000	-0.779391000	-7.379756000
H	5.467513000	0.100945000	-6.076230000
H	5.290962000	0.598075000	-7.788300000
H	-3.384294000	-0.415014000	4.626167000
H	-2.702548000	0.858010000	3.568539000
H	-3.436144000	1.300905000	5.158983000
H	3.849085000	0.756461000	5.069295000
H	3.953832000	-0.472245000	6.368800000
H	3.769610000	1.253360000	6.803902000
H	6.702213000	0.850677000	4.875578000
H	7.421630000	-0.761844000	4.601188000
H	5.955812000	-0.256300000	3.677691000
H	-0.558819000	-4.061158000	-6.647391000
H	0.280339000	-4.074811000	-5.065653000
H	0.654697000	-5.330818000	-6.307444000
H	6.553105000	5.879105000	-4.359114000
H	5.636155000	7.150957000	-3.501416000
H	5.131133000	5.423999000	-3.365785000
C	1.635141000	4.013299000	-0.274843000
C	2.593363000	3.604784000	0.655493000
C	3.903568000	3.152720000	0.209608000
C	4.316851000	2.054944000	1.075728000
C	3.263342000	1.832656000	2.058764000
C	2.199105000	2.789012000	1.793090000
C	0.866456000	2.409177000	1.948327000
C	0.534715000	1.061033000	2.380508000
C	-0.672626000	0.650609000	1.677812000
C	-0.806710000	-0.670607000	1.242702000
C	-1.355401000	-0.943239000	-0.078925000
C	-0.638648000	-2.081552000	-0.637676000
C	0.359651000	-2.505406000	0.336132000
C	0.255149000	-1.636477000	1.499768000

C	1.410971000	-1.238476000	2.180213000
C	1.551361000	0.137925000	2.633013000
C	2.944236000	0.532073000	2.466116000
C	3.666970000	-0.604377000	1.907087000
C	2.718455000	-1.698052000	1.730053000
C	2.817755000	-2.534094000	0.614871000
C	1.614095000	-2.944132000	-0.096960000
C	1.921730000	-2.978545000	-1.520592000
C	0.962462000	-2.575221000	-2.455823000
C	-0.345599000	-2.116344000	-2.004716000
C	-0.758792000	-1.015923000	-2.868054000
C	-1.449639000	0.074573000	-2.329930000
C	-1.755171000	0.110728000	-0.906470000
C	-1.620875000	1.487656000	-0.450792000
C	-1.089551000	1.751843000	0.815589000
C	-0.135117000	2.838132000	0.986195000
C	0.243454000	3.619764000	-0.106993000
C	-0.310257000	3.344325000	-1.423749000
C	-1.224208000	2.302153000	-1.593306000
C	-1.120675000	1.428954000	-2.754888000
C	-0.109883000	1.639133000	-3.698688000
C	0.610866000	0.505227000	-4.258474000
C	0.293341000	-0.794529000	-3.850839000
C	1.356416000	-1.753994000	-3.592080000
C	2.691067000	-1.376040000	-3.749515000
C	3.686394000	-1.797598000	-2.777720000
C	3.312794000	-2.585376000	-1.688030000
C	3.869940000	-2.312550000	-0.369166000
C	4.776842000	-1.262957000	-0.200531000
C	4.674781000	-0.389839000	0.960845000
C	5.003767000	0.965216000	0.537675000
C	5.305895000	0.931276000	-0.885773000
C	5.165177000	-0.443542000	-1.342072000
C	4.639251000	-0.710283000	-2.606444000
C	4.229868000	0.386926000	-3.474245000
C	3.023224000	-0.026620000	-4.180791000
C	2.004193000	0.896643000	-4.427250000
C	2.145617000	2.272575000	-3.971474000
C	0.838610000	2.732175000	-3.522241000
C	0.740373000	3.568592000	-2.407164000
C	1.943271000	3.985315000	-1.697369000
C	3.199649000	3.547682000	-2.128846000
C	3.301941000	2.672512000	-3.290911000
C	4.364923000	1.709169000	-3.035662000

C	4.918131000	1.987375000	-1.715158000
C	4.201151000	3.124955000	-1.155094000

***syn*-SubPc 1-C_{70}**

288

B	-4.568743000	-4.681081000	1.851450000
N	-4.686196000	-3.272432000	1.314950000
C	-4.756472000	-3.001307000	-0.024025000
N	-4.990351000	-3.947655000	-0.937924000
C	-5.350005000	-5.159035000	-0.498798000
N	-5.317700000	-5.498019000	0.823284000
C	-6.115384000	-6.220503000	-1.143177000
C	-6.623455000	-7.069881000	-0.100699000
C	-6.160270000	-6.517649000	1.167553000
C	-4.969826000	-2.200171000	2.113414000
C	-5.001882000	-1.045353000	1.222959000
C	-4.859379000	-1.549549000	-0.124937000
N	-6.611901000	-6.683825000	2.413850000
N	-5.405189000	-2.334056000	3.369880000
C	-5.745522000	-3.559919000	3.782242000
N	-5.504699000	-4.679839000	3.037053000
C	-6.348427000	-5.724176000	3.304704000
C	-6.672482000	-3.987906000	4.823628000
C	-7.049794000	-5.341745000	4.524914000
C	-6.471977000	-6.447635000	-2.472231000
C	-7.311727000	-7.522905000	-2.768572000
C	-7.810013000	-8.354519000	-1.747712000
C	-7.478313000	-8.127890000	-0.410232000
C	-5.231110000	0.304783000	1.480507000
C	-4.920947000	-0.707182000	-1.232978000
C	-8.011233000	-5.990915000	5.299833000
C	-8.578602000	-5.307546000	6.377321000
C	-8.208631000	-3.980919000	6.669888000
C	-7.263574000	-3.311262000	5.890560000
F	-7.650273000	-7.777874000	-4.025765000
F	-8.602508000	-9.366339000	-2.075691000
F	-9.478468000	-5.905669000	7.146649000
B	-5.381666000	4.303282000	-1.580005000
N	-5.275166000	2.892010000	-1.051920000
C	-5.174726000	1.800078000	-1.866054000
N	-5.419693000	1.860816000	-3.178726000
C	-5.947242000	2.990975000	-3.660386000
N	-6.081041000	4.124340000	-2.906208000

C	-6.765583000	3.223999000	-4.844908000
C	-7.476819000	4.454238000	-4.633839000
C	-7.084198000	4.958209000	-3.322641000
C	-5.477191000	2.589373000	0.265617000
C	-5.237730000	1.154139000	0.375688000
C	-5.070195000	0.653574000	-0.970396000
N	-7.700068000	5.812214000	-2.500845000
N	-6.063048000	3.439120000	1.114801000
C	-6.619069000	4.543334000	0.602511000
N	-6.451967000	4.909743000	-0.702125000
C	-7.434366000	5.721467000	-1.194407000
C	-7.700444000	5.388498000	1.096687000
C	-8.211186000	6.124930000	-0.027449000
C	-7.012567000	2.453644000	-5.981245000
C	-7.944622000	2.912509000	-6.913885000
C	-8.641662000	4.118245000	-6.707332000
C	-8.420910000	4.889250000	-5.564249000
C	-9.322523000	6.954645000	0.122050000
C	-9.911185000	7.068719000	1.383262000
C	-9.411005000	6.347469000	2.483934000
C	-8.312101000	5.497378000	2.345391000
F	-8.184850000	2.214120000	-8.015948000
F	-10.955636000	7.866770000	1.561635000
F	-10.000042000	6.488863000	3.664194000
F	-7.873658000	4.823581000	3.400353000
F	-9.826615000	7.639156000	-0.894711000
F	-6.382674000	1.307567000	-6.200474000
F	-6.028797000	-5.677344000	-3.457127000
F	-7.974465000	-8.923659000	0.526144000
F	-8.391728000	-7.234703000	5.046008000
F	-8.771754000	-3.371951000	7.705236000
F	-6.946296000	-2.057822000	6.186276000
C	-3.100954000	-5.162857000	2.103612000
C	-4.024013000	5.082599000	-1.569377000
C	-2.938648000	5.612560000	-1.394836000
C	-1.930915000	-5.505205000	2.157522000
B	4.705889000	-6.799121000	-0.798846000
N	5.643717000	-5.717336000	-0.312154000
C	6.328653000	-4.901672000	-1.176058000
N	6.068208000	-4.854803000	-2.486705000
C	4.979332000	-5.487101000	-2.933395000
N	4.226398000	-6.309769000	-2.134697000
C	4.143061000	-5.179735000	-4.085993000
C	2.848585000	-5.714354000	-3.819167000

C	2.909347000	-6.364612000	-2.517323000
C	5.638201000	-5.247260000	0.978637000
C	6.576814000	-4.134893000	0.998759000
C	7.006408000	-3.918912000	-0.342759000
N	1.923346000	-6.644552000	-1.662016000
N	4.676966000	-5.551840000	1.853300000
C	3.591953000	-6.185157000	1.396783000
N	3.504833000	-6.652759000	0.109465000
C	2.221110000	-6.709277000	-0.360254000
C	2.227373000	-6.160384000	1.901730000
C	1.368888000	-6.469266000	0.796927000
C	3.004030000	10.123022000	1.486664000
C	3.956668000	9.268681000	2.059632000
C	5.312135000	9.422118000	1.735063000
C	5.706679000	10.420262000	0.841890000
C	4.757697000	11.270474000	0.266160000
C	3.407185000	11.118983000	0.594791000
O	3.564903000	8.286191000	2.920676000
C	4.348672000	-4.332682000	-5.176574000
C	3.268425000	-3.997797000	-5.995896000
C	1.965032000	-4.539351000	-5.729634000
C	1.768211000	-5.394791000	-4.643843000
C	6.857049000	-3.193571000	1.992011000
C	7.543902000	-2.027977000	1.651719000
C	7.971223000	-1.805762000	0.296383000
C	7.710561000	-2.763580000	-0.685037000
C	-0.007889000	-6.297418000	0.894886000
C	-0.545557000	-5.833271000	2.108066000
C	0.306177000	-5.586843000	3.220045000
C	1.683509000	-5.732776000	3.119811000
B	3.254240000	6.998177000	2.371396000
N	4.376394000	6.319573000	1.626386000
C	5.223789000	5.428038000	2.229707000
N	4.951904000	4.877435000	3.416745000
C	3.745151000	5.102022000	3.951203000
N	2.860858000	6.011078000	3.429231000
C	2.962291000	4.259108000	4.844179000
C	1.595811000	4.636627000	4.692034000
C	1.550853000	5.704999000	3.703208000
C	4.500064000	6.349955000	0.261886000
C	5.694118000	5.571167000	-0.038144000
C	6.164005000	5.020801000	1.192169000
N	0.542862000	6.083062000	2.912385000
N	3.513090000	6.761890000	-0.535617000

C	2.317493000	6.993013000	0.018603000
N	2.117909000	6.971648000	1.375865000
C	0.839277000	6.637727000	1.732286000
C	0.994085000	6.896650000	-0.576010000
C	0.071809000	6.650905000	0.492440000
C	3.301370000	3.096861000	5.540200000
C	2.288775000	2.293350000	6.066405000
C	0.911607000	2.678891000	5.920383000
C	0.579765000	3.854336000	5.244705000
C	6.277075000	5.193323000	-1.247967000
C	7.374269000	4.329208000	-1.235357000
C	7.926316000	3.881130000	0.011866000
C	7.279613000	4.183858000	1.213165000
C	-1.238387000	6.265319000	0.223776000
C	-1.645964000	6.142720000	-1.116162000
C	-0.740863000	6.447224000	-2.169898000
C	0.573900000	6.808658000	-1.908421000
C	7.064553000	-8.486250000	0.662294000
C	5.731202000	-8.681453000	0.275016000
C	4.879509000	-9.451352000	1.079944000
C	5.360224000	-10.017064000	2.262632000
C	6.688348000	-9.820093000	2.652674000
C	7.537162000	-9.055520000	1.846545000
O	5.261640000	-8.118499000	-0.875057000
F	-9.103589000	6.012666000	-5.396116000
F	-9.516003000	4.517007000	-7.621700000
S	3.387695000	-2.803077000	-7.298008000
C	5.118292000	-2.270737000	-7.197560000
S	0.637333000	-3.988454000	-6.765624000
C	-0.835605000	-4.514242000	-5.844852000
S	8.713008000	-0.241321000	-0.057218000
C	8.788169000	-0.218623000	-1.866346000
S	7.857737000	-0.727855000	2.816979000
C	7.009905000	-1.307771000	4.310767000
S	2.608658000	0.730659000	6.840578000
C	4.364416000	0.463338000	6.479338000
S	-0.309699000	1.541352000	6.510427000
C	-1.827054000	2.129268000	5.706891000
S	9.452316000	2.987145000	-0.057443000
C	9.700547000	2.484189000	1.665374000
S	8.119399000	3.698421000	-2.715440000
C	7.093207000	4.419849000	-4.025681000
H	-5.385216000	0.677733000	2.493740000
H	-4.834449000	-1.089104000	-2.250630000

H	1.953457000	9.992894000	1.753106000
H	6.042137000	8.751865000	2.192686000
H	6.765037000	10.533513000	0.593641000
H	2.658779000	11.781393000	0.152450000
H	5.333394000	-3.894269000	-5.323352000
H	0.781783000	-5.780749000	-4.394625000
H	6.472756000	-3.360160000	2.995964000
H	7.978954000	-2.596957000	-1.726316000
H	-0.651454000	-6.457859000	0.029679000
H	-0.140800000	-5.225169000	4.147192000
H	2.338277000	-5.480687000	3.955265000
H	4.347688000	2.803263000	5.591876000
H	-0.456467000	4.139323000	5.074352000
H	5.845349000	5.558074000	-2.177341000
H	7.631211000	3.789972000	2.164349000
H	-1.918182000	6.002241000	1.034759000
H	-1.085752000	6.339461000	-3.199163000
H	1.283994000	6.975202000	-2.719925000
H	7.715956000	-7.888089000	0.022472000
H	3.845545000	-9.597674000	0.761925000
H	4.690414000	-10.616452000	2.884454000
H	8.577961000	-8.899485000	2.141505000
H	10.597892000	1.851788000	1.656390000
H	8.853391000	1.882896000	2.021127000
H	9.869691000	3.354575000	2.314938000
H	5.069568000	12.049770000	-0.432814000
H	7.793359000	-0.383619000	-2.301946000
H	9.508247000	-0.953162000	-2.253630000
H	9.117363000	0.797651000	-2.116408000
H	7.061135000	-10.263453000	3.578746000
H	5.812233000	-3.098633000	-7.401674000
H	5.336849000	-1.816624000	-6.220435000
H	5.230519000	-1.508356000	-7.980553000
H	-2.591689000	1.378871000	5.949905000
H	-1.701630000	2.174652000	4.615860000
H	-2.149438000	3.104975000	6.096884000
H	4.542909000	0.477617000	5.394118000
H	4.591808000	-0.538134000	6.869861000
H	5.007587000	1.197640000	6.985176000
H	7.148888000	-0.507750000	5.050661000
H	7.456198000	-2.234761000	4.697942000
H	5.935312000	-1.446303000	4.125938000
H	-1.687035000	-4.095958000	-6.398825000
H	-0.830968000	-4.102346000	-4.825644000

H	-0.933880000	-5.608748000	-5.816069000
H	7.501834000	4.022036000	-4.964563000
H	7.166458000	5.516581000	-4.039171000
H	6.044769000	4.105577000	-3.928443000
C	0.429324000	-3.117426000	0.896915000
C	1.485344000	-1.602167000	3.071721000
C	-0.449737000	-2.274330000	1.686732000
C	0.064783000	-1.526519000	2.751653000
C	0.001880000	-3.063568000	-0.490056000
C	0.934024000	-3.172727000	-1.559032000
C	1.782629000	-3.187678000	1.200294000
C	2.324343000	-2.423226000	2.310085000
C	1.933622000	-0.272061000	3.455807000
C	0.791615000	0.625752000	3.366733000
C	-1.404978000	-1.681136000	0.767527000
C	-0.362163000	-0.147218000	2.934400000
C	-1.128546000	-2.176535000	-0.570711000
C	2.368146000	-3.246980000	-1.236196000
C	0.546974000	-2.582238000	-2.795100000
C	2.762521000	-3.208303000	0.129457000
C	3.634986000	-1.952437000	1.899676000
C	3.201855000	0.175776000	3.060924000
C	0.970775000	1.926018000	2.888635000
C	-1.811646000	-0.361557000	0.944591000
C	-1.291470000	0.419976000	2.052661000
C	-1.348264000	-1.369639000	-1.723348000
C	3.303635000	-2.724128000	-2.170585000
C	-0.581828000	-1.694181000	-2.878310000
C	2.881823000	-2.210190000	-3.459157000
C	1.531212000	-2.142053000	-3.768327000
C	3.899773000	-2.440597000	0.557666000
C	4.065904000	-0.680355000	2.266401000
C	3.378174000	1.529024000	2.560974000
C	2.285446000	2.383119000	2.477498000
C	0.010924000	2.510601000	1.969467000
C	-1.950849000	0.509755000	-0.208880000
C	-1.096448000	1.774457000	1.559247000
C	4.669101000	-1.685520000	-0.367336000
C	4.784562000	0.145709000	1.308962000
C	4.360765000	1.507618000	1.491884000
C	2.134095000	3.254749000	1.326930000
C	0.732230000	3.328387000	1.010853000
C	-1.772656000	0.025706000	-1.534436000
C	-1.517524000	1.826383000	0.169242000

C	4.442232000	-1.960216000	-1.742679000
C	5.130341000	-0.346647000	0.020843000
C	4.283843000	2.410689000	0.393494000
C	3.126063000	3.314501000	0.308227000
C	0.285940000	3.462747000	-0.331489000
C	-0.882976000	2.687348000	-0.769880000
C	4.738797000	-0.977991000	-2.770323000
C	5.326914000	0.627633000	-0.996993000
C	4.907762000	1.989748000	-0.814317000
C	2.678412000	3.728958000	-0.976374000
C	1.279277000	3.799749000	-1.290675000
C	-1.413208000	0.991078000	-2.515635000
C	-0.299075000	-0.705553000	-3.906700000
C	-0.972957000	2.307174000	-2.137557000
C	5.170942000	0.293913000	-2.403964000
C	4.483170000	2.503039000	-2.106509000
C	3.778281000	-1.137977000	-3.849091000
C	3.391421000	3.360716000	-2.186056000
C	1.115657000	3.480077000	-2.696695000
C	4.660898000	1.460943000	-3.103327000
C	1.006382000	-0.989433000	-4.475456000
C	3.278445000	-0.019024000	-4.523703000
C	1.859317000	0.057368000	-4.843610000
C	-0.708668000	0.612592000	-3.729156000
C	2.426347000	3.216164000	-3.263359000
C	0.010676000	2.745436000	-3.113341000
C	3.728593000	1.310640000	-4.139018000
C	2.586037000	2.209359000	-4.221568000
C	1.431779000	1.434248000	-4.655419000
C	0.168702000	1.703604000	-4.113018000

8. References

1. D. S. Terekhov, K. J. M. Nolan, C. R. McArthur and C. C. Leznoff, *J. Org. Chem.*, 1996, **61**, 3034.
2. D. Wöhrle, M. Eskes, K. Shigehara and A. Yamada, *Synthesis*, 1993, 194.
3. B. del Rey, U. Keller, T. Torres, G. Rojo, F. Agullo-López, S. Nonell, C. Marti, S. Brasselet, I. Ledoux and J. Zyss, *J. Am. Chem. Soc*., 1998, **120**, 12808.
4. R. S. Iglesias, C. G. Claessens, T. Torres, M.A. Herranz, V. R. Ferro and J. M. Garcia de la Vega, *J. Org. Chem.*, 2007, **72**, 2967.
5. J. Snellenburg, S. P. Laptenok, R. Seger, K. M. Mullen, I. H. M. van Stokkum, *J. Stat. Softw.* 2012, **49**, 1–22.
6. Gaussian 16: M. J. Frisch, G. W. Trucks, H. B. Schlegel, G. E. Scuseria, M. A. Robb, J. R. Cheeseman, G. Scalmani, V. Barone, G. A. Petersson, H. Nakatsuji, X. Li, M. Caricato, A. V. Marenich, J. Bloino, B. G. Janesko, R. Gomperts, B. Mennucci, H. P. Hratchian, J. V. Ortiz, A. F. Izmaylov, J. L. Sonnenberg, D. Williams-Young, F. Ding, F. Lipparini, F. Egidi, J. Goings, B. Peng, A. Petrone, T. Henderson, D. Ranasinghe, V. G. Zakrzewski, J. Gao, N. Rega, G. Zheng, W. Liang, M. Hada, M. Ehara, K. Toyota, R. Fukuda, J. Hasegawa, M. Ishida, T. Nakajima, Y. Honda, O. Kitao, H. Nakai, T. Vreven, K. Throssell, J. A. Montgomery, Jr., J. E. Peralta, F. Ogliaro, M. J. Bearpark, J. J. Heyd, E. N. Brothers, K. N. Kudin, V. N. Staroverov, T. A. Keith, R. Kobayashi, J. Normand, K. Raghavachari, A. P. Rendell, J. C. Burant, S. S. Iyengar, J. Tomasi, M. Cossi, J. M. Millam, M. Klene, C. Adamo, R. Cammi, J. W. Ochterski, R. L. Martin, K. Morokuma, O. Farkas, J. B. Foresman, and D. J. Fox, Gaussian, Inc., Wallingford CT, 2016.
7. A. D. Becke, J. Chem. Phys., 1993, **98**, 5648.
8. F. Weigend and R. Ahlrichs, Phys. Chem. Chem. Phys., 2005, **7**, 3297.
9. F. Weigend, Phys. Chem. Chem. Phys., 2006, **8**, 1057.
10. S. Grimme, S. Ehrlich and L. Goerigk, J. Comput. Chem., 2011, **32**, 1456.
